# Prebiotic synthesis of the major classes of iron–sulfur clusters†

**DOI:** 10.1039/d5sc00524h

**Published:** 2025-02-11

**Authors:** Simone Scintilla, Daniele Rossetto, Martin Clémancey, Julia Rendon, Antonio Ranieri, Graziano Guella, Michael Assfalg, Marco Borsari, Serge Gambarelli, Geneviève Blondin, Sheref S. Mansy

**Affiliations:** a DiCIBIO, University of Trento Via Sommarive 9 Povo TN 38123 Italy; b Hudson River, Department of Biochemistry Nieuwe Kanaal 7V Wageningen PA 6709 Netherlands simone.scintilla@hudsonriverbiotechnology.com; c Univ. Grenoble Alpes, CNRS, CEA, IRIG, Laboratoire de Chimie et Biologie des Métaux – UMR 5249 17 rue des Martyrs Grenoble 38000 France; d CEA, Laboratoire de Résonance Magnétique, INAC/SCIB, UMR E3 CEA-UJF 17, rue des Martyrs Grenoble Cedex 9 38054 France; e University of Grenoble Alpes, CNRS, CEA, INAC-SyMMES Grenoble 38000 France; f Department of Life Sciences, University of Modena and Reggio Emilia Via G. Campi, 103 Modena 41125 Italy; g Department of Physics, University of Trento Via Sommarive 14 Povo TN 38123 Italy; h Department of Biotechnology, University of Verona Strada Le Grazie 15 Verona 37134 Italy; i Department of Chemical and Geological Sciences, University of Modena and Reggio Emilia Via G. Campi, 103 Modena 41125 Italy; j Department of Chemistry, University of Alberta 11227 Saskatchewan Drive Edmonton AB T6G 2G2 Canada sheref.mansy@ualberta.ca

## Abstract

Conditions that led to the synthesis of iron–sulfur clusters coordinated to tripeptides with a single thiolate ligand were investigated by UV-vis, NMR, EPR, and Mössbauer spectroscopies and by electrochemistry. Increasing concentrations of hydrosulfide correlated with the formation of higher nuclearity iron–sulfur clusters from mononuclear to [2Fe–2S] to [4Fe–4S] and finally to a putative, nitrogenase-like [6Fe–9S] complex. Increased nuclearity was also associated with decreased dynamics and increased stability. The synthesis of higher nuclearity iron–sulfur clusters is compatible with shallow, alkaline bodies of water on the surface of the early Earth, although other niche environments are possible. Because of the plasticity of such complexes, the type of iron–sulfur cluster formed on the prebiotic Earth would have been greatly influenced by the chemical environment and the thiolate containing scaffold. The discovery that all the major classes of iron–sulfur clusters easily form under prebiotically reasonable conditions broadens the chemistry accessible to protometabolic systems.

## Abstract 2

Greater concentrations of hydrosulfide lead to the prebiotic formation of higher nuclearity Fe–S peptides, culminating in a putative nitrogenase-like [6Fe–9S] cluster. Higher nuclearity clusters are more stable with lower reduction potential.

## Graphical abstract

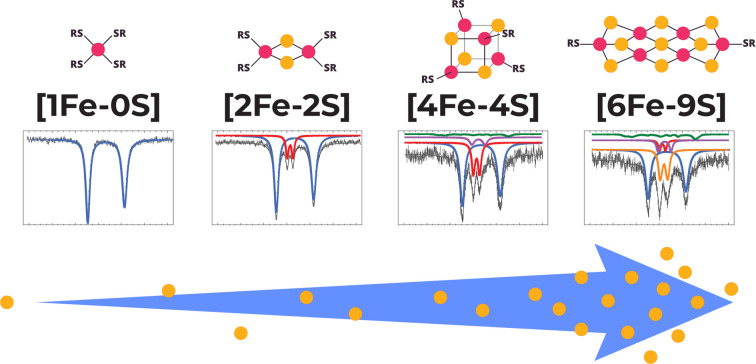

## Introduction

In biological systems, iron–sulfur clusters are sensed, trafficked, and synthesized by specialized protein machinery.^1–5^ The prebiotic assembly of iron–sulfur clusters must have been largely different. Since metallochaperone proteins were not present, iron–sulfur clusters were likely assembled through stochastic encounters with free iron ions and hydrosulfide (HS^−^) in the environment.^3^ The three main types of iron–sulfur complexes, *i.e.* mononuclear, [2Fe–2S], and [4Fe–4S] clusters, are usually coordinated to a single polypeptide by four cysteinyl thiolate side-chains. In biology, different sequence motifs typically coordinate different types of iron–sulfur clusters and are not normally capable of binding more than one type of iron–sulfur cluster.

Conversely, model prebiotic peptides containing a single cysteine are capable of coordinating different types of iron–sulfur clusters, depending upon the solution conditions.^6,7^ This is because the positioning of the cysteinyl ligands are not constrained, since each of the four cysteines comes from four different peptides. Such a dynamic system suggests that the type of iron–sulfur cluster stabilized by peptides on the prebiotic Earth was dictated by the encountered environmental conditions. To gain insight into the influencing factors on the synthesis of iron–sulfur clusters, we investigated the mechanism of cluster assembly on the tripeptide glutathione (EγCG). Glutathione is a readily available, prebiotically reasonable analogue of the types of peptides that could have existed on the early Earth. The amino acids Glu, Cys, and Gly have all been synthesized under model prebiotic conditions,^8–13^ and the prebiotic synthesis of oligopeptides has been reported by several mechanisms, including dry-wet cycling and α-aminonitrile ligation.^14–19^ Additionally, the presence of both α- and γ-peptide bonds within glutathione represents the types of heterogeneity expected for the nonribosomal synthesis of peptides.

We previously reported that exposure to UV light led to the formation of polynuclear iron–sulfur clusters coordinated to over 40 different peptides and small organic thiols, including glutathione.^7^ The data were interpreted to indicate that increased concentrations of hydrosulfide generated by the photolysis of Cys residues gave rise to [2Fe–2S] and then [4Fe–4S] clusters, but no mechanistic studies were carried-out. Difficulties in further deciphering the influencing factors stemmed from analyses that relied on Mössbauer spectroscopy of precipitated (with the addition of 2-propanol) and lyophilized aliquots of aqueous samples. The precipitation-lyophilization process likely changed molecular ratios and thus may have altered equilibria between different types of iron–sulfur clusters in solution. Additionally, the concentration of hydrosulfide generated by photolysis was not precisely known. Here, we more thoroughly interrogate the prebiotic synthesis of iron–sulfur clusters by a combination of UV-visible absorption, EPR, Mössbauer, and paramagnetic NMR spectroscopies in addition to cyclic and square wave voltammetries of soluble, aqueous samples with known concentrations of hydrosulfide. The data not only allowed us to more thoroughly characterize the types of iron–sulfur clusters and the influence of solution conditions but also to identify spectral fingerprints so that future efforts can more quickly ascertain the type of iron–sulfur cluster coordinated to oligopeptides. Although much excellent work on the abiotic synthesis of iron–sulfur clusters was reported by Holm and others,^20–24^ such studies were mostly in organic solvent with non-peptidyl ligands and largely did not address the role of ferric ions nor the inability to isolate reduced [2Fe–2S]^+^ cluster. Our results confirm the existence of multiple equilibria between different types of iron–sulfur clusters (Table S1†). Further, we were able to map out a synthetic path from mononuclear centres to [2Fe–2S] clusters and [4Fe–4S] clusters. This mechanism of cluster assembly is in agreement with the previously proposed assembly of a [4Fe–4S]^2+^ cluster in organic solvent from two [2Fe–2S]^+^ clusters.^25^ We also observed, at high ratios of HS^−^ to Fe^3+^, the formation of a higher nuclearity [6Fe–9S]^2−^ cluster coordinated to glutathione. Taken together, our work suggests that [4Fe–4S] peptides would have predominated on the prebiotic Earth in environments containing equimolar ratios of hydrosulfide and iron ions in the presence of small molecule thiolates. Conversely, environments rich in hydrosulfide would have led to the formation of a [6Fe–9S]^2−^ cluster that resembles the FeMo cofactor of nitrogenase.

## Experimental

### Materials and methods

All reagents were purchased from Sigma Aldrich and used without any further purification. Deionized MilliQ water was distilled under nitrogen to deoxygenate the solvent. The synthetic procedures to obtain the cluster were performed under controlled nitrogen atmosphere by using either a Schlenk line and Schlenk glassware or a nitrogen-purged glovebox. Samples were maintained under nitrogen (or argon) inert atmosphere and transferred to NMR tubes capped with rubber septa, EPR tubes capped with rubber septa, anaerobic sealed Hellma quartz cuvettes, electrochemical silicon-rubber sealed cells, or sealed glass vials for NMR, EPR, UV-visible, electrochemical, and Mössbauer investigation. Whenever possible, EPR and Mössbauer spectroscopies were conducted on the same sample, split in two and flash-frozen in liquid nitrogen. Measurements were mainly performed at 6 K and 20 K for EPR and at 4.2 K for Mössbauer spectroscopy. EPR spectra were recorded with a Bruker EMX spectrometer operating at X-band frequency with an ER-4116 dual mode cavity and an Oxford Instruments ESR-900 flow cryostat. Absolute quantification was based on comparison with a sample containing a known concentration of [4Fe–4S]^+^ cluster (see ESI† for more details). The fitting of absorbance at a fixed wavelength as a function of log[Zn^2+^] for the titration of iron–sulfur-containing solutions was performed with GraphPad Prism, version 6.00 (GraphPad Software, La Jolla, CA, USA) by means of a variable slope model equation (or 4-parameter dose–response curve, 4 PL, that is a sigmoidal curve symmetric around the midpoint).

### Synthesis of peptide-stabilized iron–sulfur clusters

Unless otherwise indicated, sodium sulfide (Na_2_S·9H_2_O, from 0 to 1 μmol, 0–1 mM) was added to an aqueous solution containing peptide (glutathione, 40 μmol, 40 mM) at pH 8.6 in a Schlenk round bottom flask under anaerobic conditions. Subsequently, ferric chloride (FeCl_3_·6H_2_O, 0.5 μmol, 500 μM) was added to obtain iron–sulfur peptides.

Higher concentrations of glutathione (150 μmol, 150 mM), sodium sulfide (from 0 to 3.75 μmol, 0 to 3.75 mM), and ferric chloride (1.88 μmol, 1.88 mM) were used to increase the signal-to-noise ratio for EPR, Mössbauer, and NMR spectroscopies. Additional improvement to the signal-to-noise ratio was achieved by reducing the ligand-to-metal molar ratio from 80 : 1 to 20 : 1 (150 mM glutathione, 0–15 mM Na_2_S, 7.5 mM FeCl_3_). Supplementary Table S2† reports a summary of the main spectroscopic and electrochemical properties of iron–sulfur glutathione complexes.

## Results and discussion

### The mononuclear species is rapidly reduced

**Fig. 1:**
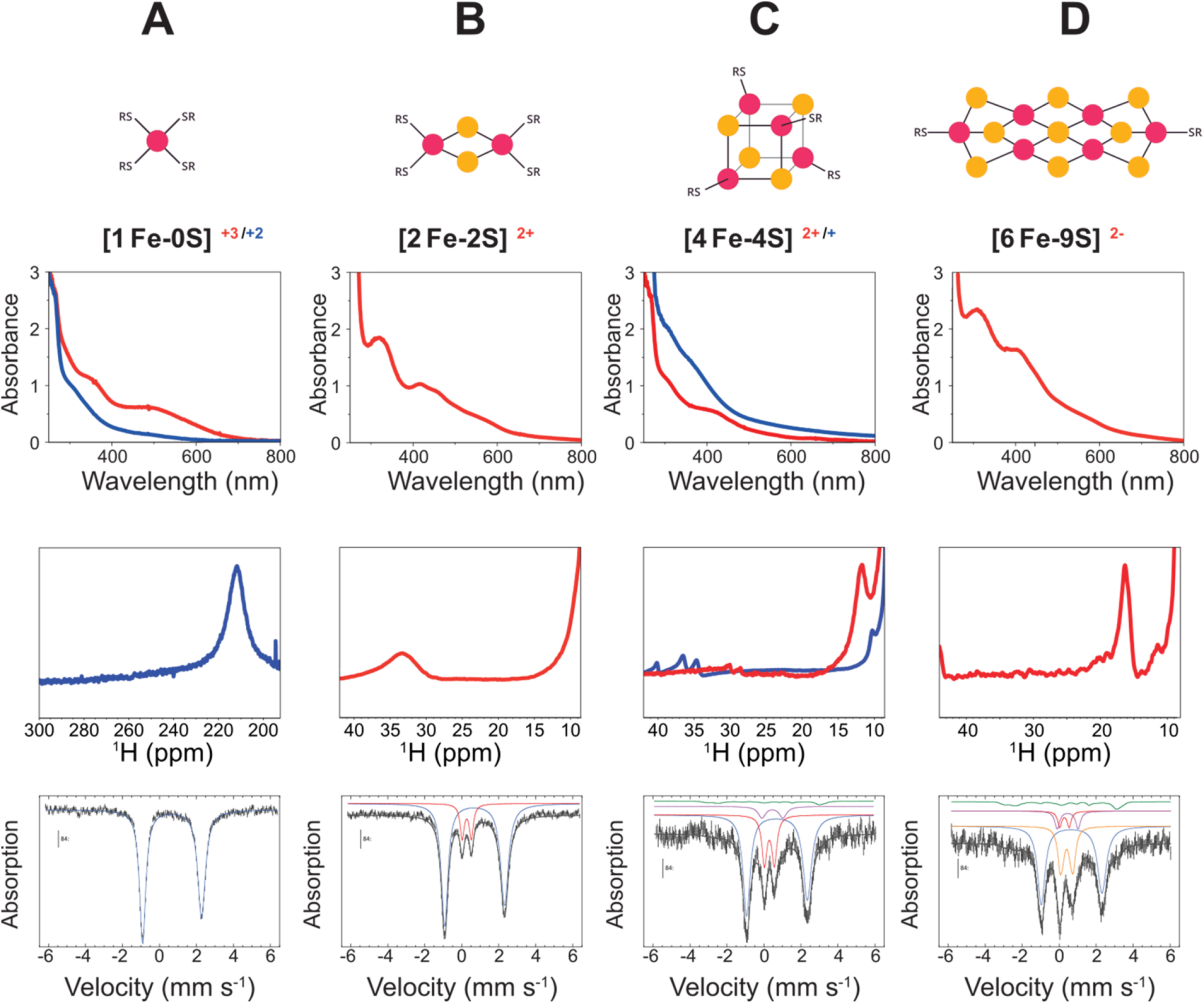
Spectroscopic characterization of solutions containing 0 : 1 (A), 0.4 : 1 (B), 1 : 1 (C), and 2 : 1 (D) HS^−^ : Fe^3+^ in the presence of glutathione showed the formation of [1Fe–0S] (A), [2Fe–2S] (B), [4Fe–4S] (C), and putative [6Fe–9S] (D) clusters. For each type of cluster, UV-visible absorption (top) and paramagnetic ^1^H NMR (middle) spectra are shown for the oxidized (red) and/or reduced (blue) states at pH 8.6 and 20 °C. The Mössbauer contributions (bottom) of the mono-, di-, tetra-, and hexanuclear species are provided in blue, red, mauve, and orange, respectively. The green solid line represents a reduced [2Fe–2S]^+^ cluster (see ESI, Fig. S4, bottom; Fig. S10, bottom; Fig. S24 and Fig. S33†).

To assess the effect of the ratio of hydrosulfide to iron ions on the type of iron–sulfur cluster formed, iron–sulfur clusters were synthesized with a constant concentration of FeCl_3_ and the tripeptide glutathione (EγCG) at pH 8.6 with varying concentrations of Na_2_S. Once dissolved in water at basic pH, Na_2_S gives rise to hydrosulfide (HS^−^).^26^ The ratio of hydrosulfide to Fe^3+^ varied from 0 : 1 to 2 : 1. When no hydrosulfide was present in solution, UV-visible absorption, paramagnetic ^1^H NMR, and Mössbauer spectroscopies showed the presence of a mononuclear Fe^2+^, rubredoxin-like species (Fig. 1A),^27^ as we previously observed.^6,7^

Although Fe^3+^ is insoluble at pH 8.6 (Fe(OH)_3_, *K*_sp_ = 10^−39^ M), Fe^3+^ was rapidly reduced to Fe^2+^ and kept in solution by coordination to peptides.^28,29^^1^H resonances at 3.3 and 3.0 ppm within the diamagnetic region of NMR spectra were indicative of oxidized glutathione, consistent with glutathione acting as the reductant of the iron (Fig. S1†). The standard reduction potential (*E*°) of glutathione at pH 8.6 is −0.34 V *vs.* SHE.^30^ Ferric ions have an *E*° of 0.77 V *vs.* SHE^31^ and are, therefore, promptly reduced to Fe^2+^. Control reactions in the absence of iron did not show the presence of oxidized glutathione (Fig. S1†). Upon reduction to Fe^2+^, all the resonances were broadened because of fast ligand exchange with high spin ions in solution, as previously observed (Fig. S1–S3†).^6,32^

Because of the rate with which Fe^3+^ was reduced to Fe^2+^ by glutathione, spectra of the oxidized state were only observed by UV-visible absorption spectroscopy. The spectra showed a band at 500 nm similar to what is typically observed for oxidized rubredoxin proteins^33,34^ and rubredoxin-like peptides.^27^ Rubredoxin contains a single iron ion coordinated by four cysteinyl thiolates. The band detected at 500 nm rapidly diminished without leaving a clear band in the visible region of the spectrum (Fig. 1A).^27^ Reduced rubredoxin similarly shows a featureless visible absorption spectrum.^33,34^ Paramagnetic ^1^H NMR spectra of samples with a featureless visible absorption spectrum showed a broad resonance at 211 ppm, as would be expected for Fe^2+^ coordinated to Cys (Fig. 1A).^27,35^ No EPR signal was detected, suggesting that neither free Fe^3+^ (*S* = 5/2) nor *S* = 1/2 paramagnetic iron–sulfur species were present. Integer spins, *e.g. S* = 2, are usually not detectable by low-field/low-frequency EPR setups and were not observed here. Mössbauer spectra were in agreement with the formation of [Fe(SR)_4_]^2−^ (Fig. 1A and S4†).^36^

To confirm the changes in reduction potential, the complexes were investigated further by electrochemistry. Square wave voltammetry (SWV) of solutions containing glutathione and iron ions showed a cathodic peak at −0.12 ± 0.01 V and an anodic peak at +0.04 ± 0.01 V. Both signals were well-shaped, although the anodic signal was broader and less intense. The *E*°′ value, calculated as the semi-sum of the peak potential values, was −0.04 ± 0.01 V (Fig. S5†), consistent with our prior measurements of mononuclear glutathione and *N*-acetyl l-cysteine methyl ester.^37^ This value was similar to that of rubredoxin, which typically falls between +0.06 and −0.08 V, depending on the specific construct.^38–40^ The large peak-to-peak separation (Δ*E*_p_ = 160 ± 8 mV) suggested a slow electron transfer process which could have arisen either from changes in geometry or changes in the coordination sphere between the reduced and oxidized forms. Since glutathione reacts rapidly with Fe^3+^ in solution to form Fe^2+^ and disulfide,^28,29^ and Fe^3+^-glutathione is unstable,^28,29^ the cathodic peak reasonably corresponded to the reduction of Fe^3+^ coordinated to oxidized glutathione^41^ formed on the electrode surface upon application of a positive potential. The following re-oxidation may have involved complexes of Fe^2+^ with oxidized, disulfide bridged glutathione and/or reduced glutathione (the latter being in large excess).^28,29^

### Ferric ions are trapped by the formation of [2Fe–2S]^2+^

[2Fe–2S]^2+^ glutathione was clearly observed in solutions containing 0.4 : 1 HS^−^ : Fe^3+^. The UV-visible absorption spectrum of the oxidized [2Fe–2S]^2+^ cluster showed the typical bands at 420 and 450 nm of [2Fe–2S]^2+^ ferredoxin^42^ (Fig. 1B) and of [2Fe–2S]^2+^ peptides, as previously reported.^6,27,32^ Paramagnetic ^1^H NMR spectra possessed a broad resonance at 33 ppm (Fig. 1B, centre), consistent with a [2Fe–2S]^2+^ cluster^27,35^ in addition to a resonance at 211 ppm from coexisting mononuclear species (Fig. S6†). The diamagnetic region of ^1^H and ^13^C NMR spectra showed less line broadening than seen for the mononuclear species (Fig. S7–S9†).

Mössbauer spectra of aqueous samples at 4.2 K confirmed the presence of oxidized [2Fe–2S]^2+^ cluster in addition to the mononuclear species at 0.4 : 1 HS^−^ : Fe^3+^ (Fig. 1B and S10†). The diamagnetic state of the [2Fe–2S]^2+^ cluster was indicated by the doublet detected at 4 K with 60 mT applied parallel to the γ-beam. EPR spectra contained signals indicative of two different types of paramagnetic iron–sulfur complexes. One signal was centred at 3515 G (*g* = 1.96) and was quite similar to a previously reported EPR signature of [2Fe–2S]^+^ cluster.^43,44^ The other was a more axial signal with a line centred at 3420 G (*g* = 2.02) representing an additional paramagnetic iron–sulfur species (Fig. S11†). Absolute spin quantification showed that these species were minor components of the solution (≈4% of the total iron for the [2Fe–2S]^+^) and were likely below the limit of detection by Mössbauer spectroscopy.

The major signals observed by square wave voltammetry were a reversible couple of peaks corresponding to the mononuclear centre. Additionally, a well-shaped shoulder at −0.20 ± 0.01 V was present and was attributed to the reduction of the [2Fe–2S]^2+^ cluster (Fig. S12†).^40^ The absence of the corresponding anodic signal indicated that the reduced form of the [2Fe–2S] cluster was unstable on the electrode surface and decomposed, as previously reported.^45^ Repeated steps of cyclic voltammetry (CV) confirmed degradation of the [2Fe–2S] cluster, as the cathodic peak of this species disappeared after the first cycle (Fig. S13†). The [4Fe–4S] cluster was not observed under these conditions by CV. Therefore, either the chemistry at the interface of the electrode hampered the reductive addition necessary for the formation of [4Fe–4S] clusters or the signals were not observable by voltammetry under these experimental conditions. Difficulty in observing polynuclear iron–sulfur clusters by voltammetry could be due either to absorption processes which decompose the clusters or to the overall high charge of [4Fe–4S] glutathione (6−/7−), which could prevent electron transfer at the surface of the electrode.^46,47^ Additionally, the high concentration of the charged peptide may have passivated the electrode making electron transfer slow.^46–49^ Similar mechanisms are also consistent with the large separation of anodic and cathodic peaks seen for the Fe^2+^/^3+^-glutathione adducts.

### [2Fe–2S]^2+^ converts to [4Fe–4S]^2+^

The UV-visible absorption, ^1^H NMR, and EPR spectra of [2Fe–2S]^2+^ glutathione slowly changed over time in solutions containing 0.4 : 1 HS^−^ : Fe^3+^ in a manner that suggested the formation of a [4Fe–4S]^2+^ cluster. Over a period of 2 h, the absorption band at 450 nm was lost, leaving a single band at ∼410 nm (Fig. 1C). Similarly, the resonance at 33 ppm decreased with a concomitant increase of a resonance at 11.8 ppm and a small doublet at 28 and 30 ppm, as would be expected for a [4Fe–4S]^2+^ peptide^27,35^ (Fig. 1C and S14†). The resonance corresponding to the mononuclear ferrous complex slightly decreased in intensity over time (Fig. S15†). Simultaneously, resonances assigned to oxidized glutathione were found to progressively increase in intensity in the diamagnetic region of the spectra (Fig. S16†), likely as a by-product of the transient formation of [2Fe–2S]^+^ in route to a cubane-like [4Fe–4S]^2+^. EPR spectra recorded for this sample 3 h and 24 h after mixing showed a general decrease in intensity and a change in the relative ratio of the two EPR-active species, with the proportion of [2Fe–2S]^+^ decreasing over time. If we assume that the relative ratio of paramagnetic species observed by EPR spectroscopy mirrors the relative ratio of diamagnetic cluster, then the additional axial spectrum may represent a [4Fe–4S]^+^ cluster (Fig. S17a and b†).

### [4Fe–4S]^2+^ is rapidly formed at equimolar sulfide-to-iron ratios

Equimolar concentrations of hydrosulfide and ferric ions showed the same series of events as solutions of 0.4 : 1 HS^−^ : Fe^3+^ but on a faster timescale. The d–d transition region of UV-visible absorption spectra initially showed bands at 420 nm and 450 nm similar to [2Fe–2S]^2+^ clusters and an additional broad band at 580 nm. Within 1 h, the bands at 420 nm, 450 nm, and 580 nm diminished, and a new peak at 410 nm emerged that was consistent with a [4Fe–4S]^2+^ cluster (Fig. S18†). Also in this case, diamagnetic ^1^H NMR spectra showed broadening for all of the ^1^H resonances of glutathione (Fig. S19†) but to a lesser extent with respect to what was observed for solutions containing [Fe(SR)_4_]^2−^ and the mixture [Fe(SR)_4_]^2−^/[2Fe–2S]^2+^ (Fig. S1 and S7†). Paramagnetic ^1^H NMR spectra confirmed the presence of a [4Fe–4S]^2+^ cluster (11.8, 28 and 30 ppm) in addition to small amounts of [2Fe–2S]^2+^ (33.3 ppm) and mononuclear species (211 ppm), plus an additional resonance at 15 ppm (Fig. S20 and S21†). After 12 h, no resonance corresponding to the [2Fe–2S]^2+^ cluster nor the mononuclear species was observed. Instead, the only strong resonances that remained were of the [4Fe–4S]^2+^ cluster and the additional resonance at 15 ppm, which was unchanged in intensity. The diamagnetic ^13^C NMR data were consistent with dynamics in between that of the high and low hydrosulfide conditions (Fig. S22 and S23†). Mössbauer spectra recorded at 4.2 K confirmed the presence of a mixture containing the mononuclear complex, [2Fe–2S]^2+^ and a probable [4Fe–4S]^2+^ cluster (Fig. 1C and S24†).^20^ Once again, EPR spectra indicated a mixture of [2Fe–2S]^+^ and [4Fe–4S]^+^ (Fig. S25†). Recordings at progressively higher temperature (10, 20, and 40 K) allowed for the detection of slight difference in relaxation properties between the two active EPR species, tentatively assigned to [4Fe–4S]^+^ and [2Fe–2S]^+^ (Fig. S26†). When a more dilute solution was interrogated, only a [2Fe–2S]^+^ cluster was observed immediately after mixing, likely reflecting slower kinetics of cluster formation (Fig. S25†).

Similar EPR findings were reported in previous studies of iron–sulfur glutathione systems at similar conditions, which were interpreted to indicate that [2Fe–2S]^+^ and [4Fe–4S]^2+^ clusters were the main species in solution.^43^ We also observed a comparable EPR spectrum for [2Fe–2S]^+^ and an additional signal attributed to [4Fe–4S]^+^. However, spin counting demonstrated that these two species were present at low concentrations and thus were minor contributors to the UV-vis and NMR spectra. We additionally collected Mössbauer spectra, which were dominated by a [4Fe–4S]^2+^ cluster rather than a [2Fe–2S]^+^ cluster (*ca.* 4% contribution, Fig. S24†). Any contribution of a [4Fe–4S]^+^ cluster was likely below the limit of detection. Square wave voltammetry of solutions containing 1 : 1 HS^−^ : Fe^3+^ revealed a new signal at more negative potential consistent with the [4Fe–4S]^+/2+^ redox couple (*E*°′ = −0.49 ± 0.02 V) (Fig. S27†).^50^ The intensity of the previously observed cathodic peak of the [2Fe–2S] cluster decreased until almost disappearing. The signals related to the mononuclear species remained almost unchanged.

In the absence of a rigid scaffold, it seems that the reduction of [2Fe–2S]^2+^ to [2Fe–2S]^+^ clusters rapidly leads to the formation of a [4Fe–4S]^2+^ cluster, as demonstrated by Holm and colleagues.^25^ Although [2Fe–2S] clusters appeared as intermediates rather than final products when ligated by peptides that possessed a single thiolate group, their role was important. The kinetic accessibility of [2Fe–2S] clusters^51^ and their slower rates of reduction by cysteinyl peptides in comparison to mononuclear centres ensured that ferric ions were preserved for sufficient time to allow for the formation of [4Fe–4S] clusters. That is, despite the likely photo-oxidation of Fe^2+^ to Fe^3+^ on the prebiotic Earth,^7^ the thiolate ligands needed for the formation of iron–sulfur clusters would have reduced Fe^3+^ to Fe^2+^ if polynuclear iron–sulfur clusters could not form rapidly.

### [6Fe–9S] forms at high hydrosulfide-to-iron ratios

Previous work only observed [2Fe–2S] or [4Fe–4S] clusters coordinated to oligopeptides.^27^ At 2 : 1 HS^−^ : Fe^3+^, we observed the appearance of a new higher nuclearity iron–sulfur cluster. This newly observed species showed a UV-visible absorption band at 410 nm and a paramagnetically shifted ^1^H resonance at 16 ppm (Fig. 1D), consistent with previous reports of a [6Fe–9S]^2−^ cluster.^52^ No mononuclear ferrous species was detected by NMR spectroscopy (Fig. S28†). The diamagnetic region of the ^1^H NMR spectrum showed less broadening than the spectra collected with lower HS^−^ to Fe^3+^ ratios (Fig. S29†). Furthermore, the ^1^H resonances of Glu and Gly possessed evident multiplicity, consistent with slower ligand exchange in comparison to those of the mononuclear and [2Fe–2S]^2+^ glutathione complexes and negligible interaction with the cluster core. Traces of oxidized cysteine were observed at 3.3 ppm. Similarly, ^13^C NMR spectra showed less paramagnetic-induced broadening (Fig. S30 and S31†) with integral ratios closer to those of the non-metallated peptide. Previous work assessed similar magnetic susceptibility per Fe at room temperature for [4Fe–4S]^2+^ and [6Fe–9S]^2−^, and a 50% larger value for [2Fe–2S]^2+^.^53^ Over time, no evidence of other species formed or of increased oxidized glutathione was observed indicating that this polynuclear iron–sulfur cluster possessed a reduction potential lower than that of glutathione. EPR spectroscopic spin counting was consistent with a negligible contribution of a paramagnetic [2Fe–2S]^+^ cluster with respect to the overall concentration of the metal ion in solution (11% of the total iron, Fig. S32†).

The Mössbauer spectrum showed an intense absorption at 0.72 mm s^−1^ that did not originate from either a [2Fe–2S]^2+^ or a [4Fe–4S]^2+^ cluster. The isomer shift and quadrupole splitting were more consistent with a [6Fe–9S]^2−^ cluster that presents a diamagnetic ground state (Fig. 1D and S33†).^54^ However, Mössbauer spectroscopy also showed the strong presence of the mononuclear [Fe(SR)_4_]^2−^ complex, which was not observed by NMR spectroscopy. Similar discrepancies with iron coordinated glutathione were previously observed and were interpreted to indicate the temperature sensitivity of redox equilibria in solution.^29^ Differences in peptide concentration may have also shifted equilibria. Therefore, the assignment of a [6Fe–9S]^2−^ cluster remains tentative until corroborated by further studies. It should be noted that the UV-vis spectrum at high ratios of HS^−^ to Fe^3+^ was previously interpreted to indicate a [4Fe–4S]^2+^ cluster.^7,43^ However, that spectrum is unlike previous reports of a [4Fe–4S]^2+^ cluster.^27,40,52^

To gain insight into the redox stability of the [6Fe–9S]^2−^ cluster, sodium dithionite, sodium ascorbate, hydrogen peroxide, and potassium ferricyanide were added. The addition of 1.5 equiv of sodium dithionite led to the loss of absorbance at 410 nm (Fig. S34†) and gave paramagnetic ^1^H NMR spectra that lacked resonances consistent with reduced or lower nuclearity iron–sulfur cluster (Fig. S35†). The data suggested that the higher nuclearity [6Fe–9S]^2−^ cluster rapidly degraded upon reduction. The addition of up to six equivalents of the weaker reductant sodium ascorbate failed to reduce or break the cluster, as no changes to the hyperfine resonances were observed (Fig. S36†). Neither the addition of one or two equivalents of the oxidants hydrogen peroxide or potassium ferricyanide gave rise to spectra indicative of the formation of additional iron–sulfur clusters.^35^ Paramagnetic ^1^H NMR spectra did not show alterations to the 16 ppm hyperfine resonance upon the addition of the oxidizing agents (Fig. S37 and S38†).

**Fig. 2:**
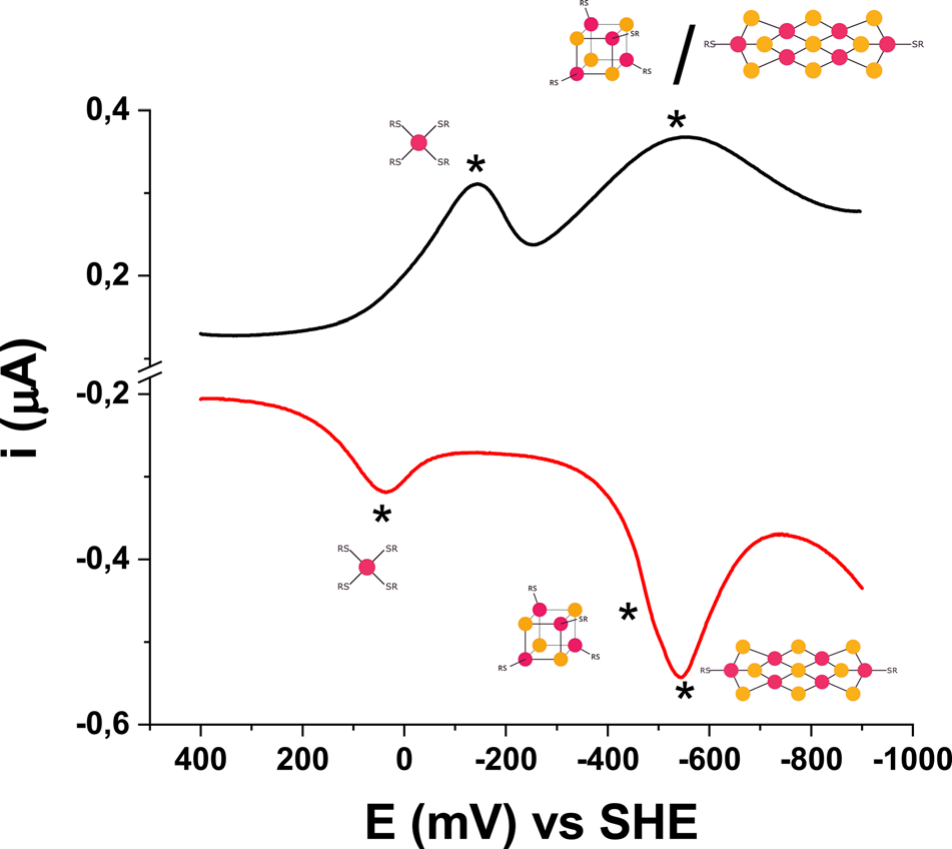
Square wave voltammetry of 2 : 1 HS^−^ : Fe^3+^ glutathione (40 mM glutathione, 4 mM Na_2_S, 2 mM FeCl_3_) in H_2_O, pH 8.6 and 20 °C. At this ratio, a well-defined anodic current peak is predominant at −0.55 ± 0.01 V, together with traces of [4Fe–4S] (shoulder at *ca.* −0.48 V). The peaks related to both the species are overlapped in the cathodic SW voltammogram (at −0.54V ± 0.01 V). Peaks related to mononuclear complex (−0.13 ± 0.01 V and 0.04 ± 0.01 V, cathodic and anodic peaks, respectively) are observed. The new species with *E*°′ ∼0.56 is tentatively assigned to a [6Fe–9S] cluster.

The reduction potential was then measured by square wave voltammetry at high ratios of hydrosulfide to iron. At 1.5 : 1 HS^−^ : Fe^3+^, an additional, reversible signal in the anodic scan at more negative potentials was observed (*E*_ap_ = −0.55 ± 0.01 V, Fig. S39†) that corresponded to the appearance of the higher nuclearity cluster, putatively ascribed to a [6Fe–9S] cluster.^52,54–57^ The corresponding cathodic peak was not resolved and overlapped that of the [4Fe–4S] cluster (*E*_cp_ = −0.54 ± 0.01 V). The reversible signal became prevalent at 2 : 1 HS^−^ : Fe^3+^ with *E*°′ = −0.56 ± 0.01 V (Fig. 2). Finally, when hydrosulfide was increased to over 2 : 1 HS^−^ : Fe^3+^, the intensity of both cathodic and anodic peaks decreased. The estimated reduction potential of the [6Fe–9S]^1−/2−^ cluster fell within the range of published values for the FeMo-cofactor of nitrogenase.^58^ For example, microcoulometry and EPR potentiometric titrations gave values ranging from −0.32 V to −0.58 V.^59–61^

### [2Fe–2S], [4Fe–4S], and [6Fe–9S] form in the presence of carbonate

Archean carbonate-rich lakes have been proposed as locations where the building blocks of life could have accumulated,^62,63^ and bicarbonate has been reported to increase the stability of iron–sulfur clusters.^64^ To determine whether the observed types of iron–sulfur clusters were compatible with the presence of carbonate, the electrochemical measurements were repeated in the presence of 100 mM bicarbonate at pH 8.6. At 0.4 : 1 HS^−^ : Fe^3+^, a composite signal in the cathodic scan consisted of a peak that corresponded to a [4Fe–4S] cluster (−0.5 V) with two poorly resolved shoulders, indicating a [2Fe–2S] cluster at *ca.* 0.21 V and an unidentified species at *ca.* −0.38 V (Fig. S40†). This new species also appeared in the anodic scan at −0.45 ± 0.01 V, in addition to a peak corresponding to the [4Fe–4S] cluster at *ca.* −0.49 V (Fig. S40†). An anodic signal corresponding to the [2Fe–2S] cluster was not observed, consistent with data collected in the absence of bicarbonate. However, an oxidation peak of a mononuclear species (+0.01 V) was clearly present indicating reductive conversion of the [2Fe–2S] cluster to a mononuclear centre. The recorded Mössbauer spectrum confirmed the presence of [1Fe–0S]^2+^, [2Fe–2S]^2+^, and [4Fe–4S]^2+^ centres, but *ca.* 40% of the iron (*vs.* the total iron content) was detected in a high-spin ferric state (Fig. S41†). That is, *ca.* 40% of the iron content did not correspond to [1Fe–0S]^2+^, [2Fe–2S]^2+^, or [4Fe–4S]^2+^ centres. The parameters for this high-spin ferric species strongly suggested a hexa-coordinate environment, which were consistent with EPR spectra of the same sample, where a signal at *ca. g* = 4.3 (Fe^3+^*S* = 5/2) was observed alongside a signal at *ca. g* = 2, attributed above to a [4Fe–4S]^+^ cluster (Fig. S42 and S11†). As the concentration of HS^−^ was further increased, a broad wave could be found at *E*_cp_ = −0.46 ± 0.01 V and *E*_ap_ = −0.45 ± 0.01 V, due to the overlap of the [4Fe–4S] signal and the signal of the additional species, which we have tentatively assigned to a [4Fe–4S] cluster with a different geometry or with a different set of ligands (Fig. S43†). In the presence of bicarbonate at 1 : 1 HS^−^ : Fe^3+^, the signal of this new, intermediate [4Fe–4S] cluster remained the most prevalent, but decreased at higher concentrations of HS^−^ (Fig. S44†). Under these conditions, another signal formed at −0.57 ± 0.01 V and at −0.56 ± 0.01 V in cathodic and anodic scans, respectively, which became prevalent at HS^−^ : Fe^3+^ > 2. This couple indicated the appearance of the higher nuclearity [6Fe–9S] cluster.^57,65–68^ Residual mononuclear species was also observed, indicating the degradation of the [2Fe–2S]^2+^ cluster.

Paramagnetic ^1^H NMR spectroscopy confirmed the presence of the [4Fe–4S]^2+^ cluster in addition to the [6Fe–9S]^2−^ cluster (Fig. S45†).^52^ However, no resonance attributable to a mononuclear complex was detected (Fig. S46†). Larger ratios of HS^−^ to Fe^3+^ led to a decrease in [4Fe–4S]^2+^ with a concomitant increase and broadening of the species with a resonance at 15.0 ppm. When HS^−^ was in excess, only a single broad peak at 16.0 ppm ([6Fe–9S]^2−^ cluster) was predominant in the paramagnetic upfield region. Only trace amounts of [4Fe–4S]^2+^ cluster were detected. No resonances corresponding to a mononuclear complex or a [2Fe–2S]^2+^ cluster were observed (Fig. S45†). UV-vis spectra at 1 : 1 HS^−^ : Fe^3+^ in 100 mM bicarbonate showed bands at 330, 420 (broad), and 578 nm, consistent with a mixture containing mostly [4Fe–4S]^2+^ cluster. The additional features of the UV-vis spectra,^52^ the resonance at 15 ppm in paramagnetic ^1^H NMR spectra, and the observed new cathodic (*ca.* −0.46 ± 0.01 V) and anodic (*ca.* −0.45 ± 0.01 V) electrochemical signals^52,55,69^ could be interpreted to indicate the presence of an intermediate iron–sulfur species similar to a [4Fe–4S]^2+^ cluster. Finally, at 2 : 1 HS^−^ : Fe^3+^, bands at 330, 416, and 578 nm were observed, consistent with previously reported spectra of a [6Fe–9S]^2−^ cluster (Fig. S47†).^52,57^ Taken together, all of the iron–sulfur cluster types that were observed in the absence of bicarbonate were also observed in the presence of bicarbonate.

### Potential synthetic pathways for iron–sulfur clusters

**Fig. 3:**
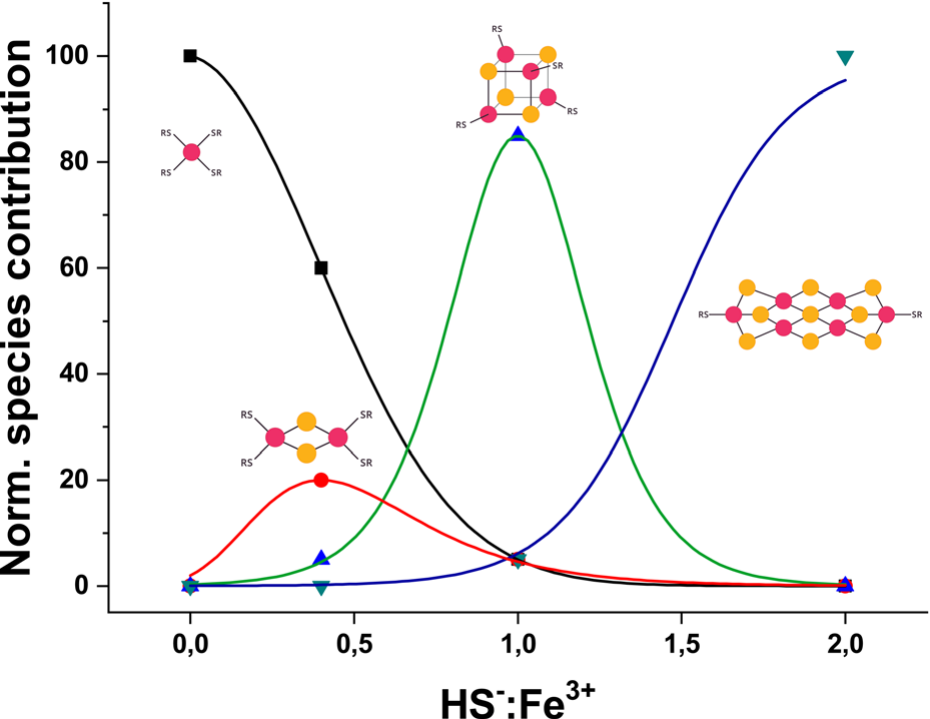
Normalized distribution of iron–sulfur glutathione as a function of HS^−^ : Fe^3+^. Ratios lower than one favour the formation of [2Fe–2S]^2+^ (red curve) and mononuclear complex (black curve). Ratios higher than one promote the formation of the putative [6Fe–9S]^2−^ (blue). Contribution of [4Fe–4S]^2+^ (green curve) is maximum at equimolar HS^−^ : Fe^3+^. Species contributions are obtained by UV-vis spectral decomposition and were fit to obtain each distribution curve, at 0 : 1, 0.4 : 1, 1 : 1 and 2 : 1 HS^−^ : Fe^3+^, respectively, as previously reported.^51^

There are clear boundary conditions that regulate what type of iron–sulfur cluster can form. If only ferrous and no ferric ions are present, then only the mononuclear complex can form, because fully reduced polynuclear iron–sulfur clusters, *i.e.* [2Fe–2S]^0^ and [4Fe–4S]^0^, are typically not stable (Fig. S48†).^6,27,70^ The opposite scenario in which only ferric but not ferrous ions are available can support the assembly of mononuclear centres and [2Fe–2S] clusters but not [4Fe–4S] clusters. This is because [2Fe–2S]^2+^ but not [4Fe–4S]^4+^ clusters are stable.^27^ Within this space between theoretically complete ferrous and complete ferric compositions, it is the concentration of hydrosulfide with respect to the iron ions that determines what type of iron–sulfur cluster can assemble (Fig. 3). Low HS^−^ to Fe^3+^ ratios favour the formation of mononuclear and [2Fe–2S]^2+^ glutathione.^43^ Higher ratios of HS^−^ to Fe^3+^ favour the formation of [4Fe–4S]^2+^ glutathione and then a [6Fe–9S]^2−^ cluster.

The mechanisms by which such iron–sulfur clusters form have been elucidated by Holm and colleagues.^25^ For example, in organic solvent and with non-peptidyl ligands, the primary route for the formation of [4Fe–4S] clusters proceeds through the condensation of two mixed-valence [2Fe–2S]^+^ clusters (Fig. S49†).^25,71^ This is logical since [2Fe–2S]^+^ and [4Fe–4S]^2+^ are the only two cluster types that share the same ratio of ferrous and ferric ions^27^ (Table S1†). We hypothezise that this same mechanism proceeds in aqueous solution by the reduction of [2Fe–2S]^2+^ units with excess glutathione. Cysteine-containing peptides (*E*°′ = −0.22 ± 0.25 V)^30,72,73^ are capable of reducing mononuclear centres (*E*°′ = −0.03 ± 0.06 V) but not [4Fe–4S]^2+^ clusters (*E*°′ = *ca.* −0.5 V).^39^ It is likely that glutathione slowly reduces the [2Fe–2S]^2+^ cluster (*E*_cp_ = *ca.* −0.2 V). The lower reduction potential of [4Fe–4S] clusters in addition to their decreased dynamics results in an accumulation of [4Fe–4S] clusters at the expense of lower nuclearity iron–sulfur species when the ratio of HS^−^ to Fe^3+^ is one or below.

The mechanism for the formation of the [6Fe–9S]^2−^ cluster is less clear. In biology, the FeMo cofactor is built with Nif proteins prior to insertion into nitrognease. After assembly of a [2Fe–2S] cluster on NifU,^74^ a [4Fe–4S] cluster and then a NifB coordinated [6Fe–9S] core,^75^ likely containing an interstitial carbide, is formed prior to the incorporation of an additional iron and molybdenum ion in route to the final FeMo cofactor. Our data suggest an analogous pathway in which [2Fe–2S] clusters mature into [4Fe–4S] and then [6Fe–9S] clusters. For example, an intermediate species between the appearance of the [4Fe–4S] and [6Fe–9S] clusters with features highly similar to a [4Fe–4S] cluster could be observed upon addition of increasing concentrations of hydrosulfide. Support for the existence of a modified [4Fe–4S] intermediate comes from SWV which shows the appearance of a [4Fe–4S] cluster (*E*_cp_ = −0.46 V, *E*_cp_ = −0.45 V, *E*°′ −0.45 ± 0.01 V) along with a [6Fe–9S]^1−/2−^ cluster (*E*_cp_ = −0.57 V, *E*_ap_ = −0.56 V). It may be that at HS^−^ : Fe^3+^ > 1 an iron ion dissociates from [4Fe–4S]^2+^ generating [3Fe–4S]^+^ clusters that combine with free HS^−^ to generate a [6Fe–9S]^2−^ cluster.^52,55,69,76^ Alternatively, [4Fe–4S]^2+^ units may become bridged by excess sulfide followed by loss of two ferrous ions, giving rise to an insoluble mixture of iron sulfide compounds as a by-product.^77^ Such a mechanism is consistent with the size of hydrosulfide, which is much smaller than glutathione and thus may be kinetically favoured for binding. Although the precise mechanism for the formation of the putative [6Fe–9S]^2−^ cluster was not clear, it was unlikely that the mononuclear species participated. For example, the addition of the chelator Na_4_EDTA to a solution containing 0.4 : 1 HS^−^ : Fe^3+^ removed the mononuclear species (Fig. S50 and S51†), and this solution supported the synthesis of the putative [6Fe–9S]^2−^ cluster upon the subsequent addition of hydrosulfide (Fig. S52†).

**Fig. 4:**
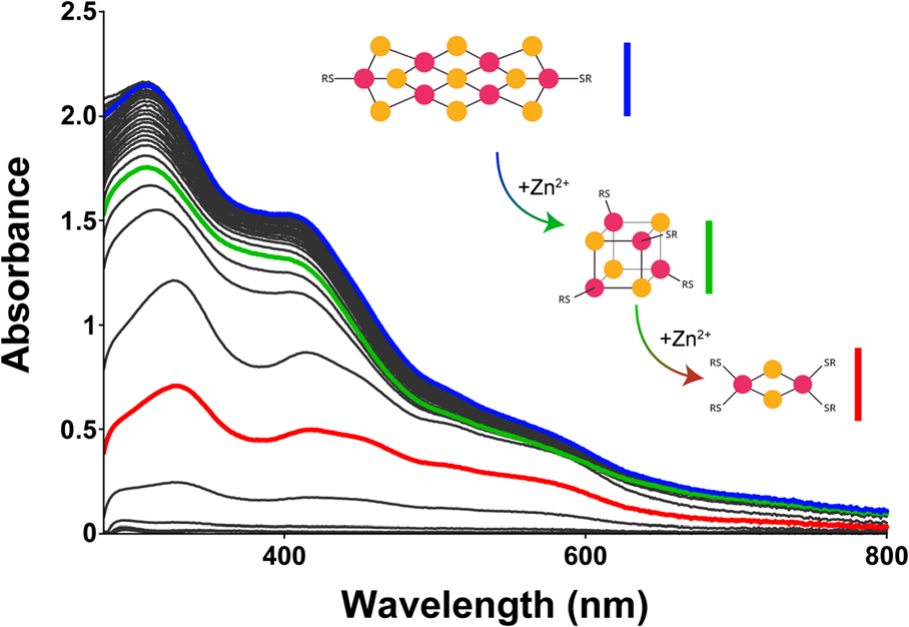
UV-visible absorption spectra of putative [6Fe–9S]^2−^ glutathione titrated with Zn^2+^ (pH 8.6 and 20 °C). As the concentration of Zn^2+^ increased, the intensity of the band corresponding to the putative [6Fe–9S]^2−^ cluster (410 nm, blue curve) decreased and shifted until the two absorption bands indicative of a [2Fe–2S]^2+^ cluster at 420 and 450 nm emerged (red curve). An intermediate species is observed (green curve).

To corroborate this putative mechanism, we sought to determine whether a [6Fe–9S]^2−^ would break down along the same path as observed for synthesis but in reverse order. [6Fe–9S] clusters were previously reported to break into [4Fe–4S] clusters.^78^ To do so, we titrated in Zn^2+^, which binds to the same sites as Fe^2+/3+^ but with increased affinity.^79^ Zn^2+^ could affect the type of complexes present by displacing bound Fe^2+/3+^ or by binding to free glutathione or hydrosulfide in solution. Upon the addition of Zn^2+^ to a solution containing a [6Fe–9S]^2−^ cluster, signatures of [4Fe–4S]^2+^ and [2Fe–2S]^2+^ (Fig. 4, green and red curves, respectively) were identified by UV-visible and ^1^H-NMR spectroscopies (Fig. S54–S56†). The concentration of Zn^2+^ needed to degrade half of the iron–sulfur cluster in the presence of 40 mM glutathione was 5.6 mM, 6.2 mM, and 11.3 mM for mixtures containing 0.4 : 1, 1 : 1, and 2 : 1 HS^−^ : Fe^3+^, respectively (Fig. S53–S55†). The data were consistent with [6Fe–9S] clusters breaking down to [4Fe–4S] and then [2Fe–2S] clusters, suggesting that [6Fe–9S] clusters are built from [4Fe–4S] units. It should be noted that even though Zn^2+^ binds to thiolate ligands with greater affinity than iron,^79^ the binding of iron ions was likely favoured on the prebiotic Earth because of the availability of each metal ion.^80^

Because of the differing ways that Zn^2+^ could have affected stability, and because of the different solution conditions used to generate each type of iron–sulfur cluster, it was difficult to assess the thermodynamic stability of each type of iron–sulfur cluster. In the presence of low ratios of hydrosulfide to iron ions, the [4Fe–4S] cluster was favored over other polynuclear iron–sulfur clusters. At higher ratios, the [6Fe–9S] cluster was favored over lower nuclearity iron–sulfur clusters. Both were consistent with the increased sulfide content of the iron–sulfur cluster with respect to ligating cysteines. That is, each iron centre was coordinated by two Cys and two S^2−^ for a [2Fe–2S] cluster, one Cys and three S^2−^ for a [4Fe–4S] cluster, and four of the iron centres of the [6Fe–9S] cluster were completely coordinated by S^2−^. Such analyses speak to kinetic accessibility but not necessarily to thermodynamic stability. Degradation likely resulted from the intrinsic dynamics associated with iron–sulfur clusters coordinated to peptides with a single Cys. On-, off-associations between Fe^3+^ and cysteine residues lead to the generation of highly reactive radicals, *e.g.* Cys-S˙, that degrade peptides. The linewidths of NMR spectra and electrochemistry showed that as the nuclearity of the cluster increased, the dynamics and the reduction potential of the complexes decreased, eventually below that of the non-metallated cysteinyl peptide. This suggests that higher nuclearity iron–sulfur clusters are more stable because of decreased opportunities to form radicals through interactions with iron ions. In fact, over the course of a day, the [4Fe–4S] cluster partially degraded, generating iron sulfide precipitates that were observable as sedimented black powder. Conversely, the [6Fe–9S] cluster did not degrade over the same period of time (Fig. S57†).

Holm and co-workers previously demonstrated that [Fe(SR)_4_]^2−^ progressively led to the formation of higher nuclearity iron–sulfur clusters in non-aqueous solvent with increasing concentrations of elemental sulfur.^52^ This was possible because elemental sulfur oxidized the iron ions. We show here that a similar pathway proceeds in water with a cysteinyl peptide and Fe^3+^ under conditions more easily extrapolatable to a prebiotic setting. Although cysteinyl peptides reduce Fe^3+^ to Fe^2+^, the rapidity with which polynuclear iron–sulfur clusters with lower reduction potential form protects much of the Fe^3+^ from reduction. This is consistent with higher concentrations of HS^−^ stabilizing Fe^3+^.^20^ Therefore, several factors impact the type of iron–sulfur cluster that can form, including the strength of the reducing environment, the concentration of hydrosulfide, the presence of competing metal ions, and the kinetics of assembly of the iron–sulfur cluster with the organic thiolates present in solution.

## Conclusions

Iron–sulfur clusters are thought to be ancient cofactors that were exploited by early, life-like chemical systems.^81–83^ The fact that few prebiotically plausible components, including iron ions, hydrosulfide, and thiolates, are sufficient to form the major classes of biological-like iron–sulfur centres in aqueous solution lends support to this hypothesis.^6,7,43,84^ While solution conditions and the type of thiolate-containing scaffold present impact the specific type of iron–sulfur cluster formed, iron–sulfur clusters are quite resilient to the presence of many chemicals and can form in seawater.^79^ Therefore, the boundary conditions are largely dictated by the availability of the chemical constituents of the iron–sulfur cluster. Since ferric ions are generally required to form iron–sulfur clusters,^20,27,85^ anoxic environments devoid of mechanisms to oxidize ferrous ions would be incapable of supporting the synthesis of iron–sulfur clusters. This poses a challenge to deep-sea hydrothermal vent environments that likely lacked Fe^3+^. Although Fe^2+^ can be photooxidized to Fe^3+^, such reactions require sunlight at the surface of the early Earth.^7^ Other proposed mechanisms for the generation of Fe^3+^ include volcanic emissions, lightening, and meteoritic impacts,^64^ all events compatible with surface and not deep-sea conditions. Calculations show the formation of Fe^3+^ in seawater containing dissolved nitrate and nitrite and no ammonium.^86^ Such conditions are not compatible with the consensus view of early anoxic seawater, which would have contained ammonium and negligible concentrations of nitrate and nitrite. In fact, there is direct geological evidence for ammonium in the Archean ocean.^87^ Furthermore, laboratory experiments meant to probe the prebiotic synthesis of iron–sulfur clusters at deep-sea hydrothermal vents were run at room temperature and surface pressures,^64^*i.e.* conditions far from that found at deep-sea hydrothermal vents.

Although aqueous surficial environments would have had access to ferric ions, access to hydrosulfide would have likely been more limited. Volcanism likely provided several sulfur species, including hydrogen sulfide (H_2_S), which would have existed in equilibrium with hydrosulfide (HS^−^) in shallow, surficial bodies of water.^63^ At alkaline conditions, which favors the retention of the hydrosulfide ion, hydrosulfide could have reached micromolar concentrations.^63^ One prebiotically plausible setting consistent with such conditions is an Archean carbonate-rich lake.^62^ Because of the complexation of carbonate with Ca^2+^, carbonate-rich lakes accumulate phosphate, a biologically important ingredient.^62^ Past reports^64,88^ and our data in this manuscript are generally consistent with the formation of iron–sulfur clusters in carbonate-rich lakes. Although high concentrations of Ca^2+^ would likely degrade iron–sulfur clusters,^88^ the composition of such surficial lakes are far from homogenous, providing gradients of pH and metal ions.^89^ An alternative surficial setting with plausibly high concentrations of hydrosulfide would be a shallow hydrothermal system, but their elevated temperatures would have disfavored the formation of iron–sulfur clusters coordinated to short peptides.^88^ Many of these stability issues would likely not be problematic if longer peptides were invoked. For example, dodeca-^84^ and tetradeca-^90^ iron–sulfur peptides can be remarkably stable. Therefore, the data thus far argue for specific niche environments particularly amenable to the stability of iron–sulfur clusters, *e.g.* subregions of alkaline, carbonate-rich lakes, or for the presence of longer cysteinyl-peptides that are better able to stabilize the iron–sulfur cluster in more commonly found environments.

Our observation that high concentrations of hydrosulfide with respect to iron leads to the formation of a putative [6Fe–9S]^2−^ broadens the types of iron–sulfur clusters that could have been environmentally available. However, confirmation of the precise nature of this iron–sulfur cluster would require isolation of this compound, which would be complicated by the intricate equilibria between different iron-containing species present in solution. More work is clearly needed to confirm the identity of this polynuclear iron–sulfur cluster. Excitingly, [6Fe–9S]^2−^ clusters geometrically resemble the FeMo cofactor of nitrogenase.^54,55^ When a similar [6Fe–9S]^2−^ cluster is inserted into nitrogenase in place of the natural FeMo cofactor, the enzyme retains catalytic activity.^91^ The metallocofactor substituted nitrogenase is capable of reducing acetylene to ethylene.^91^ Our work suggests that such nitrogenase-like iron–sulfur clusters would have emerged in prebiotic environments high in hydrosulfide, potentially providing an early catalyst that could have facilitated the emergence of protometabolism. Prebiotic environments may have possessed a broader suite of soluble iron–sulfur clusters than previously appreciated, perhaps generating the reduced nitrogen needed for the synthesis of amino acids and nucleic acids or facilitating the formation of carbon–carbon bonds.^92^

## Data availability

Data for this article, including UV-vis, Mossbauer, NMR, and EPR spectra, are available at Zenodo. See DOI: https://doi.org/10.5281/zenodo.14833746.

## Author contributions

Conceptualization: SS and SSM; funding acquisition: SG, GB, and SSM; investigation: SS, DR, MC, JR, AR; supervision: GG, MA, MB, SG, GB, and SSM; writing: all authors.

## Conflicts of interest

There are no conflicts to declare.

## Supplementary Material

SC-016-D5SC00524H-s001
