# Supplementary material for: Prebiotic synthesis of the major classes of iron–sulfur clusters
Source: Chem Sci. 2025 Feb 11;16(11):4614–24. doi: 10.1039/d5sc00524h (PMC11812447; doi:10.1039/d5sc00524h)
Supplement: SC-016-D5SC00524H-s001 [file SC-016-D5SC00524H-s001.pdf]

Supplementary Information (SI) for Chemical Science.
This journal is © The Royal Society of Chemistry 2025

Prebiotic synthesis of the major classes of iron-sulfur clusters

Simone Scintilla, Daniele Rossetto, Martin Clémancey, Julia Rendon, Antonio Ranieri, Graziano Guella, Michael Assfalg, Marco Borsari, Serge Gambarelli, Geneviève Blondin, and Sheref S. Mansy

DiCIBIO, University of Trento, Via Sommarive 9, 38123 Povo (TN), Italy.
Hudson River, Department of Biochemistry, Nieuwe Kanaal 7V, Wageningen, 6709 PA, NL.
Univ. Grenoble Alpes, CNRS, CEA, IRIG, Laboratoire de Chimie et Biologie des Métaux – UMR 5249, 17 rue des Martyrs, 38000 Grenoble, France.
CEA, Laboratoire de Résonance Magnétique, INAC/SCIB, UMR E3 CEA-UJF, 17, rue des Martyrs, 38054 Grenoble Cedex 9 (France).
University of Grenoble Alpes, CNRS, CEA, INAC-SyMMES, 38000 Grenoble, France.
Department of Life Sciences, University of Modena and Reggio Emilia, Via G. Campi, 103, 41125 Modena, Italy
Department of Physics, University of Trento, Via Sommarive 14, 38123 Povo (TN), Italy.
Department of Chemical and Geological Sciences, University of Modena and Reggio Emilia, Via G. Campi, 103, 41125 Modena, Italy.
Department of Biotechnology, University of Verona, Strada Le Grazie 15, 37134 Verona, Italy.
Department of Chemistry, University of Alberta, 11227 Saskatchewan Drive, Edmonton AB T6G 2G2, Canada.

The Supplementary Information contains:
Supplementary Methods
Supplementary Table 1-2
Supplementary Figures S1-S57

Supplementary Methods

NMR spectroscopy. NMR spectra were recorded at 25 °C using a 600 MHz Bruker Avance III spectrometer equipped with a triple resonance TCI cryogenic probe. Typical one-dimensional ^{1}H-NMR spectra were acquired with an excitation sculpting water suppression pulse sequence that utilized water selective 180° pulses. Acquisition parameters were 16 scans, 29761 Hz (50 ppm) spectral width, 4 s recycle delay, 0.550 s acquisition time, 32768 time domain data points. Exponential filtering of 1 Hz was applied prior to Fourier transformation. After Fourier transformation, the spectra were phase- and baseline-corrected manually. One-dimensional ^{1}H-NMR spectra optimized for paramagnetic samples were acquired with a water presaturation pulse sequence. A low power (50 Hz RF field) pulse was applied during the 0.08 s recycle delay. Acquisition parameters were 24 scans, 178571 Hz (298 ppm), 0.004816 s acquisition time, 1720 data points. The free induction decay was processed using 400 Hz exponential filtering prior to Fourier transformation (2048 points). For resonance assignment, two-dimensional ^{1}H homonuclear chemical shift correlation spectroscopy (COSY) and ^{1}H-^{13}C heteronuclear single quantum correlation (HSQC) experiments were used. Magnitude-mode multiple quantum filtered COSY spectra were acquired with presaturation of the water resonance during the recycle delay (2 s) using the following parameters: 16 scans, 2048 (F2, direct dimension) and 240 (F1, indirect dimension) data points, 7812 Hz (13 ppm) spectral width in both F1 and F2, 0.131 s acquisition time. The spectra were processed applying a $\pi/3$-shifted squared sine-bell function in both dimensions and zero-filling to a final spectrum size of 2048 × 1024 data points. HSQC experiments were acquired with a phase-sensitive standard pulse sequence incorporating a sensitivity-improvement scheme, shaped pulses for inversion, and gradients in the back-INEPT sequence. ^{13}C decoupling from ^{1}H was obtained by applying a GARP4 pulse train (decoupling pulse length 60 µs) during acquisition. Additional acquisition parameters were 16 scans, 1024 (F2, direct dimension) and 192 (F1, indirect dimension) data points, 9615 Hz (16 ppm, F2) and 30180 Hz (200 ppm, F1, centred at 70 ppm) spectral widths, 2 s recycle delay, 145 Hz ^{1}H-^{13}C coupling constant, and 500 µs pulse length of adiabatic inversion pulse. The spectra were processed applying a $\pi/2$-shifted sine-bell function in both dimensions and zero-filling to a final spectrum size of 1024 × 512 data points.

UV-Visible spectroscopy. UV-Visible absorption spectra of freshly prepared solutions were recorded with an Agilent 8453 UV-Visible diode array spectrophotometer with an integration time of 0.5 s and an interval of 1 nm.

EPR spectroscopy. Low temperature EPR spectra were collected with a Bruker EMX spectrometer operating at X-band frequency with an ER 4116DM dual mode cavity and an Oxford Instruments ESR-900 flow cryostat. Experimental parameters were as follows: temperature, 20, 40, 80 K; microwave frequency, 9.619 GHz; microwave power, 10 mW; modulation amplitude, 10 G; modulation frequency, 100 kHz, time constant, 40.96 ms; conversion time, 40.96 ms; number of scans, 20, number of points, 1024. The reference sample, the protein NifB from *Methanobacterium thermoautotrophicum* with two fully reduced $[4Fe\text{-}4S]^{+}$ clusters, was provided by Dr. Y. Nicolet and Dr. T.-Q. Nguyen (CEA, CNRS, IBS, Grenoble, France). The total S=1/2 spin concentration of this sample was previously determined to be 368 µM. The spin number was determined after baseline correction by double integration of the signal.

Mössbauer spectroscopy. Mössbauer spectra were recorded 4.2 and 80 K on a spectrometer equipped with a Janis SVT400 cryostat and on a strong-field Mössbauer spectrometer equipped with an Oxford Instruments Spectromag 4000 cryostat containing an 8 T split-pair superconducting magnet. The spectrometers were operated in a constant acceleration mode in transmission geometry. The isomer shifts were referenced against that of a metallic iron foil at room-temperature. Analysis of the data was performed with the software WMOSS Mössbauer Spectral Analysis Software (www. wmoss.org, 2012–2013, Web Research, Edina) and with a home-made software.[1–3] Samples were prepared by mixing the reagents in a nitrogen-purged glove-box and frozen in Mössbauer cups.

Electrochemical Measurements All the electrochemical measurements were performed at 20 °C. Cyclic and square wave voltammetry experiments were performed using a Potentiostat/Galvanostat mod. 273 A (EG&G PAR, Oak Ridge, USA). All measurements used a sealed cell for small volume samples (0.5 mL) under argon atmosphere, using a platinum ring and a saturated calomel electrode (SCE) as the counter and reference electrodes, respectively. A 1 mm diameter pyrolytic graphite (PGE) disk was used as the working electrode for cyclic and square wave voltammetry experiments. The electric contact between the SCE and the working solution was achieved with a Vycor (from PAR) set. All potentials were calibrated against the MV^{2+}/MV^{+} couple (MV = Methyl Viologen)[4] and were found to be reproducible within ±5 mV with 5% error on measured currents. Cyclic voltammetry was carried out at different scan rates (0.02 - 5 V s^{-1}). Square wave voltammetry conditions were with a frequency of 5 Hz and a pulse amplitude of 25 mV. All the formal reduction potentials (E°' values) are with respect to the standard hydrogen electrode (SHE).

The PGE electrode was first treated with anhydrous ethanol for 10 min, then polished with an alumina (BDH, particle size of ca. 0.015 µm) water slurry on cotton wool for 5 min. The electrode was then placed in an ultrasonic bath for 10 min. The formal reduction potentials E°' were calculated from the average of the anodic and cathodic peak potentials of cyclic voltammetry or square wave voltammetry (and were typically independent of scan rate in the range 0.02 - 5 V s^{-1}). For each species, the experiments were performed at least five times.

Synthesis of [Fe-S] clusters Mononuclear iron glutathione complexes were obtained by adding $FeCl_3$ (500 µM, 0.5 µmol) to glutathione (40 mM, 40 µmol, pH 8.6). Mixtures of mononuclear glutathione complexes and $[2Fe\text{-}2S]^{2+}$ were obtained by adding 0.4 equiv (185 nmol, 185 µM) of sodium sulfide with respect to ferric chloride (0.5 µmol, 500 µM) to a solution of glutathione (40 mM, 40 µmol, pH 8.6). Mixtures of mononuclear, $[2Fe\text{-}2S]^{2+}$ and $[4Fe\text{-}4S]^{2+}$ glutathione were obtained by adding one equiv (0.5 µmol, 500 µM) of sodium sulfide with respect to ferric chloride (0.5 µmol, 500 µM) to a solution of glutathione. $[4Fe\text{-}4S]^{2+}$ glutathione was collected as a single component upon incubating samples containing a mixture of the mononuclear complex and $[2Fe\text{-}2S]^{2+}$ glutathione at room temperature for over 180. The putative $[6Fe\text{-}9S]^{2-}$ cluster was obtained by adding two equiv (1.0 µmol, 1.0 mM) of sodium sulfide with respect to ferric chloride (0.5 µmol, 500 µM) to a solution of glutathione.

Supplementary Table 1. Number of ferric and ferrous ions for different types of iron-sulfur clusters.*

Cluster	Fe(III)	Fe(II)
$[1Fe\text{-}0S]^{3+}$	1	0
$[1Fe\text{-}0S]^{2+}$	0	1
$[2Fe\text{-}2S]^{2+}$	2	0
**$[2Fe\text{-}2S]^{+}$**	**1**	**1**
$[2Fe\text{-}2S]^{0}$	0	2
$[4Fe\text{-}4S]^{3+}$	3	1
**$[4Fe\text{-}4S]^{2+}$**	**2**	**2**
$[4Fe\text{-}4S]^{+}$	1	3
$[4Fe\text{-}4S]^{0}$	0	4
$[6Fe\text{-}9S]^{2-}$	4	2
$[6Fe\text{-}9S]^{-}$	5	1

*$[2Fe\text{-}2S]^{+}$ and $[4Fe\text{-}4S]^{2+}$ clusters contain the same ratio of reduced and oxidized iron ions are shown in bold. $[1Fe\text{-}0S]^{3+}$ and $[1Fe\text{-}0S]^{2+}$ signify oxidized and reduced, respectively, rubredoxin-like, mononuclear iron centres of $[Fe(SR)_4]^{-/2-}$.

Supplementary Table 2. Summary of the main spectroscopic and electrochemical properties of iron-sulfur glutathione.

	**$[1Fe\text{-}0S]^{3+}$**	**$[1Fe\text{-}0S]^{2+}$**	**$[2Fe\text{-}2S]^{2+}$**	**$[2Fe\text{-}2S]^{+}$**	**$[4Fe\text{-}4S]^{2+}$**	**$[4Fe\text{-}4S]^{+}$**	**$[6Fe\text{-}9S]^{-}$**	**$[6Fe\text{-}9S]^{2-}$**
**UV-visible absorption (nm)**	550	-	420<br>450	415	410	-	-	415
**^{1}H NMR paramagnetic (ppm)**	-	211	33.3	-	11.8<br>(28.6/30.11)<br>15.0	-	-	16.02
**E (vs SHE) (V)**	-0.12 (E_{red})	+0.04 (E_{ox})	-0.20 (E_{red})<br>-0.21 (E_{red})[#]	-	-0.50 (E_{red})<br>-0.50 (E_{red})[#]	-0.48 (E_{ox})<br>-0.49 (E_{ox})[#]	-0.54 (E_{red})<br>-0.57 (E_{red})[#]	-0.55 (E_{ox})<br>-0.56 (E_{ox})[#]
**EPR**	-[*]	-[*]	-[‡]	g=1.96	-[‡]	g=2.02	-*	[‡]
**Mössbauer** (δ / ΔE_Q in mm s^{-1})[%]	-/-	0.69 / 3.24	0.28 / 0.52	0.34 / 1.0<br>0.65 / 3.2[§]	0.46 / 1.13	-/-	- / -<br>- / -	0.41 / 0.72<br>0.40 / 0.43[&]

[#]In the presence of 100 mM sodium carbonate, pH 8.6.

[*]Not visible

[‡]Not EPR active

[%]For $[1Fe\text{-}0S]^{2+}$, $[2Fe\text{-}2S]^{2+}$ and $[4Fe\text{-}4S]^{2+}$, values are averages calculated from analyzed 4 K data reported in Figures S4, S10, S24 and S33.

[§] Parameters for the ferric and ferrous sites, respectively, in a 1:1 ratio, of the reduced two-iron ferredoxin from *Clostridium pasteurianum*[5]

[&] Parameters determined for $[Fe_6S_9(S^tBu)_2]^{4-}$.[6] The two doublets are in a 2:1 ratio.

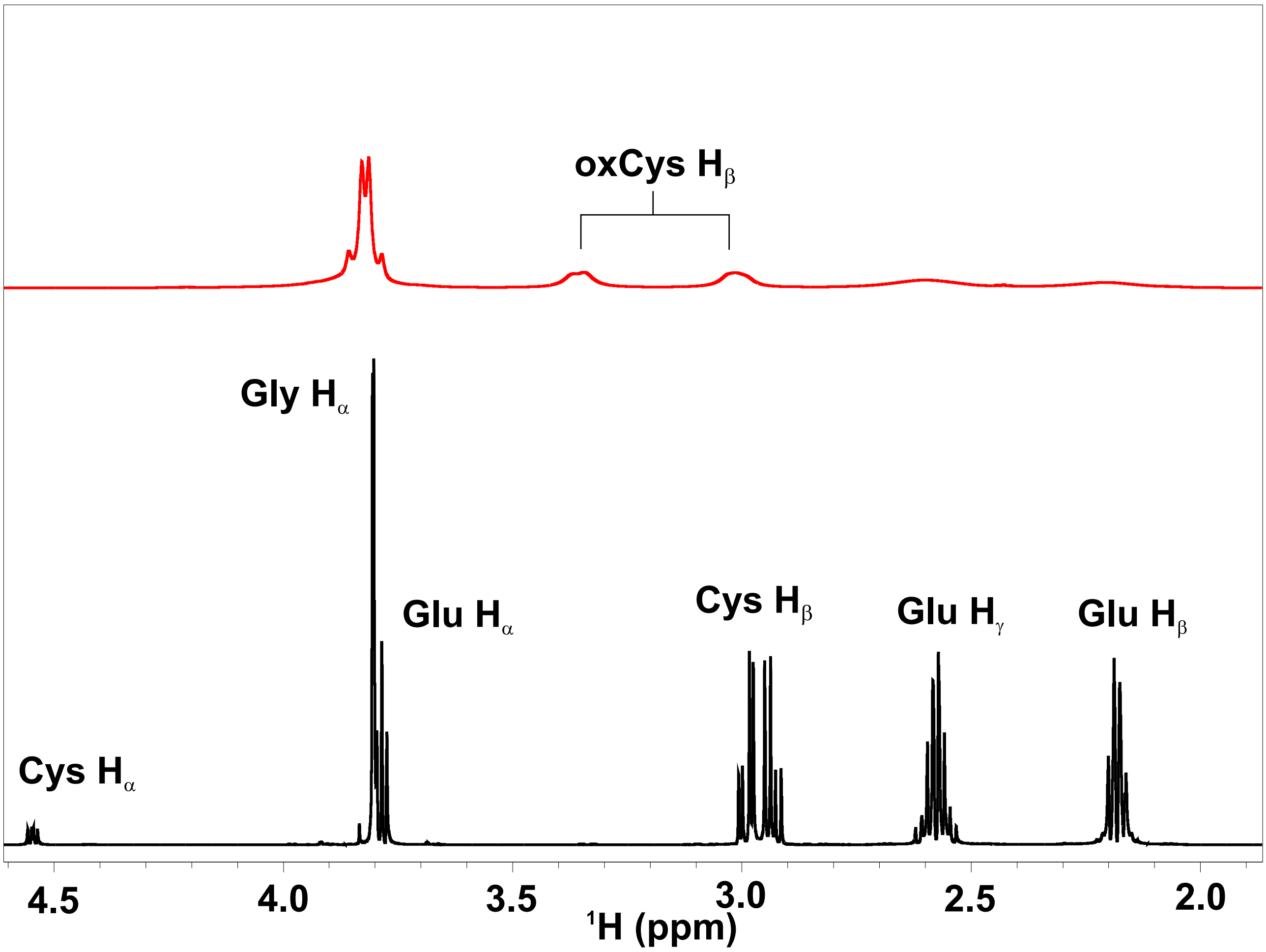

Supplementary Figure 1. ^{1}H NMR spectra of glutathione (black, 150 mM glutathione) and a solution obtained upon the addition of $FeCl_3$ to glutathione (red, 150 mM glutathione, 1.87 mM $FeCl_3$) in D_2O, pD 8.6. Upon the addition of ferric ions to the solution, severe broadening affected all the ^{1}H resonances of glutathione, suggesting a fast ligand exchange rate involving the whole bulk of the solution. In particular, Cys H_β (2.98 ppm) broadened to under the limit of detection so that only the signals corresponding to oxidized Cys H_β (3.01 and 3.34 ppm) upon glutathione oxidation by means of Fe^{3+} were detected. The ratio of integrals of Glu $H_{\beta,\gamma}$ (2.18 and 2.58 ppm) and Gly H_α (3.8 ppm) methylene groups dropped from 1 to 0.3 upon the addition of $FeCl_3$, suggesting a minor involvement of Gly residue in the interaction with the metal ion with respect to the Glu signals. The intensity of the signal corresponding to Cys H_α (4.55 ppm) was affected by the water suppression pulse (4.81 ppm). Therefore, the effect of the metal ion on the Cys H_α resonance was not interpretable. The resonances of Glu $H_{\alpha,B,\gamma}$ were broadened to a greater extent than that of Gly H_α, suggesting that Glu interacted more strongly with Fe^{2+} than Gly.

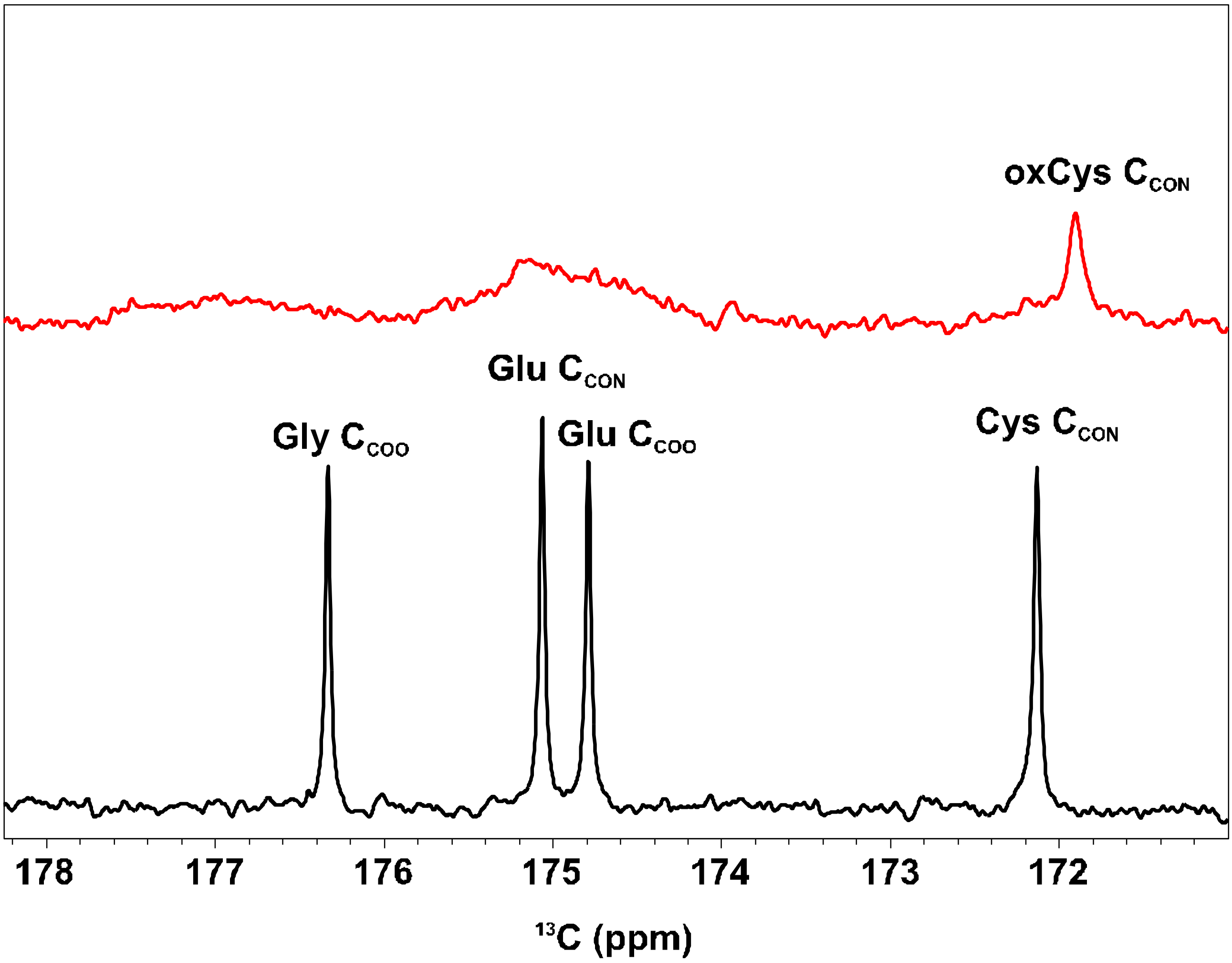

Supplementary Figure 2. ^{13}C NMR spectra of glutathione (black, 150 mM glutathione) and a solution obtained by the addition of $FeCl_3$ to glutathione (red, 150 mM glutathione and 1.87 mM $FeCl_3$) in D_2O, pD 8.6. The spectra refer to the carbonyl/carboxyl (CON/COO) region. Upon addition of ferric ions, a severe broadening of all of the resonances corresponding to glutathione $^{13}C_{CON}$ and $^{13}C_{COO}$ nuclei was observed due to the fast ligand exchange rate involving the whole bulk of the solution. A narrow resonance, corresponding to oxCys $^{13}C_{CON}$ could be detected at 171.85 ppm upon oxidation of glutathione by Fe^{3+}. However, no further ^{13}C resonances attributable to oxidized glutathione could be detected, likely due to interactions with paramagnetic Fe^{2+} in solution.[7]

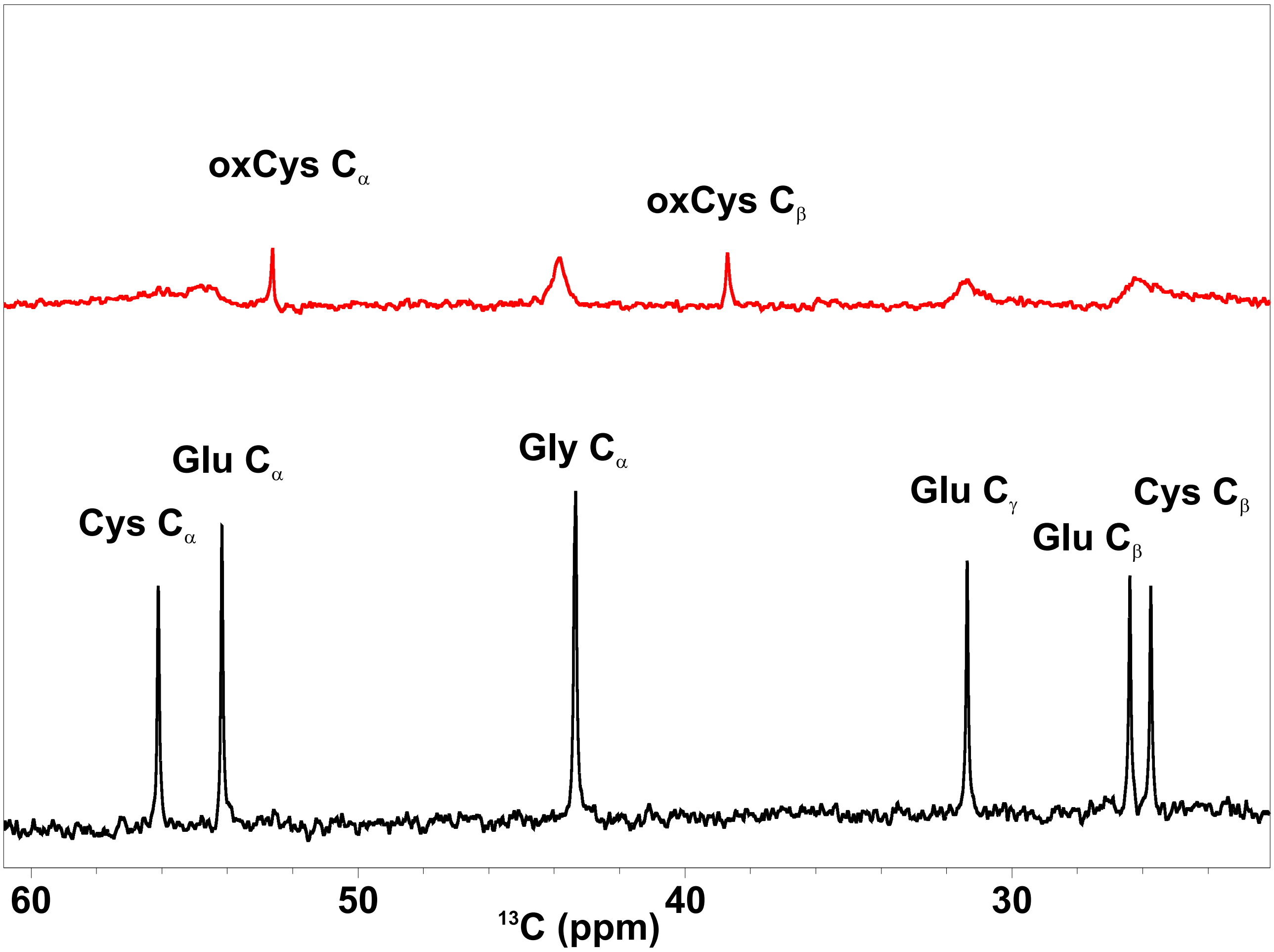

Supplementary Figure 3. The alkyl region of ^{13}C NMR spectra of glutathione (black, 150 mM glutathione) and a solution obtained by the addition of $FeCl_3$ to glutathione (red, 150 mM glutathione and 1.87 mM $FeCl_3$) in D_2O, pD 8.6. Upon the addition of ferric ions, severe broadening of all the resonances of the peptide is observed. In particular, the resonance corresponding to Cys C_α and C_β (56.12 and 25.75 ppm, respectively) broadened to under the limit of detection. The other broadened resonances included Glu $C_{\alpha,\beta,\gamma}$ (54.16, 26.38, and 31.36 ppm, respectively) and to a lesser extent Gly C_α (43.8 ppm). The presence of oxidized glutathione, arising from the reduction of Fe^{3+} to Fe^{2+}, was indicated by the sharp resonances of oxCys C_α and C_β at 52.59 and 38.75 ppm, respectively. However, no further ^{13}C resonances attributable to oxidized glutathione could be detected, likely due to interactions with paramagnetic Fe^{2+} in solution.

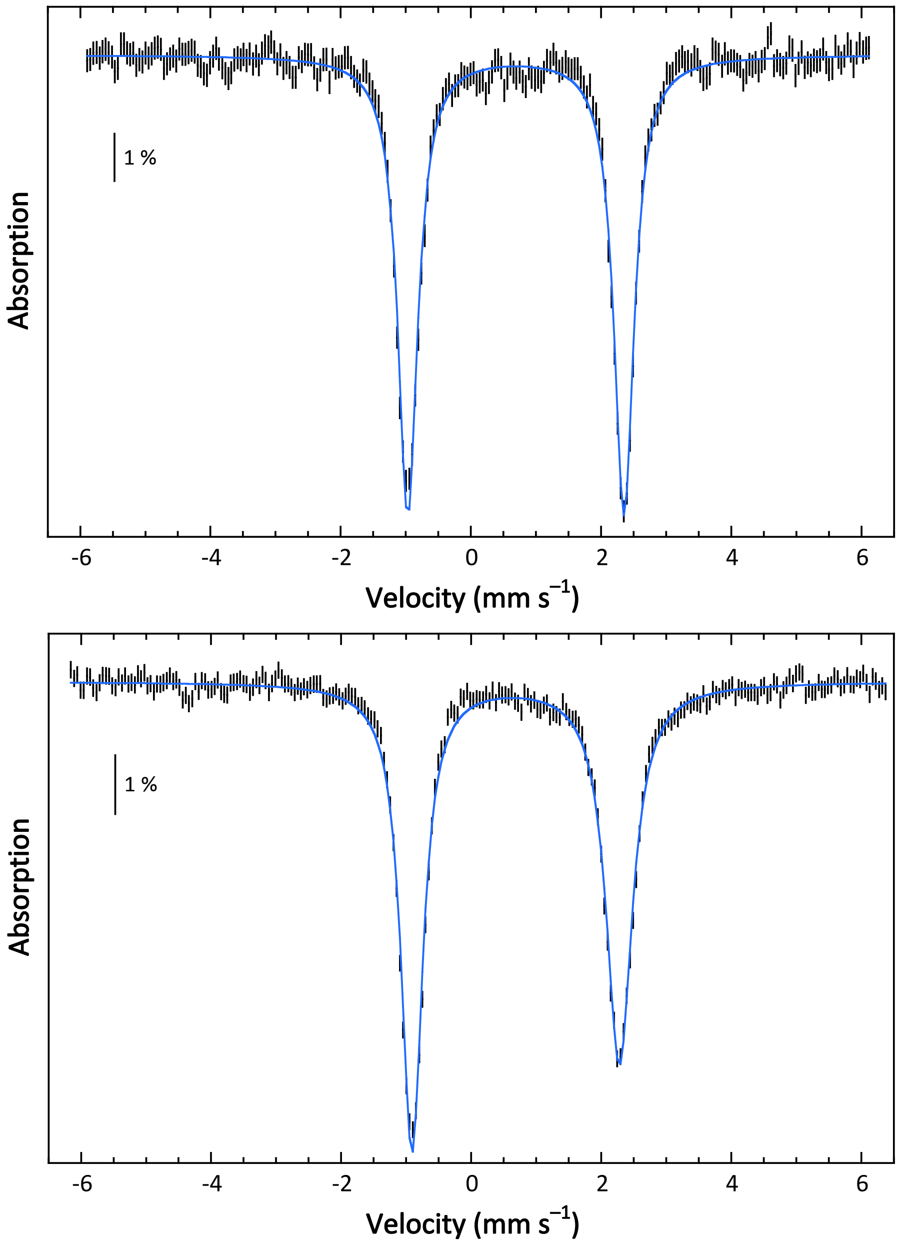

Supplementary Figure 4. Mössbauer spectra of the glutathione/$FeCl_3$ mixture (40 mM glutathione and 2 mM $FeCl_3$) in H_2O, pH 8.6 at 80 K and zero-field (top) and at 4.2 K with a 0.06 T external magnetic field applied parallel to the γ-rays (bottom). Both spectra present a single doublet indicating the presence of a single iron species. The nuclear parameters (δ = 0.69 mm s^{-1} and ΔE_Q = 3.22 mm s^{-1} at 80 K, δ = 0.68 mm s^{-1} and ΔE_Q = 3.19 mm s^{-1} at 4.2 K) are consistent with a high-spin Fe^{II} ion with a tetrahedral sulfur coordination. The doublet is less symmetric at 4.2 K, as is often seen for high-spin Fe^{II} species.

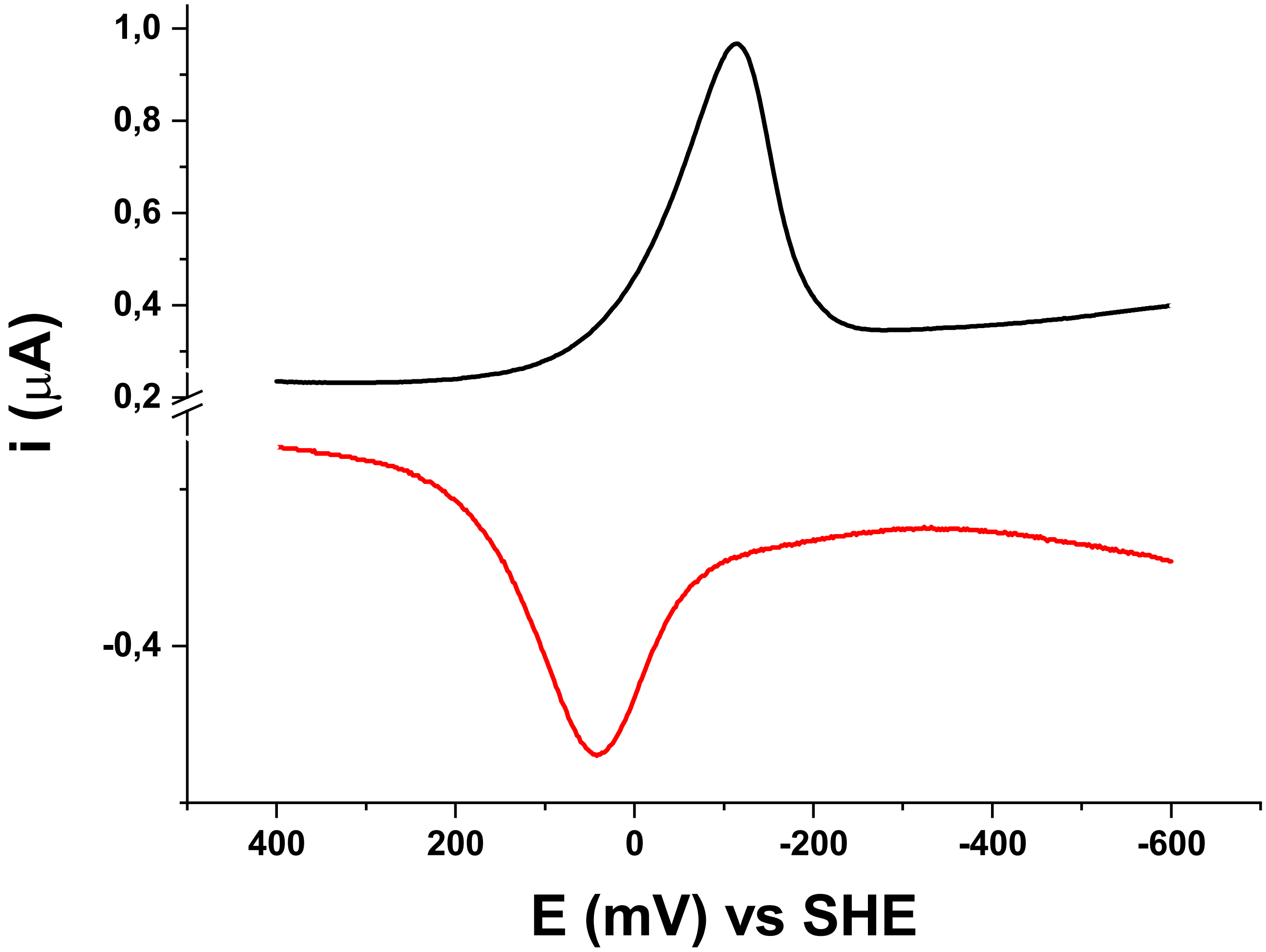

Supplementary Figure 5. Square wave voltammetry of 40 mM glutathione, 2 mM $FeCl_3$ in H_2O, pH 8.6. The mononuclear species gave rise to an intense peak at -0.12 ± 0.01 V in cathodic current (black) and to a peak at +0.04 ± 0.01 V in anodic current (red). The value of E°', -0.04 ± 0.01 V, was within the range of potentials reported for rubredoxin proteins (between -0.08 and + 0.06 V).[12] The large peak-to-peak separation (ΔE_p = 160 mV) suggested a slow electron transfer process, possibly due to changes in geometry between the reduced and oxidized forms.

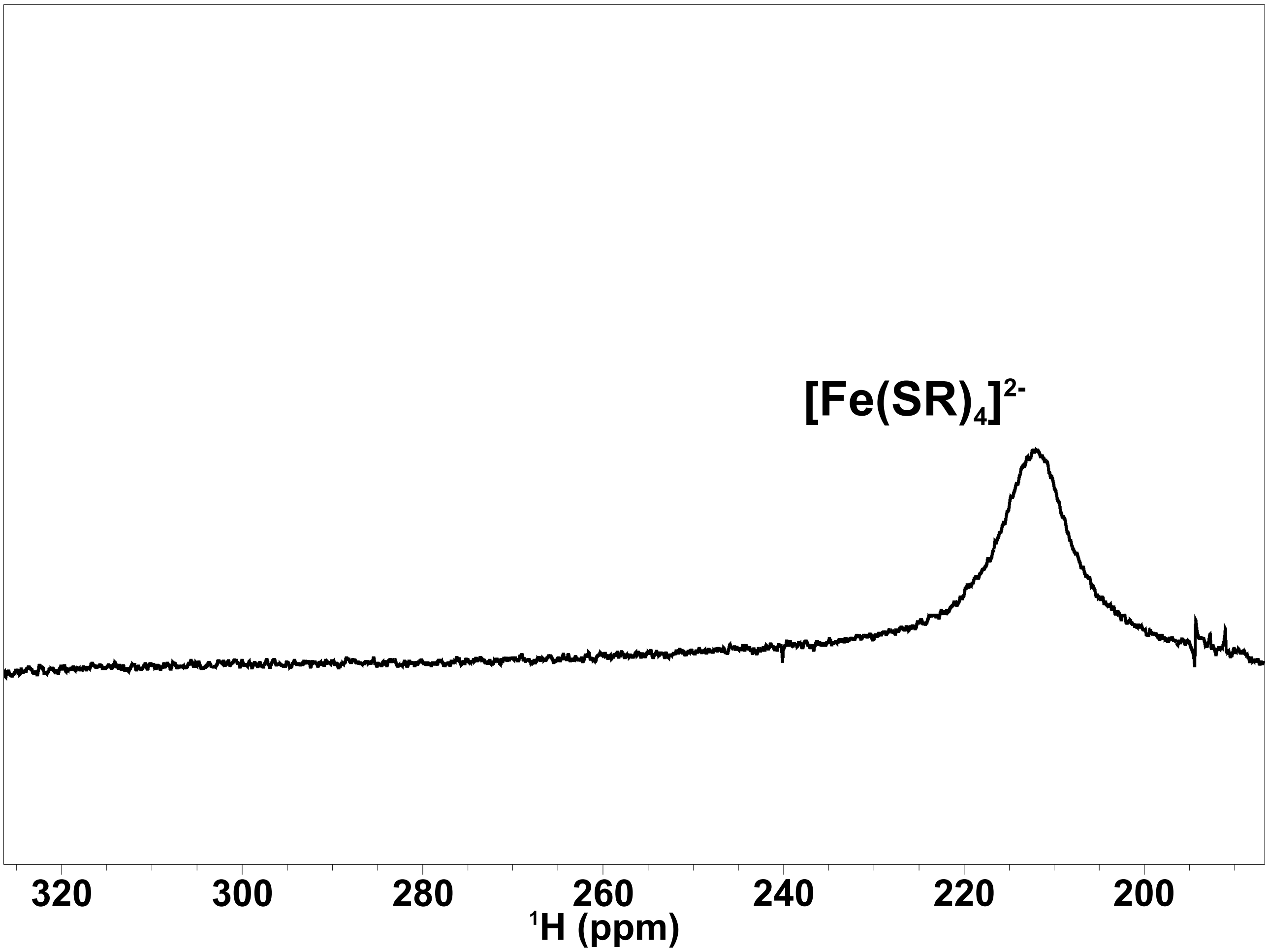

Supplementary Figure 6. Paramagnetic ^{1}H NMR spectrum of 0.4:1 HS^-:Fe^{3+} glutathione (150 mM glutathione, 0.75 mM Na_2S, 1.87 mM $FeCl_3$) in D_2O, pD 8.6. The resonance at 211 ppm is diagnostic of the presence of the reduced mononuclear species. This solution also contains a $[2Fe\text{-}2S]^{2+}$ cluster, as shown in **Fig. 1B** (centre, paramagnetic resonance at 33.3 ppm, red line). **Fig. S7** shows the diamagnetic region corresponding to the mixture. If all of the hydrosulfide was consumed to form a $[2Fe\text{-}2S]^{2+}$ cluster, then the solution consisted of 60% mononuclear species and 40% $[2Fe\text{-}2S]^{2+}$ cluster. The calculation was confirmed by a titration of the $[Fe(SR)_4]^{2-}$ complex with EDTA (**Fig. S50**).

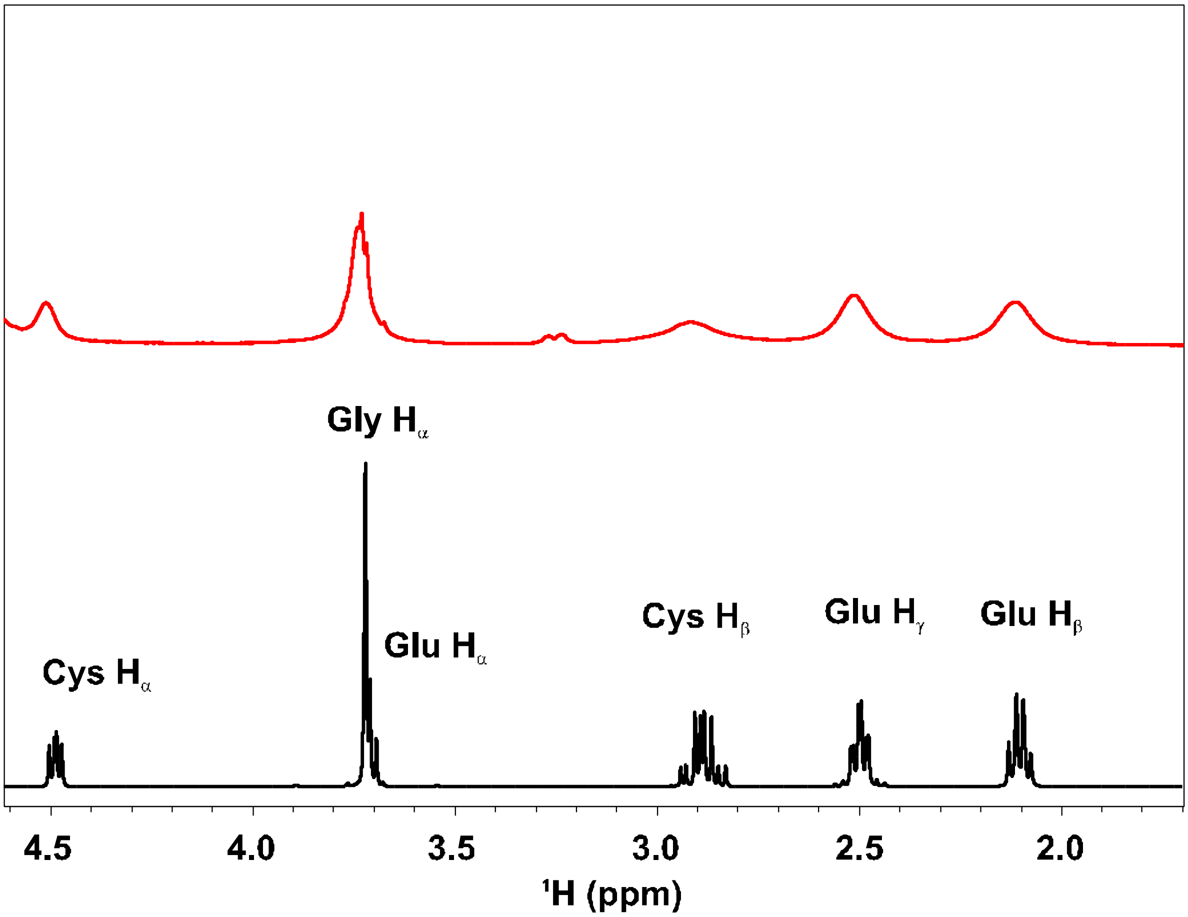

Supplementary Figure 7. ^{1}H NMR spectra of 150 mM glutathione (black) and 0.4:1 HS^{-}:Fe^{3+} glutathione (red) (150 mM glutathione, 0.75 mM Na_2S, 1.87 mM $FeCl_3$) in D_2O, pD 8.6. Upon the addition of hydrosulfide and ferric ions, all the ^{1}H resonances of the peptide were broadened. However, unlike spectra of the mononuclear species, a broad resonance of Cys H_{β} (2.89 ppm) was detected. Traces of oxidized Cys H_{β} were also observed (2.92 and 3.25 ppm) in solution. Although broadening was observed within the diamagnetic region, the line broadening was less than that observed for the mononuclear species (see **Fig. S1**). For example, the linewidth of the Glu H_{γ} resonance of $[2Fe\text{-}2S]^{2+}$ glutathione was 14 Hz, whereas the line-width of the same resonance was 95 Hz in the case of the mononuclear species. The ratio of integrals between the resonances corresponding to Glu $H_{\beta,\gamma}$ (2.06 and 2.45 ppm) and Gly H_{α} (3.68 ppm) was 1 for the non-metallated peptide and 0.8 for the solution that contained a mixture of $[2Fe\text{-}2S]^{2+}$ cluster and the mononuclear species. The data suggested that the Gly and Glu nuclei were more similarly affected by the presence of the $[2Fe\text{-}2S]^{2+}$ cluster than in the case of the mononuclear species (Glu $H_{\beta,\gamma}$/Gly H_{α} = 0.3). The intensity of the signal corresponding to Cys H_{α} (4.42 ppm) was influenced by the NMR water suppression pulse.

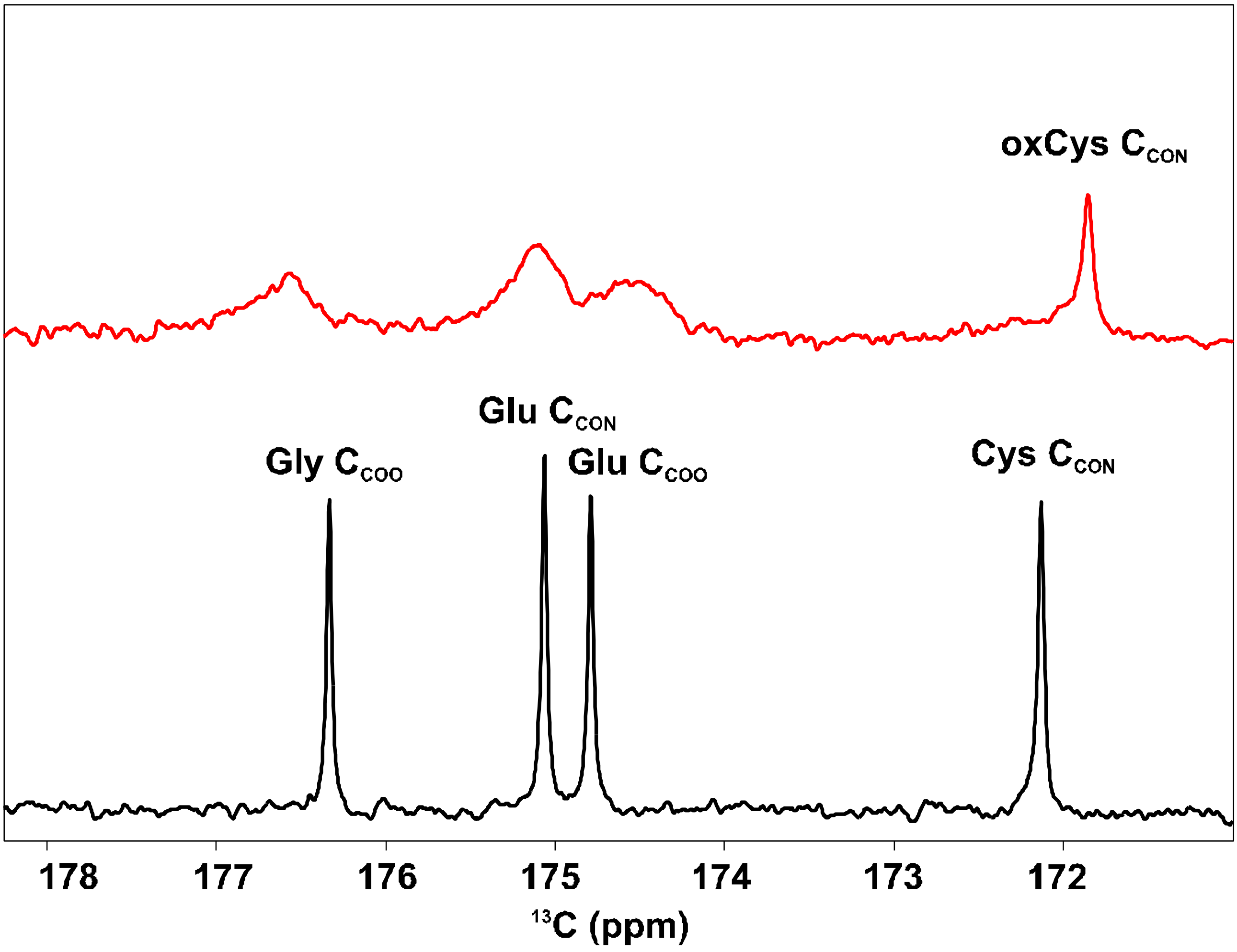

Supplementary Figure 8. ^{13}C NMR spectra of the carbonyl/carboxyl (CON/COO) region of 150 mM glutathione (black) and 0.4:1 HS^-:Fe^{3+} glutathione (red) (150 mM glutathione, 0.75 mM Na_2S, 1.87 mM $FeCl_3$) in D_2O, pD 8.6. Upon the addition of hydrosulfide and ferric ions, broadening was observed for all the $^{13}C_{CON}$ and $^{13}C_{COO}$ resonances. The spectrum showed less intense broadening for $[2Fe\text{-}2S]^{2+}$ glutathione in comparison to the sample containing the mononuclear species (**Fig. S2**). The most broadened signal corresponded to Cys $^{13}C_{CON}$ (172.03 ppm). A small, sharp signal corresponding to oxCys $^{13}C_{CON}$ (171.85 ppm) was also detected. However, no further ^{13}C resonances attributable to oxidized glutathione could be detected in solution.

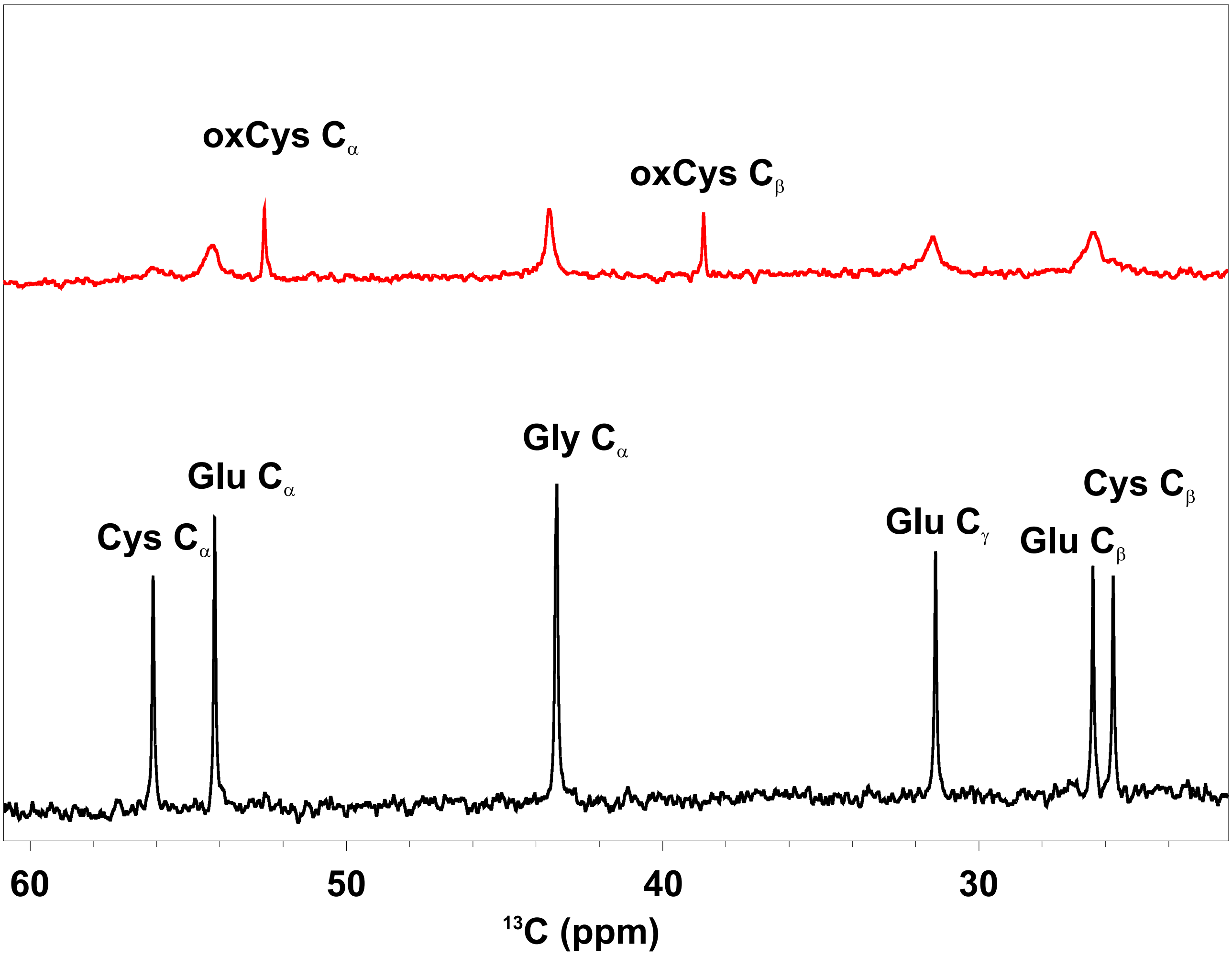

Supplementary Figure 9. ^{13}C NMR spectra of the alkyl region of 150 mM glutathione (black) and 0.4:1 HS^-:Fe^{3+} glutathione (red) (150 mM glutathione, 0.75 mM Na_2S, 1.87 mM $FeCl_3$) in D_2O, pD 8.6. Upon the addition of hydrosulfide and ferric ions, broadening was observed for all the ^{13}C resonances. In particular, the resonances corresponding to Cys C_α and C_β (56.12 and 25.75 ppm, respectively) were the most broadened. The broadening of Gly C_α (43.3 ppm) and Glu $C_{\alpha,\beta,\gamma}$ (54.16, 26.38 and 31.36 ppm, respectively) confirmed their involvement in the interaction with the iron-sulfur cluster. Similarly to what was observed in the case of the CON/COO spectral region (**Fig. S8**), the resonances of $[2Fe\text{-}2S]^{2+}$ glutathione underwent less intense broadening in comparison to that found for the mononuclear species (**Fig. S3**). Oxidized Cys C_α and C_β were found at 52.59 and 38.75 ppm, respectively. However, no further ^{13}C resonances attributable to oxidized glutathione could be detected in solution.

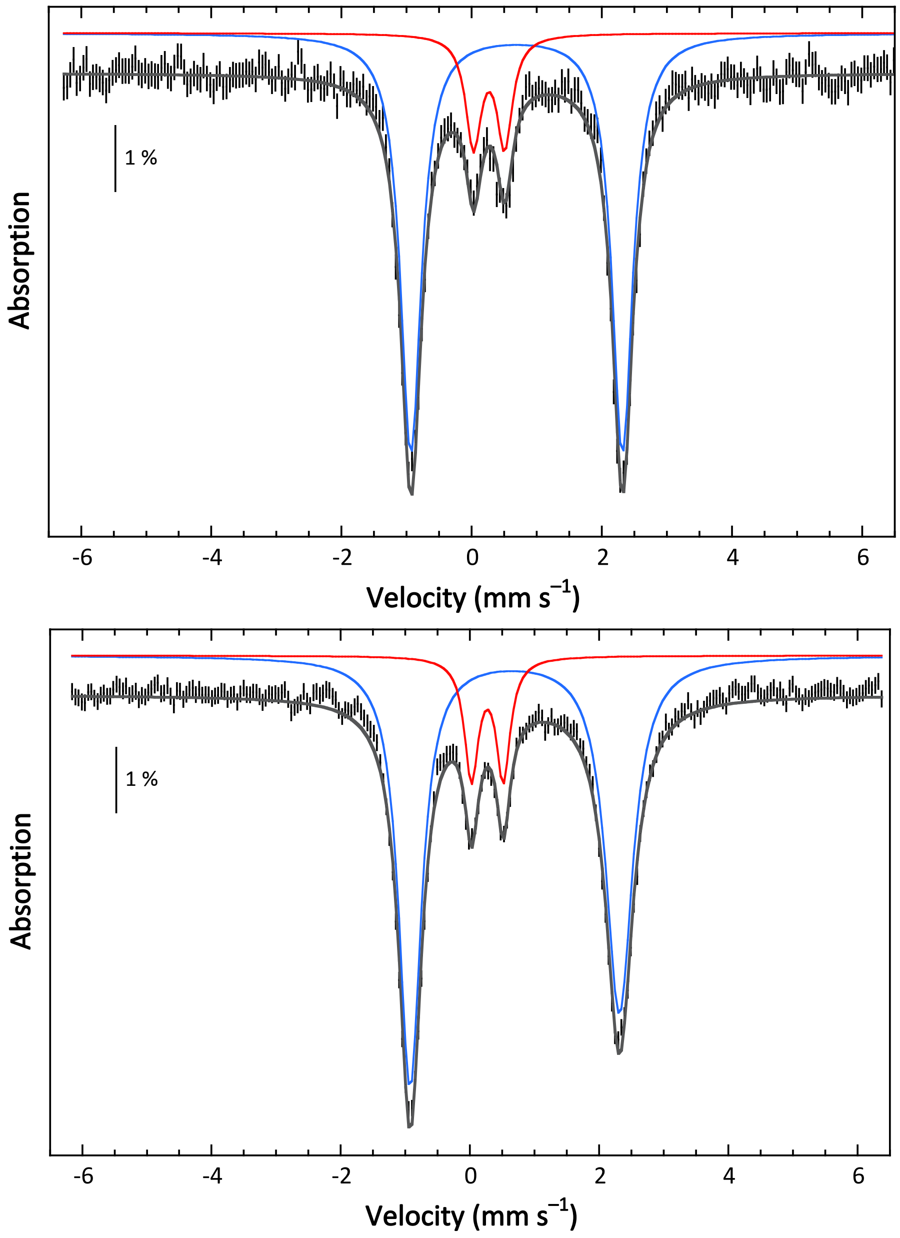

Supplementary Figure 10. Mössbauer spectra of the glutathione/HS^-/$FeCl_3$ mixture (160 mM glutathione, 0.74 mM Na_2S and 2 mM $FeCl_3$) in H_2O, pH 8.6 at 80 K and zero-field (top) and at 4.2 K with a 0.06 T external magnetic field applied parallel to the γ-rays (bottom). In addition to the previously characterized mononuclear tetrahedral high-spin Fe^{II} species (δ = 0.69 mm s^{-1} and ΔE_Q = 3.25 mm s^{-1} at 80 K, δ = 0.69 mm s^{-1} and ΔE_Q = 3.24 mm s^{-1} at 4.2 K, blue traces), a new species accounting for ≈15 % of the total iron content is evidenced (red traces). The nuclear parameters (δ = 0.27 mm s^{-1} and ΔE_Q = 0.48 mm s^{-1} at 80 K, δ = 0.27 mm s^{-1} and ΔE_Q = 0.49 mm s^{-1} at 4.2 K) and the diamagnetic character (doublet at 4.2 K) are consistent with the ferric sites of oxidized [2Fe-2S] cluster.

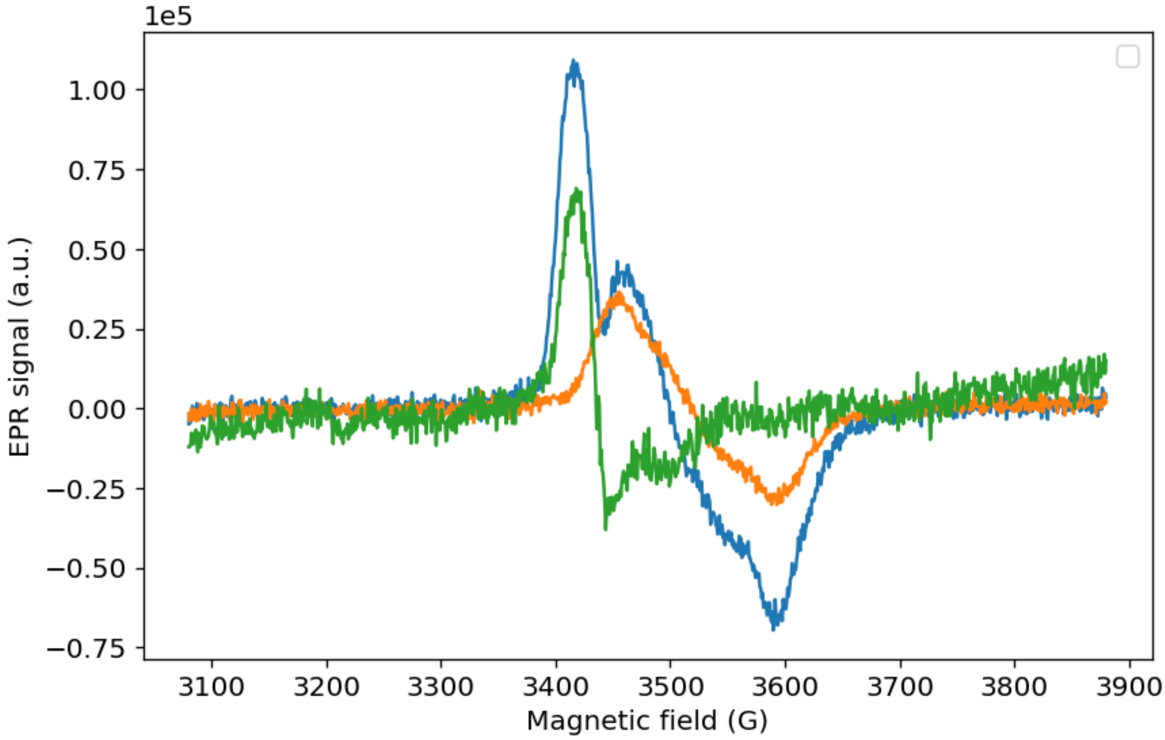

Supplementary Figure 11. EPR spectra of a solution containing 150 mM glutathione, 2.775 mM Na_2S, 7.5 mM $FeCl_3$ in H_2O, pH 8.6 at 15 minutes after mixing at 20 K (blue); of a solution containing 40 mM glutathione, 2 mM Na_2S and 2 mM Fe^{3+}, pH 8.6 15 minutes after mixing at 20 K (orange); and of a solution containing 15 mM glutathione, 0.74 mM Na_2S, 2 mM $FeCl_3$ and 100 mM carbonate in H_2O, pH 8.6 15 minutes after mixing at 10K (green, zoom 2x). In first approximation, the blue spectrum contains contributions from $[2Fe\text{-}2S]^+$ cluster (3515 G, g=1.96) and $[4Fe\text{-}4S]^+$ cluster (3420 G, g=2.02), while orange and green correspond to pure $[2Fe\text{-}2S]^+$ and $[4Fe\text{-}4S]^+$ clusters, respectively. Spin counting with comparison with a sample containing a known concentration of a $[4Fe\text{-}4S]^+$ shows that the pure $[2Fe\text{-}2S]^+$ (orange trace) corresponds to 4 % of total iron. The counting for $[4Fe\text{-}4S]^+$ (green spectrum) is 0.5% of the total iron (with a rather large uncertainty because of the low signal/noise ratio).

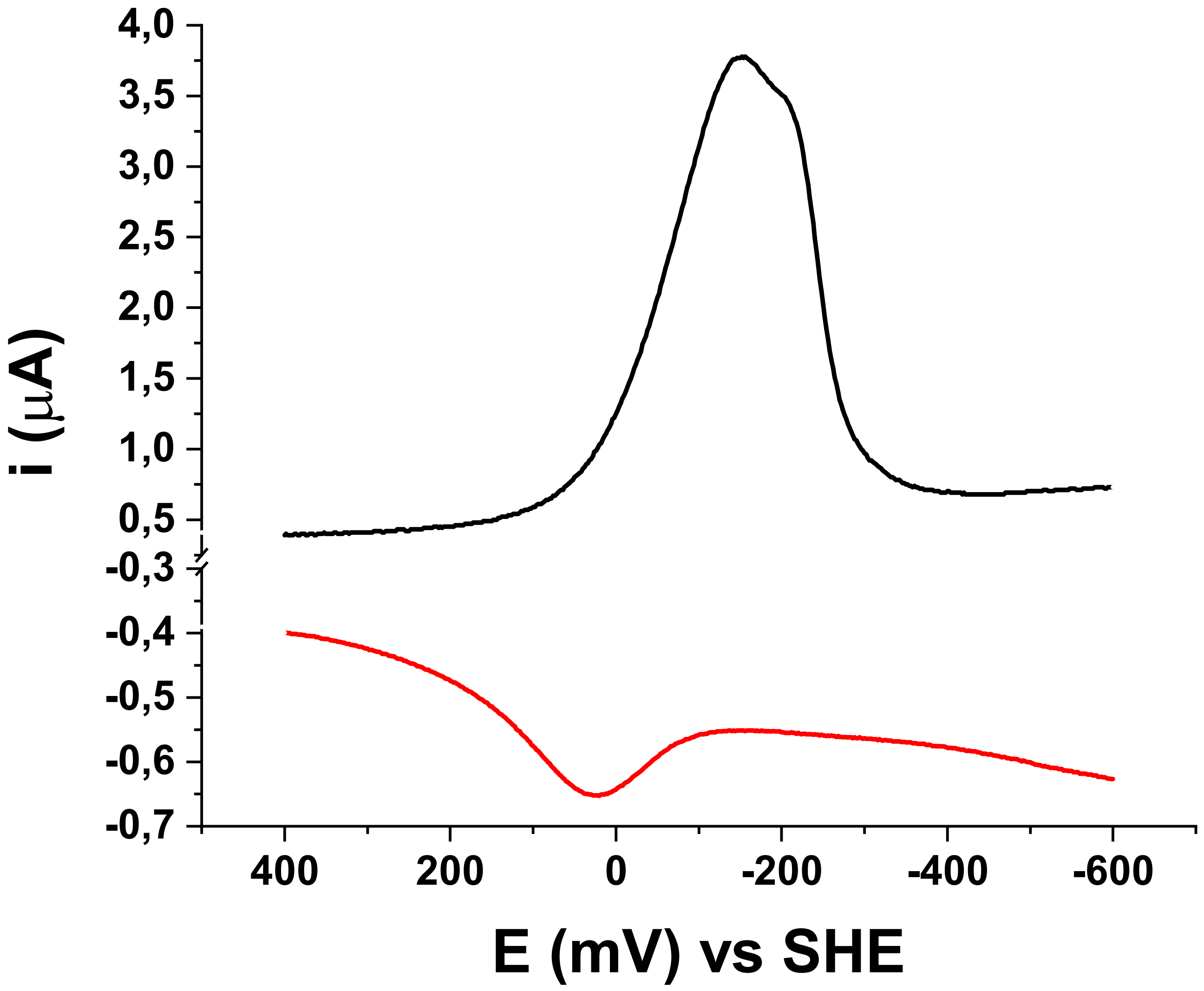

Supplementary Figure 12. Square wave voltammetry of 0.4:1 HS^-:Fe^{3+} glutathione (40 mM glutathione, 0.8 mM Na_2S, 2 mM $FeCl_3$) in H_2O, pH 8.6. Cathodic square wave voltammetry (black curve) showed one peak at -0.13 ± 0.01 V, which was attributed to the mononuclear species that also had a shoulder at -0.20 ± 0.01 V. The shoulder was assigned to a $[2Fe\text{-}2S]^{2+}$ cluster. Anodic square wave voltammetry (red curve) showed only a single peak without an evident shoulder at +0.04 ± 0.01 V, which was attributed to the mononuclear species. Under these conditions, the mononuclear species showed a formal reduction potential E°' = -0.04 ± 0.01 V. The shift (8 mV) experienced by the cathodic peak of the mononuclear species upon addition of HS^- is attributed to the overlap with the [2Fe-2S] reduction peak (see **Fig. S5**). The presence of only a single anodic peak was consistent with the reductive degradation of [2Fe-2S] glutathione into mononuclear species, as confirmed by repeated steps of cyclic voltammetry reported in **Fig. S13**.

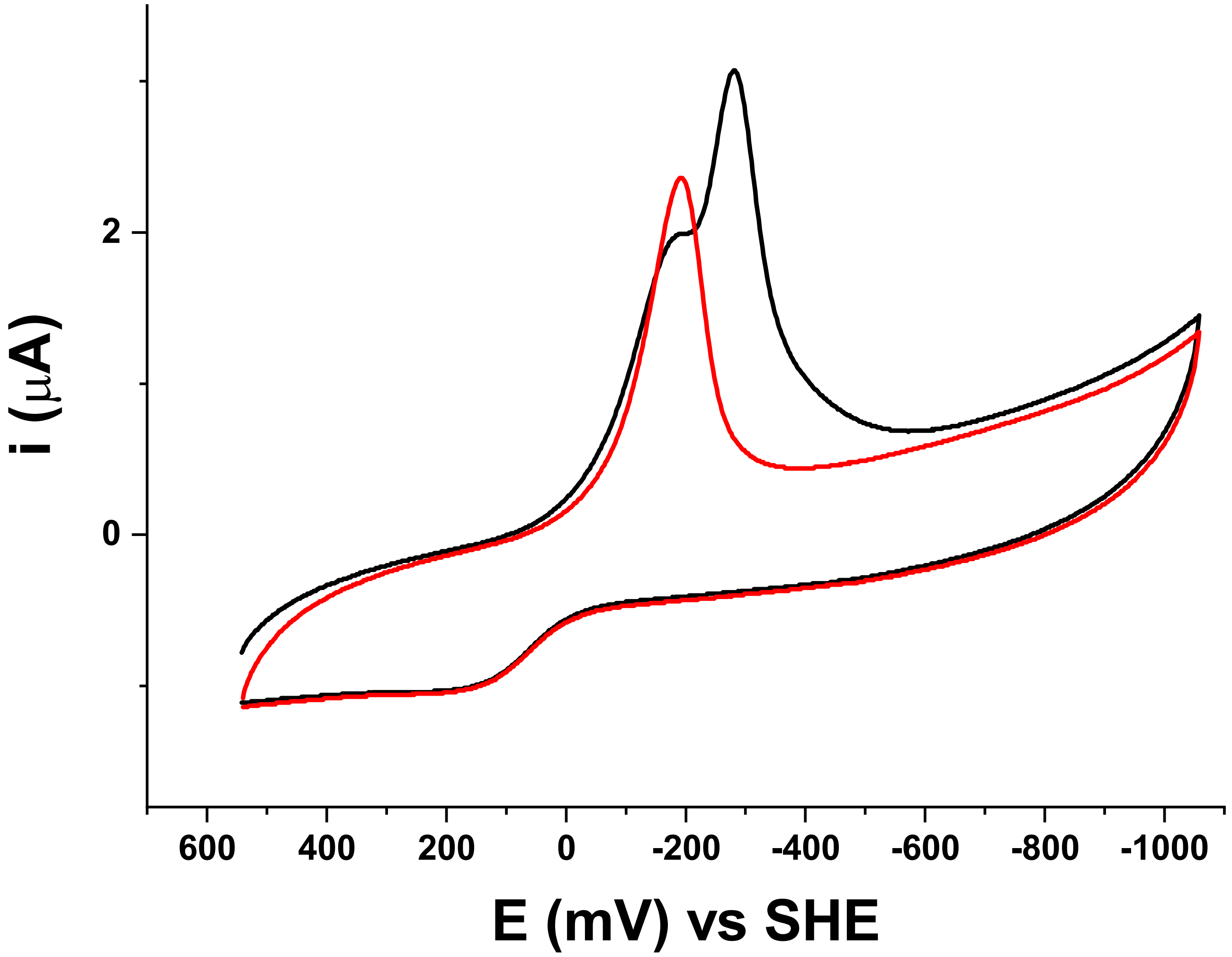

Supplementary Figure 13. Cyclic voltammetry of 0.4:1 HS^-:Fe^{3+} glutathione (40 mM glutathione, 0.8 mM Na_2S, 2 mM $FeCl_3$) in H_2O, pH 8.6. The potential sweep was applied in two consecutive steps. In accordance with the square wave voltammograms, the first cathodic sweep (black) detected two peaks, at -0.2 and -0.29 V, attributable to the mononuclear species and to [2Fe-2S] glutathione, respectively. The first anodic sweep revealed only a single anodic peak indicative (+0.50 V) of the mononuclear species. A second potential sweep (red) revealed a single cathodic peak indicative of the mononuclear species at -0.12 V, consistent with the reductive degradation of the [2Fe-2S] cluster.

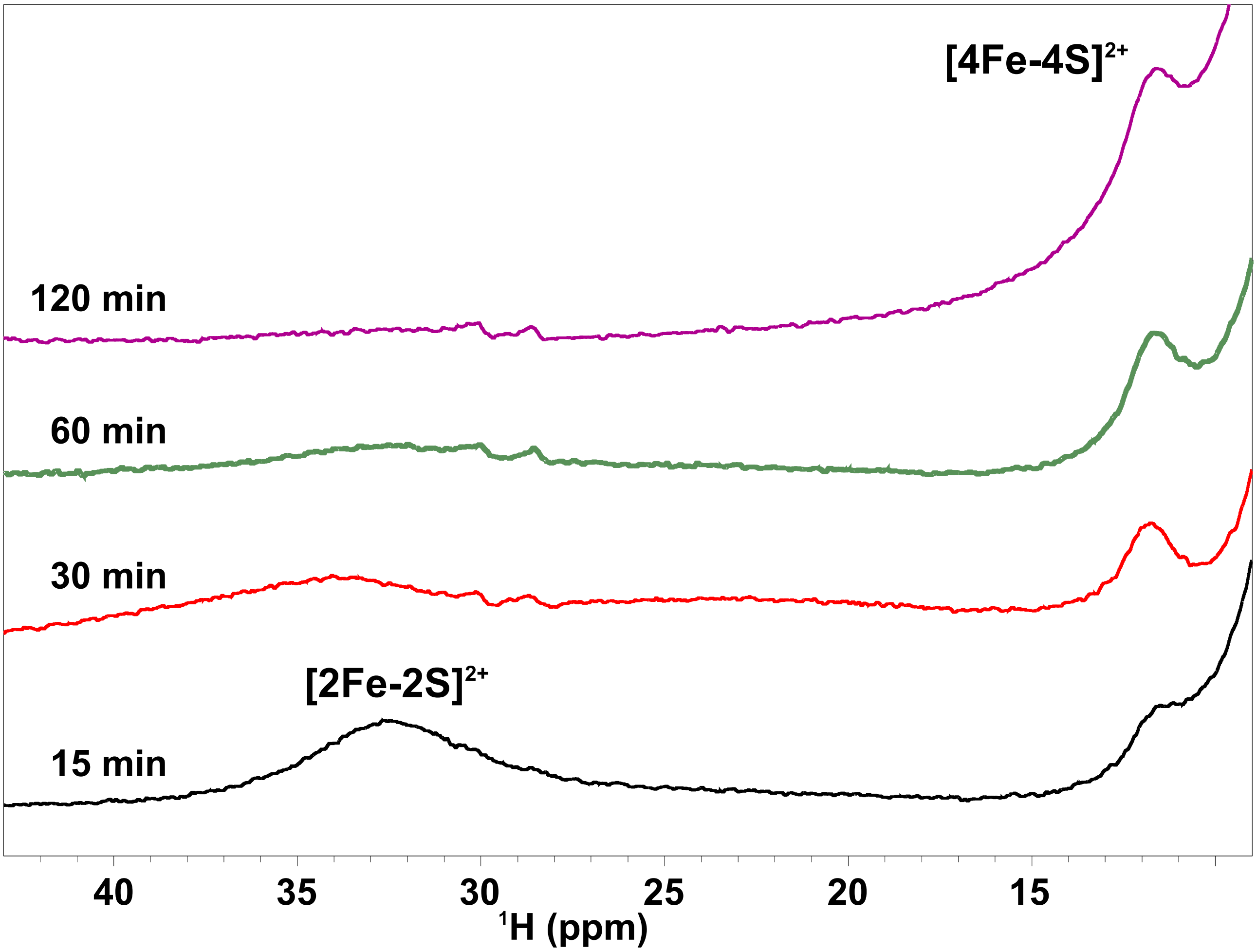

Supplementary Figure 14. Paramagnetic ^{1}H NMR spectra of 0.4:1 HS^-:Fe^{3+} glutathione (150 mM glutathione, 0.75 mM Na_2S, 1.87 mM $FeCl_3$) in D_2O, pD 8.6 over time. Upfield region. Initially (black spectrum), the resonance at 33 ppm corresponding to $[2Fe\text{-}2S]^{2+}$ glutathione was the major resonance detected in the spectral range, together with traces of $[4Fe\text{-}4S]^{2+}$ cluster (resonance at 11.8 ppm). Over time, the signal at 11.8 ppm increased and only traces of the broad resonance at 33 ppm ($[2Fe\text{-}2S]^{2+}$) were detected. Small resonances at 28.6 and 30.11 ppm are also detected (red, green and purple spectrum for 30, 60 and 120 minutes, respectively). The data suggested the reduction of the $[2Fe\text{-}2S]^{2+}$ cluster by means of exceeding glutathione with subsequent assembly of 2 $[2Fe\text{-}2S]^{+}$ units into a $[4Fe\text{-}4S]^{2+}$. During this process, the peak corresponding to the mononuclear, rubredoxin-like species stayed nearly unchanged (see **Fig. S15**).

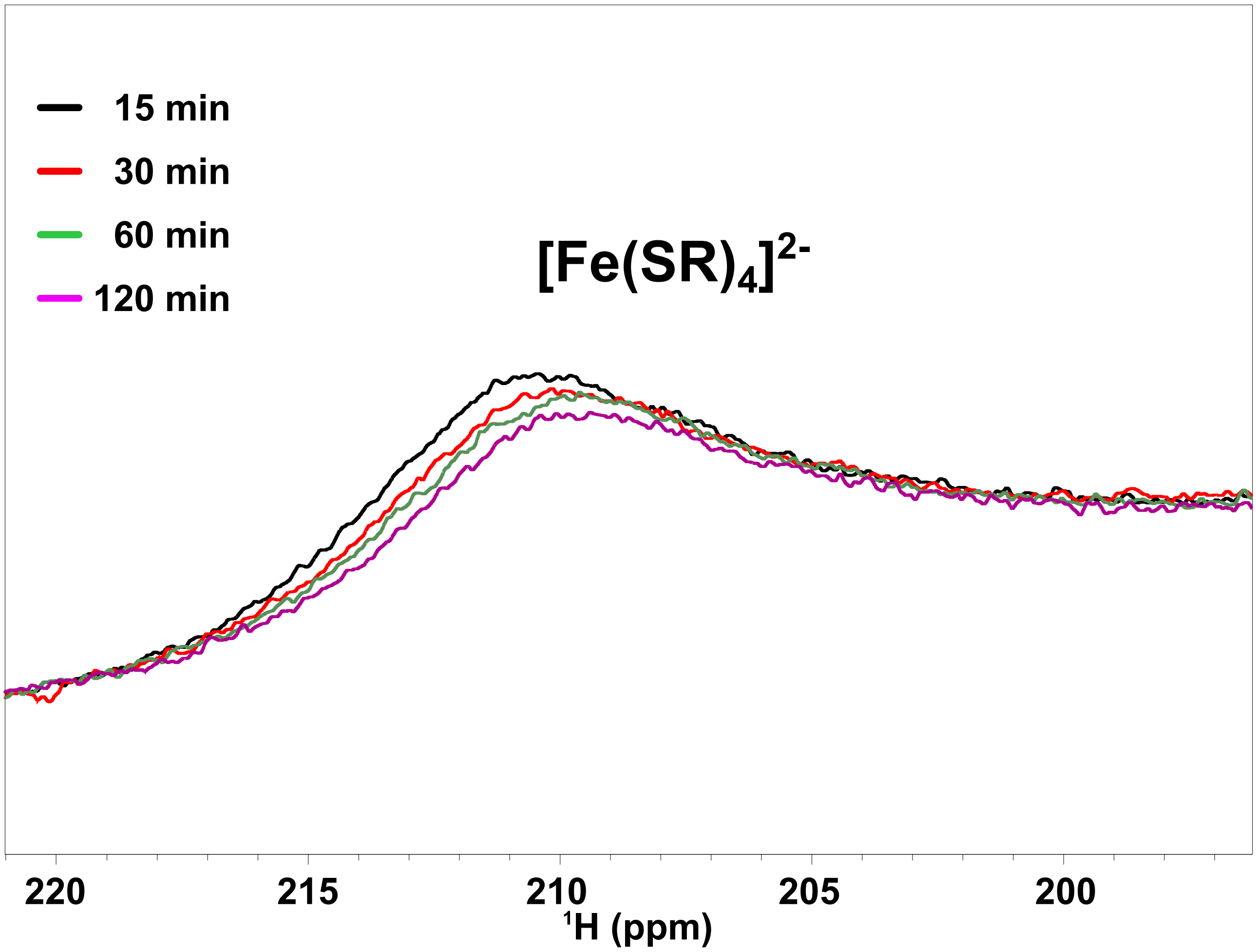

Supplementary Figure 15. Downfield region of paramagnetic ^{1}H NMR spectra of 0.4:1 HS^-:Fe^{3+} glutathione (150 mM glutathione, 0.75 mM Na_2S 1.87 mM $FeCl_3$) in D_2O, pD 8.6 over time. The resonance indicative of the reduced mononuclear species underwent a small decrease in intensity (black, red, green and purple represent 15, 30, 60 and 120 min, respectively). The same sample showed the presence of a $[2Fe\text{-}2S]^{2+}$ by a resonance at 33.3 pm, converting over the time into a $[4Fe\text{-}4S]^{2+}$in the upfield region (see **Fig. S14**).

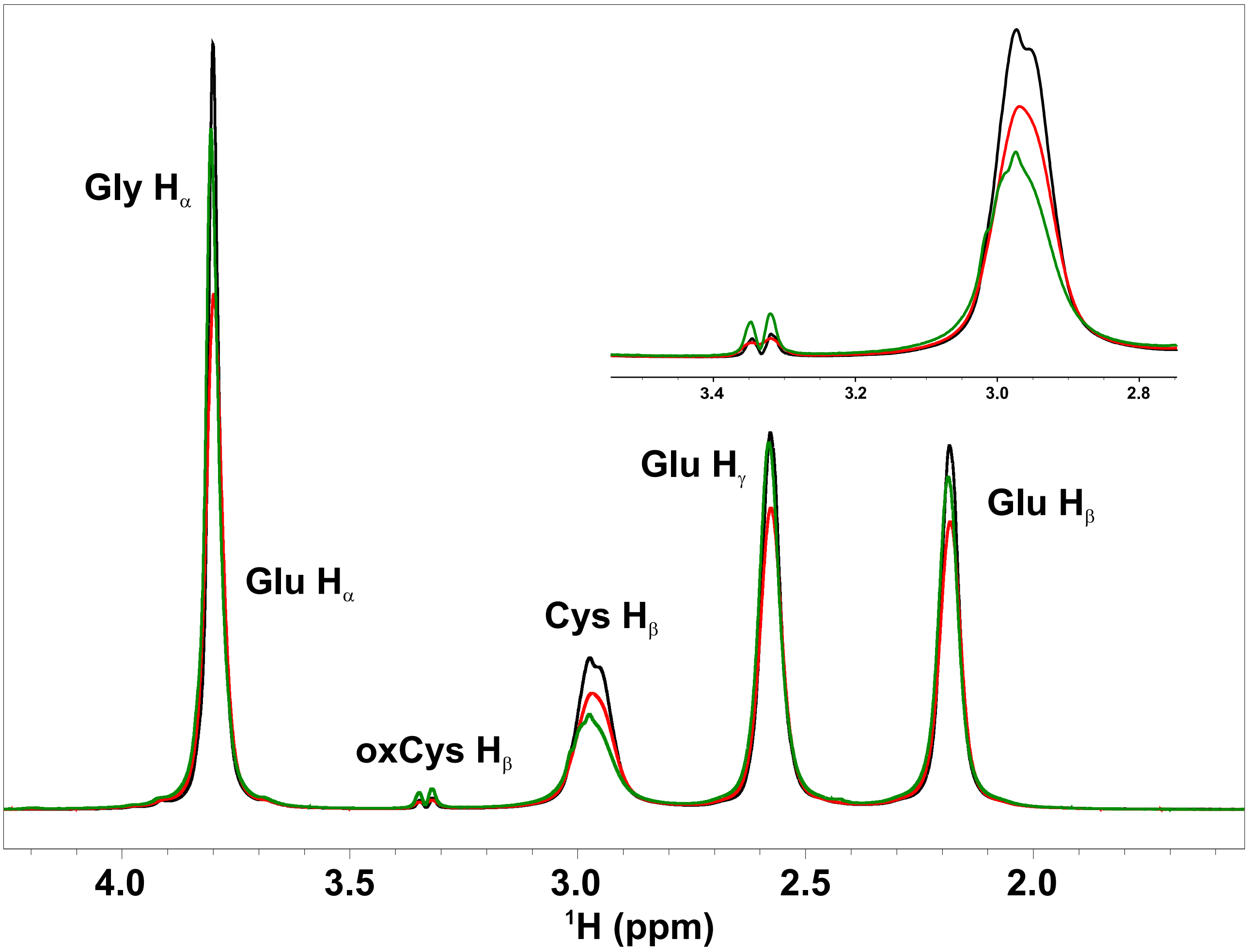

Supplementary Figure 16. ^1H NMR spectra of 0.4 HS^-:Fe^{3+} glutathione (150 mM glutathione, 0.75 mM Na_2S, 1.87 mM $FeCl_3$) in D_2O, pD 8.6 over time. Black (top), red (middle), and green (bottom) curves indicate spectra recorded immediately, 30 min, and 1 h after mixing. The resonances corresponding to oxidized Cys H_β (3.34 and 3.01 ppm, the latter buried by the broad Cys H_β resonance at 2.96 ppm) showed an increase in intensity over time.

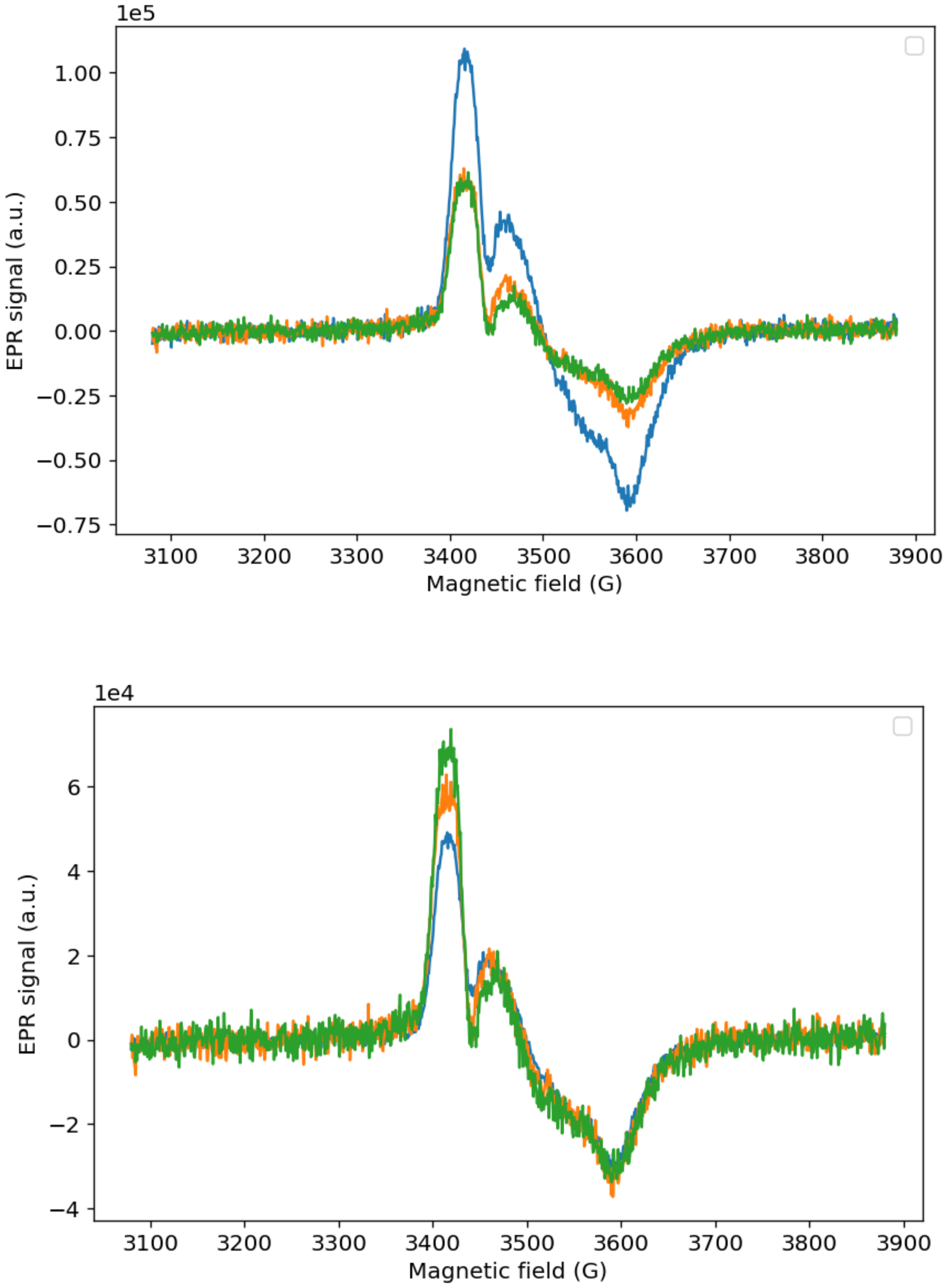

Supplementary Figure 17. EPR spectra of a solution containing 150 mM glutathione, 2.775 mM Na_2S, 7.5 mM $FeCl_3$ in H_2O, pH 8.6 at 20 K 15 minutes (blue), 3 hours (orange) and 24 hours (green) after mixing. (Top). The overall intensity decreases with time and the proportion of the two species changes. In the bottom figure, the same spectra were scaled so that the intensity corresponding to $[2Fe\text{-}2S]^+$ cluster (signal at 3515 G, g=1.96) is constant. This allows evidencing the relative increase upon time of the axial EPR active species (3420 G, g = 2.02) that is thus tentatively attributed to a reduced $[4Fe\text{-}4S]^{1+}$ cluster.

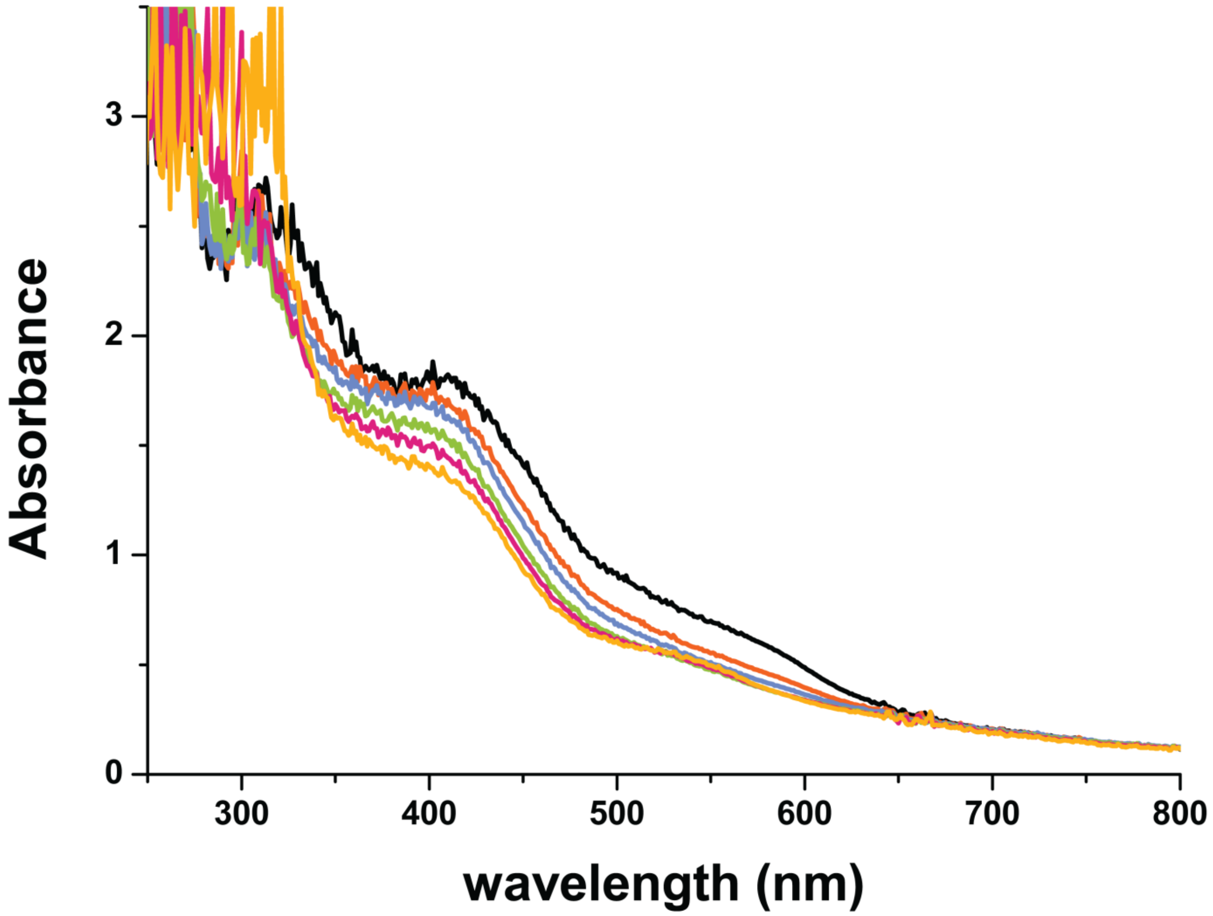

Supplementary Figure 18. UV-visible absorption spectra of 1:1 HS^{-}:Fe^{3+} glutathione (40 mM glutathione, 0.5 mM Na_2S, 0.5 mM $FeCl_3$) in H_2O, pH 8.6 over time. Soon after mixing (black, top), the absorption bands at 420 and 450 nm were observed, indicative of the presence of a $[2Fe\text{-}2S]^{2+}$ cluster. The fact that the absorption at 420 nm was more intense than that of 450 nm additionally suggested the presence of $[4Fe\text{-}4S]^{2+}$ cluster (contribution at 410 nm) in the mixture. Within 1 h, the band at 450 nm decreased in intensity and only a broad band at 410 nm remained (orange). Spectra from top to bottom are of 0 h, 1 h, 2 h, 4 h, 8 h, and 16 h after mixing.

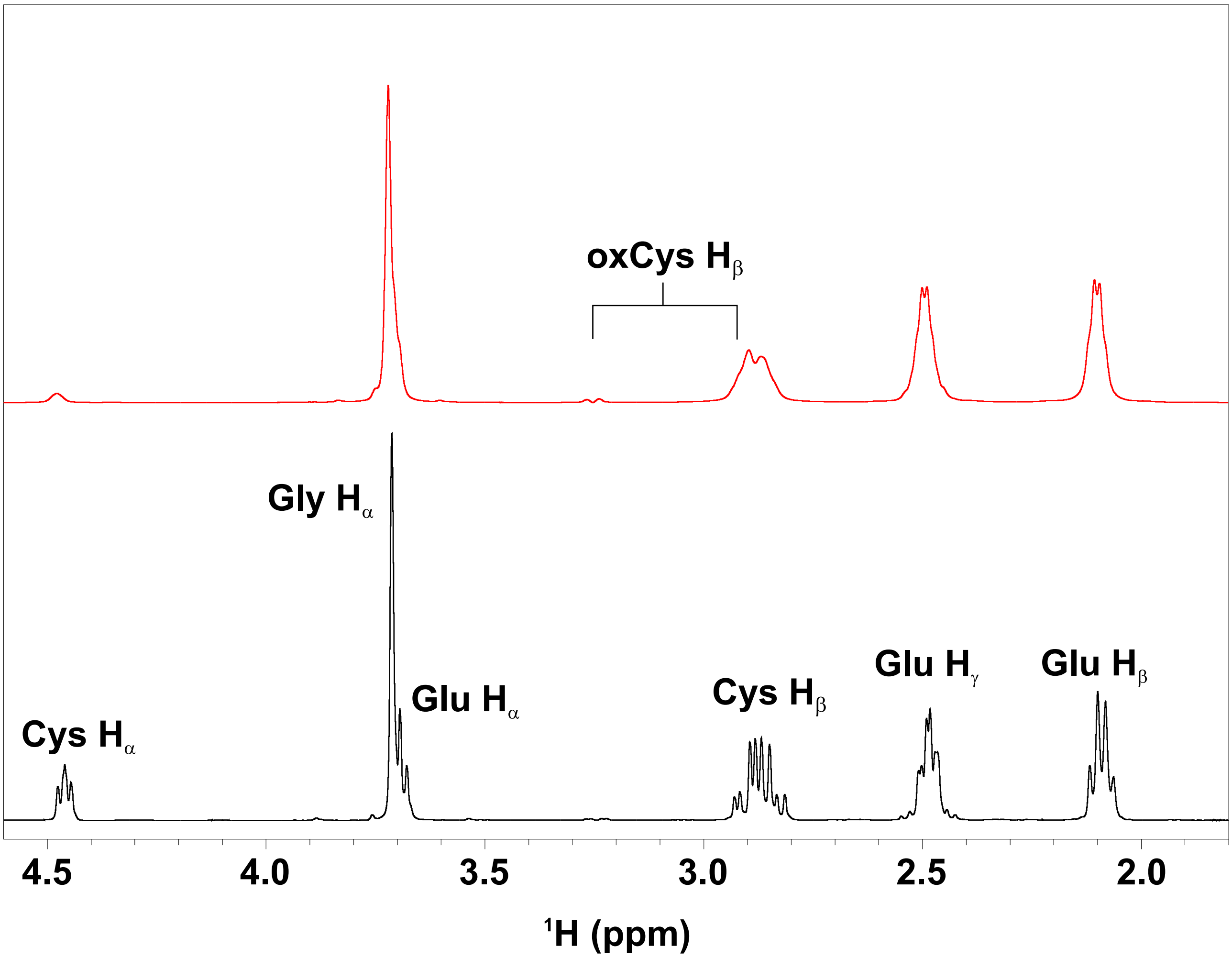

Supplementary Figure 19. ^{1}H NMR spectra of glutathione (black, 150 mM glutathione) and a solution of 1:1 HS^{-}:Fe^{3+} glutathione (red, 150 mM glutathione, 1.87 mM Na_2S and 1.87 mM $FeCl_3$) in D_2O, pD 8.6. Upon mixing the components, broadening affected all of the ^{1}H resonances of glutathione to a lesser extent with respect to what observed for the mononuclear complex and the mixture mononuclear/$[2Fe\text{-}2S]^{2+}$ (**Fig. S1** and **S7**, respectively): in particular the resonance of Cys H_{β} residue (2.83 ppm) appeared the most affected by the broadening. Nevertheless, the ratio of the integrals of the Glu $H_{\alpha/\beta}$ and Cys H_{β} resonances increased to 0.75 for $[4Fe\text{-}4S]^{2+}$ glutathione, whereas the same ratio was incalculable for mononuclear and $[2Fe\text{-}2S]^{2+}$ glutathione because their Cys H_{β} resonances were broadened beyond the detection limit. For comparison, the ratio of resonances Glu $H_{\alpha/\beta}$ / Cys H_{β} was 1 for the non-metallated form. The intensity of the signal corresponding to Cys H_{α} (4.42 ppm) was influenced by the NMR water suppression pulse.

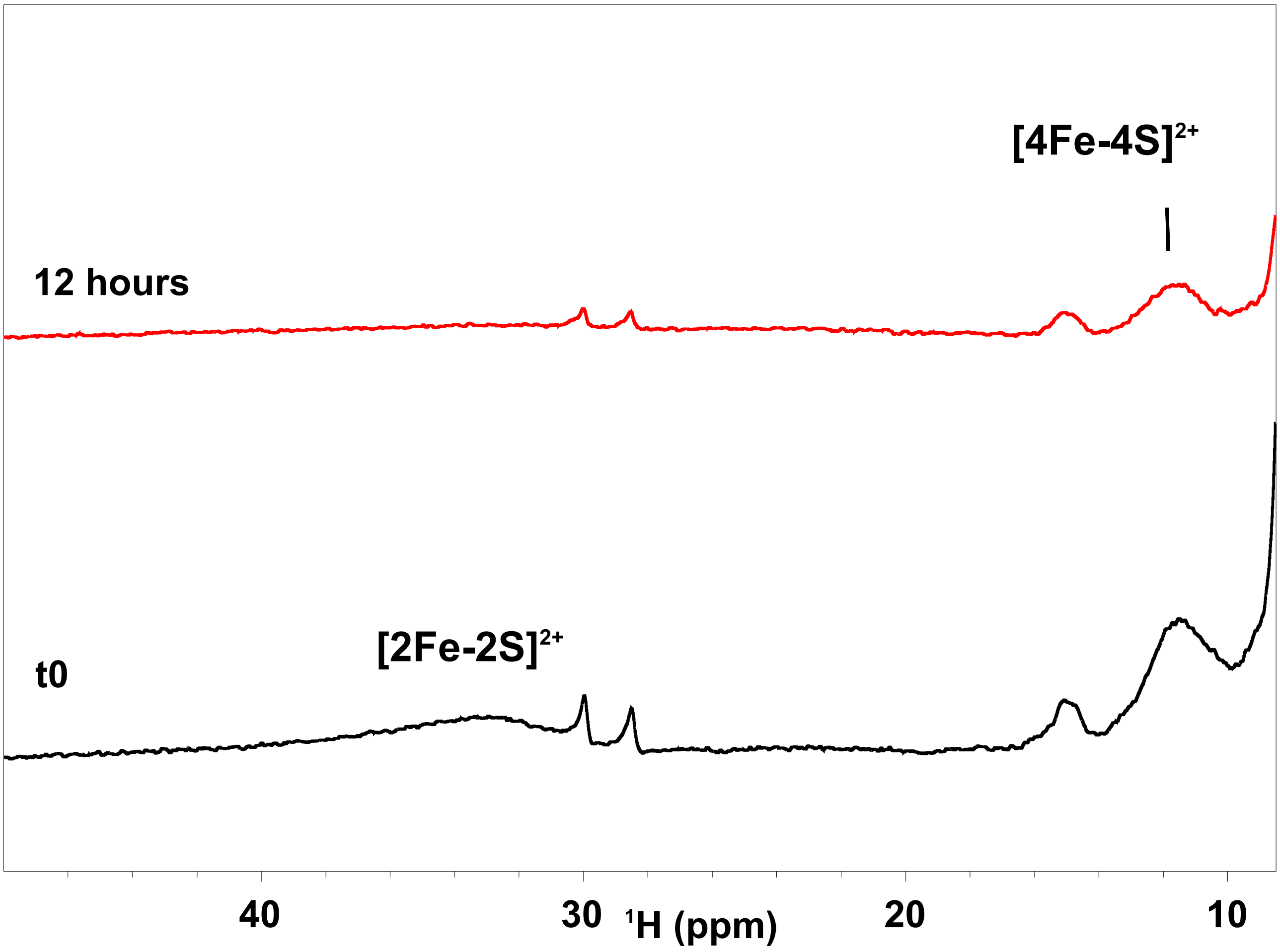

Supplementary Figure 20. Paramagnetic ^{1}H NMR spectra of 1:1 HS^{-}:Fe^{3+} glutathione (150 mM glutathione, 7.5 mM Na_2S, 7.5 mM $FeCl_3$) in D_2O, pD 8.6 over time. The solution initially gave rise to a mixture of iron-sulfur clusters (black, bottom). The broad band at 33.3 ppm indicated $[2Fe\text{-}2S]^{2+}$ glutathione, and the resonances at 11.8, 28 and 30 ppm were of $[4Fe\text{-}4S]^{2+}$. After 12 h (red, top), the resonance corresponding to $[2Fe\text{-}2S]^{2+}$ greatly diminished whereas the $[4Fe\text{-}4S]^{2+}$ and a resonance at 15.0 ppm were still detected. (See **Fig. S21** for the spectral region related to the mononuclear species). The resonance at 15.0 ppm can be attributed to an Fe-S intermediate species.

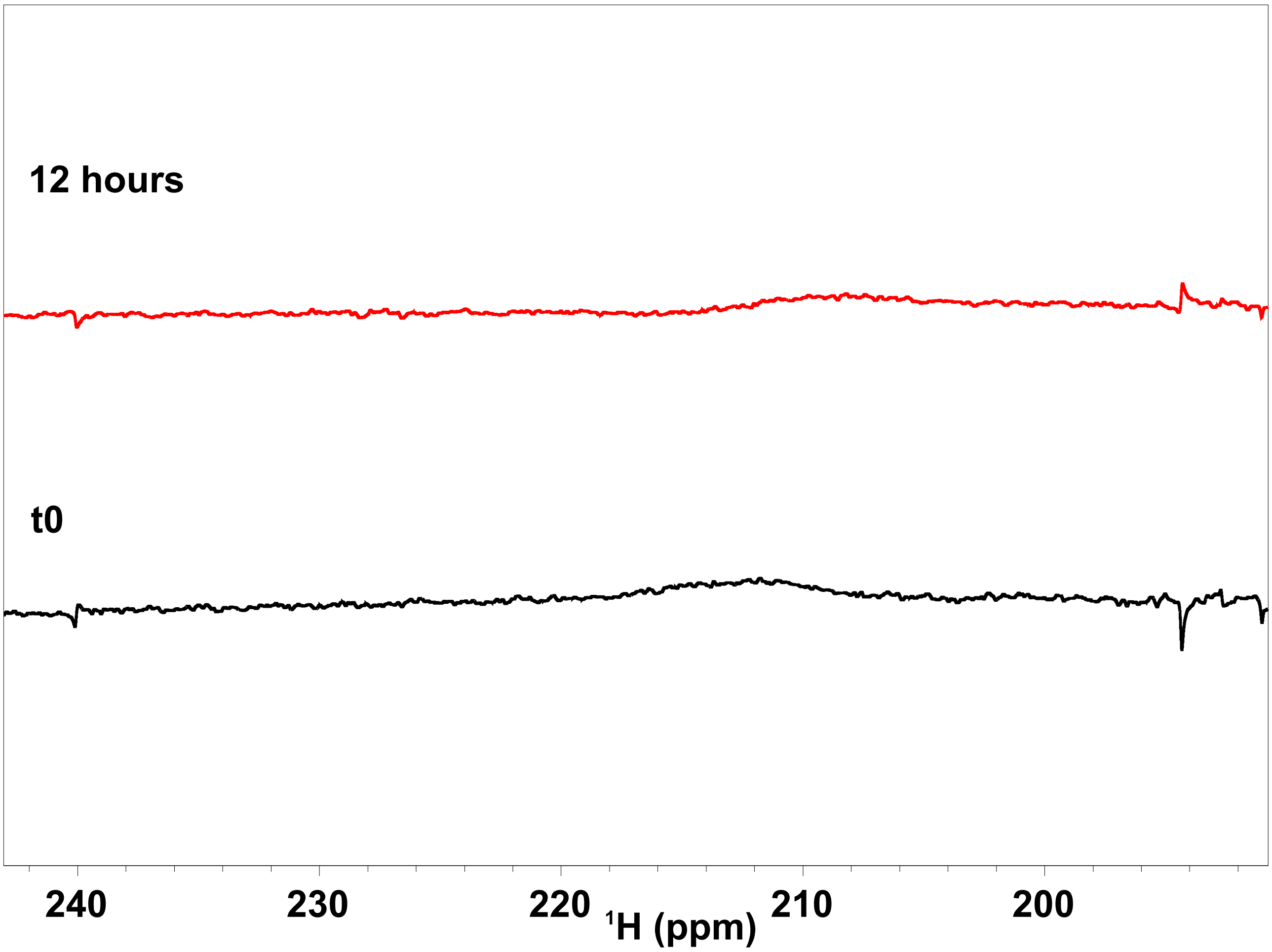

Supplementary Figure 21. Paramagnetic ^{1}H NMR spectra of 1:1 $HS^{-}:Fe^{3+}$ glutathione (150 mM glutathione, 7.5 mM Na_2S, 7.5 mM $FeCl_3$) in D_2O, pD 8.6. In addition to the iron-sulfur clusters detected in the upfield paramagnetic region (see **Fig. S20**), the solution of 1:1 $HS^{-}:Fe^{3+}$ showed, immediately after mixing, traces of mononuclear species at 211 ppm (black, bottom). After 12 h (red, top), the intensity of the resonance is negligible.

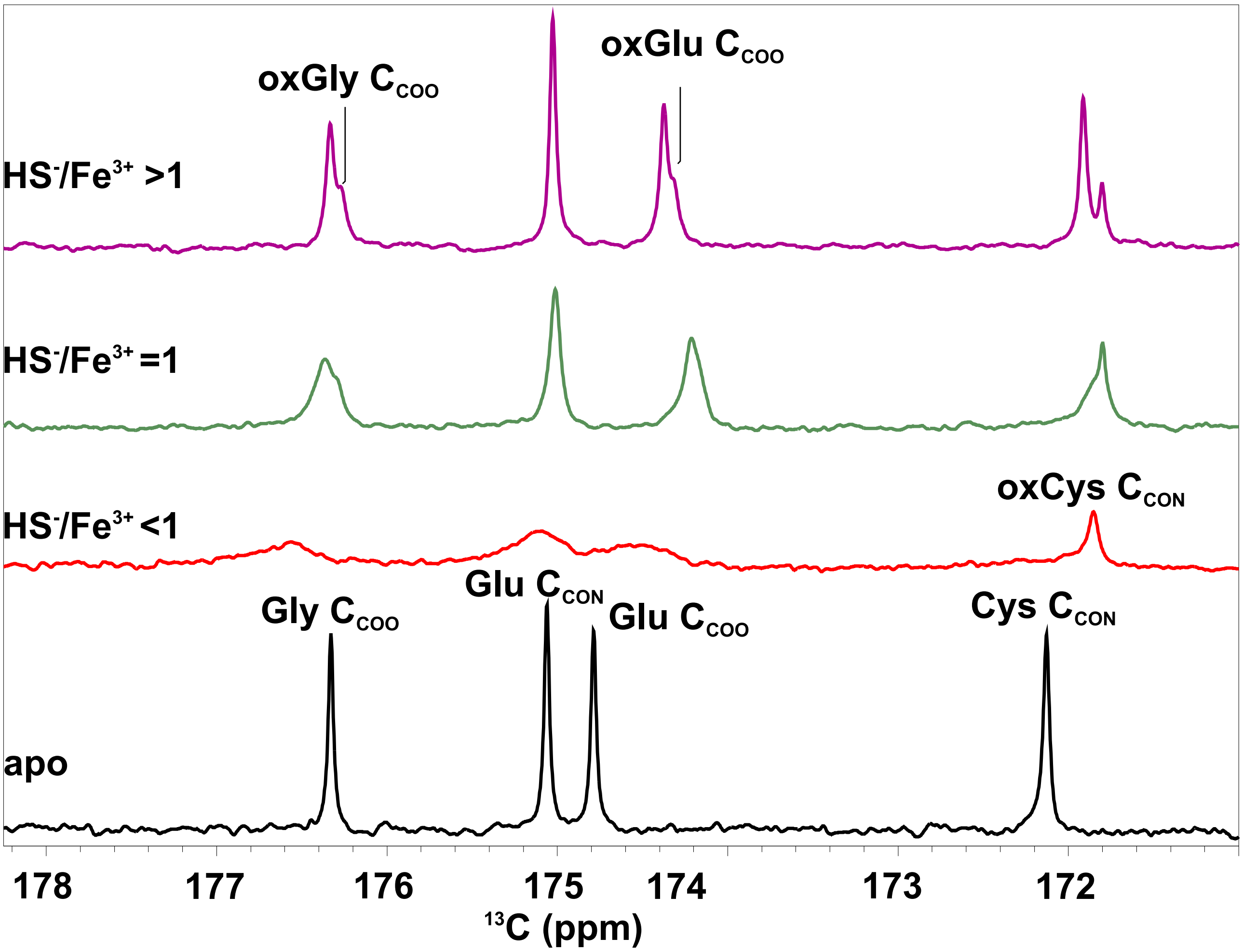

Supplementary Figure 22. ^{13}C NMR spectra of the carbonyl/carboxyl (CON/COO) region of 150 mM glutathione (black, bottom) alone and solutions containing 150 mM glutathione, 1.87 mM $FeCl_3$, and different concentrations of Na_2S in D_2O, pD 8.6. When $HS^-/Fe^{3+} < 1$ (red, 2nd from bottom, 750 μM Na_2S, 0.4:1 HS^-:Fe^{3+}), fast ligand exchange was responsible for the strong broadening of all of the ^{13}C resonances of glutathione. Conversely, the resonance corresponding to oxCys $^{13}C_{CON}$ (171.8 ppm) was detected. At 1:1 HS^-:Fe^{3+} (green, 2nd from top, 1.87 mM Na_2S), the rate of ligand exchange decreased, as well as the broadening of the signals. The $^{13}C_{CON}$ and $^{13}C_{COO}$ resonances of glutathione were now clearly distinguishable. The shoulder on the left of the oxCys $^{13}C_{CON}$ resonance corresponded to broadened and reduced Cys $^{13}C_{CON}$. When $HS^-/Fe^{3+} > 1$ (mauve, top, 3.75 mM Na_2S, 2:1 HS^-:Fe^{3+}), the resonances of glutathione appeared sharp (Glu C_{CON} half peak width 5.75, 11.51 and 6.81 for HS^-/Fe^{3+} =2, 1, and 0.4, respectively), increased in intensity, and higher than the resonances related to oxidized glutathione. At this ratio, peaks related to oxGly $^{13}C_{COO}$ and oxGlu $^{13}C_{COO}$ could be detected.

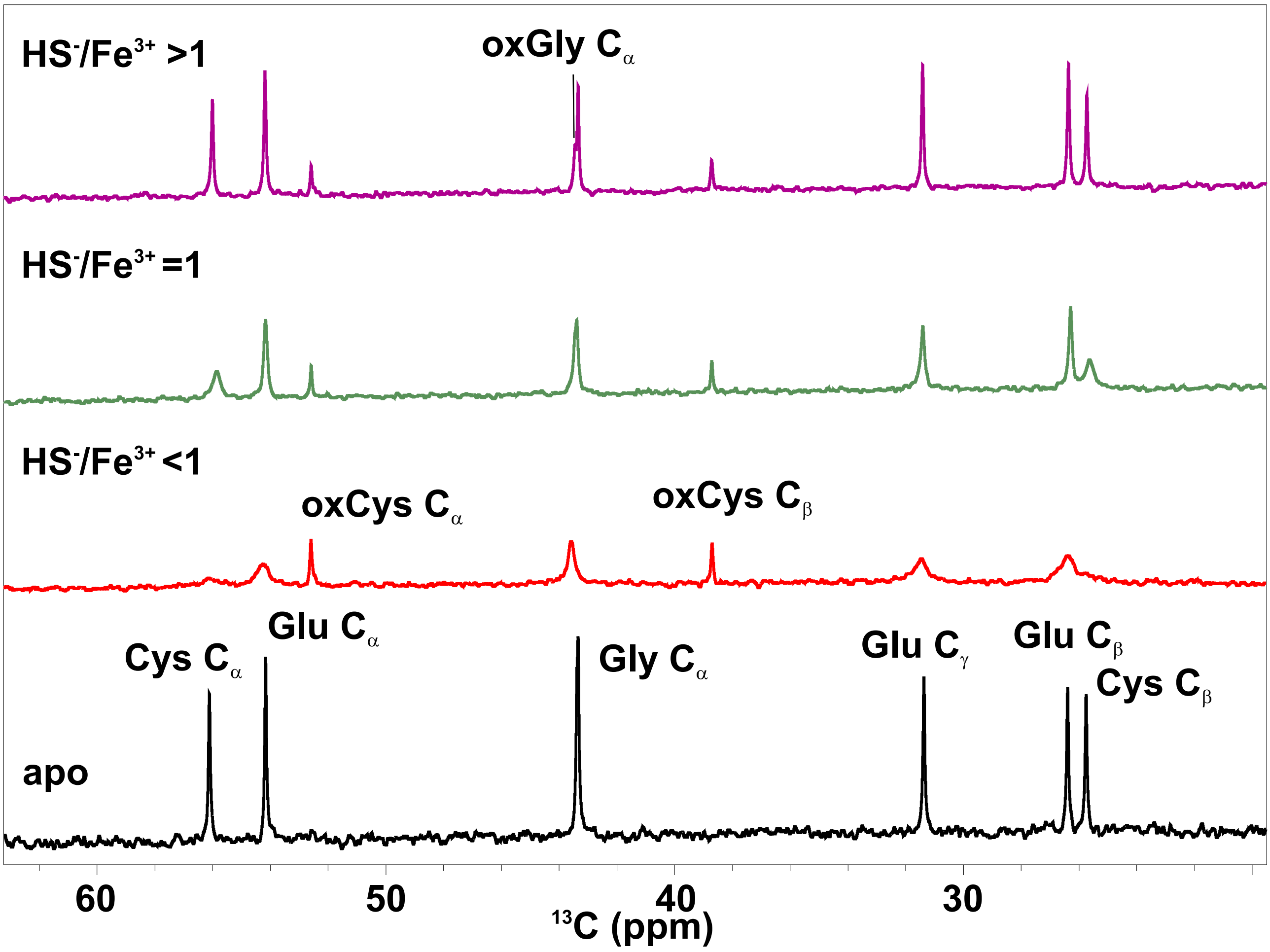

Supplementary Figure 23. The alkyl region of ^{13}C NMR spectra of 150 mM glutathione alone (black, bottom) and 150 mM glutathione, 1.87 mM $FeCl_3$ with different concentrations of Na_2S in D_2O, pD 8.6. When $HS^-/Fe^{3+} < 1$ (red, 2nd from bottom, 750 μM Na_2S, 0.4:1 $HS^-:Fe^{3+}$), fast ligand exchange strongly affected all of the ^{13}C resonances of glutathione, whereas the resonance of oxidized Cys $C_{\alpha,\beta}$ (38.7 and 52.6 ppm, respectively) could be detected. For example, the half-peak width of the Glu $^{13}C_\gamma$ resonance (5.9 Hz for apo glutathione, black, bottom) broadened to 48.7 Hz. At 1:1 $HS^-:Fe^{3+}$ (green, 2nd from top, 1.87 mM Na_2S), as effect of a slower ligand exchange a less intense broadening of the ^{13}C resonances was observed. As a result, all the resonances reduced their signal width (≈16 Hz) with respect to the apo-, non-metallated peptide. In particular, for Glu $^{13}C_\gamma$ resonance, the value decreased to 16.6 Hz. At this ratio, the resonance corresponding to oxGly $^{13}C_\alpha$ could be detected flanking Gly $^{13}C_\alpha$ resonance. When $HS^-/Fe^{3+} > 1$ (mauve, top, 3.75 mM Na_2S, 2:1 $HS^-:Fe^{3+}$), the half-peak width of Cys $^{13}C_{\alpha,\beta}$ resonances recovered almost entirely their relative intensity, and the line-widths were narrower than any other tested conditions containing HS^-.

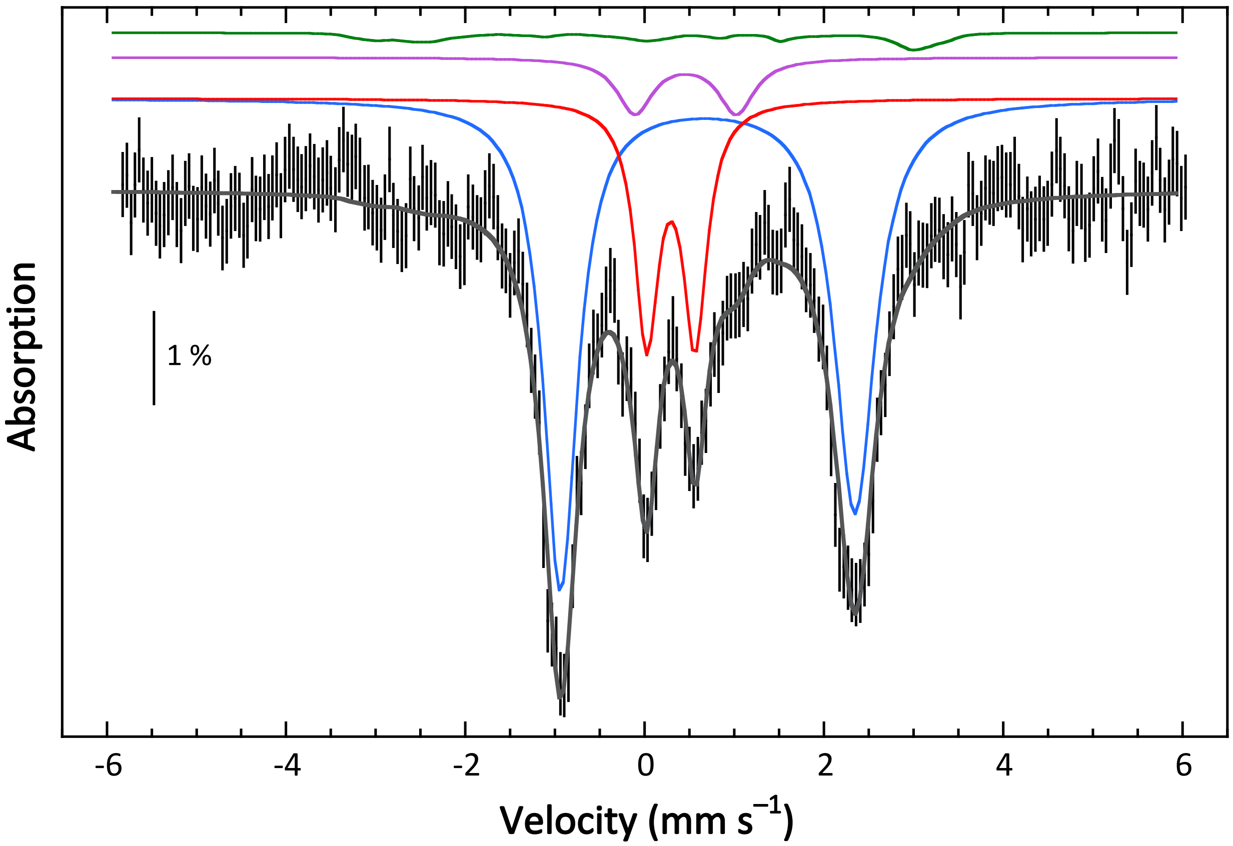

Supplementary Figure 24 Mössbauer spectra of the of 1:1 HS^{-}:Fe^{3+} glutathione (150 mM glutathione, 2 mM Na_2S and 2 mM $FeCl_3$) in H_2O, pH 8.6 at 4.2 K with a 0.06 T external magnetic field applied parallel to the γ-rays. The overlaid simulation (thick grey solid line) is the sum of four contributions displayed above the spectrum. The previously identified mononuclear ferrous complex (δ = 0.70 mm s^{-1} and ΔE_Q = 3.29 mm s^{-1}) and the all-ferric [2Fe-2S] cluster (δ = 0.29 mm s^{-1} and ΔE_Q = 0.54 mm s^{-1}) are shown in blue and red, respectively. The absorption close to 1 mm s^{-1} can be reproduced by a doublet (mauve solid line) presenting nuclear parameters consistent with diamagnetic $[4Fe\text{-}4S]^{2+}$ clusters (δ = 0.46 mm s^{-1} and ΔE_Q = 1.13 mm s^{-1}). Lastly, a small contribution of $[2Fe\text{-}2S]^{+}$ clusters (green solid line) is considered. All the parameters associated to this latter species are fixed to those of the $[2Fe\text{-}2S]^{+}$ cluster of the two-iron ferredoxin of *Clostridium pasteurianum*[5]. These fours species account for 66, 22, 8 and 4 % of the total iron content, respectively.

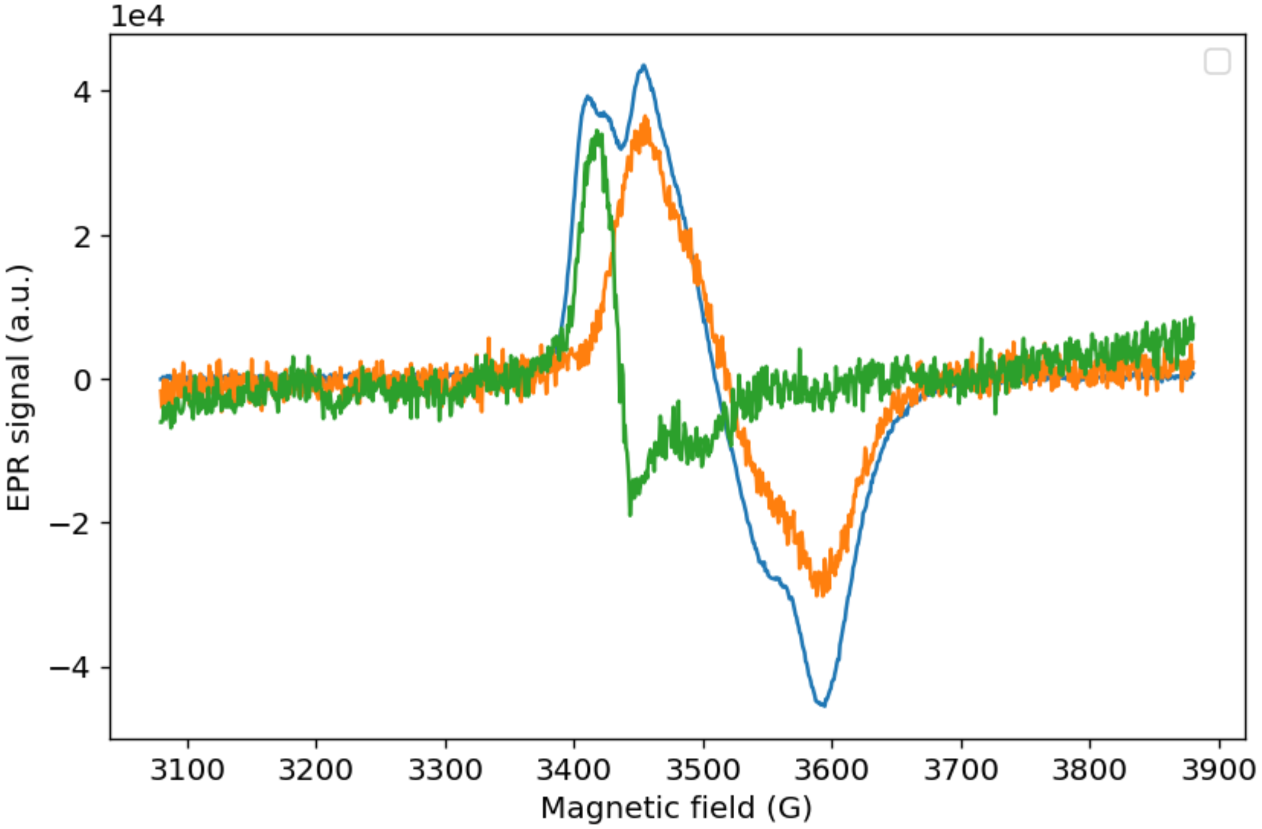

Supplementary Figure 25 EPR spectra of a solution containing 150 mM glutathione, 7.5 mM Na_2S, 7.5 mM $FeCl_3$ in H_2O, pH 8.6 15 minutes after mixing at 20 K (blue, zoom 0.15x); of a solution containing 40 mM glutathione, 2 mM Na_2S, 2 mM $FeCl_3$ in H_2O, pH 8.6 15 minutes after mixing at 20 K (orange); of a solution containing 15 mM glutathione, 0.74 mM Na_2S, 2 mM $FeCl_3$ in H_2O, and 100 mM carbonate, pH 8.6 at 10 K (green). A mixture of $[2Fe\text{-}2S]^{+}$ (3515 G, g=1.96) and $[4Fe\text{-}4S]^{+}$ (3420 G, g=2.02) could be observed, as previously for solutions containing $[Fe(SR)_4]^{2-}/[2Fe\text{-}2S]^{2+}$ mixtures (see **Fig. S11**), but with a different ratio.

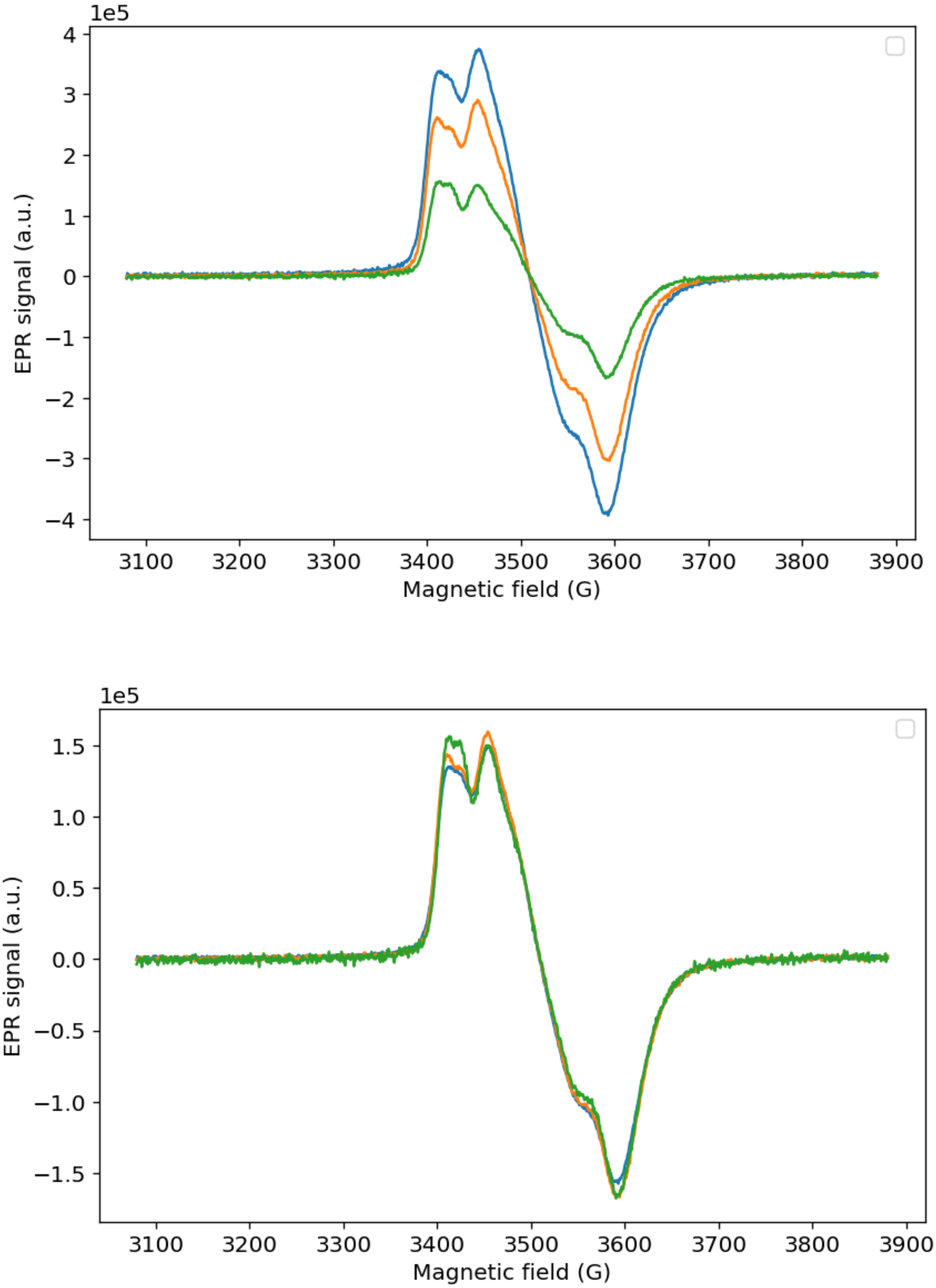

Supplementary Figure 26. EPR spectra of a solution of 150 mM glutathione, 7.5 mM Na_2S, 7.5 mM $FeCl_3$ in H_2O, pH 8.6 immediately after mixing (top) at 10K (blue), 20 K (orange) and 40 K (green). A mixture of $[2Fe\text{-}2S]^+$ (3515 G, g=1.96) and $[4Fe\text{-}4S]^+$ (3420 G, g=2.02) could be observed. The same EPR spectra have been scaled (bottom) so that the intensity of the $[2Fe\text{-}2S]^+$ species is constant, thus revealing slight difference in relaxation properties between these two species between at 10 K and 40 K.

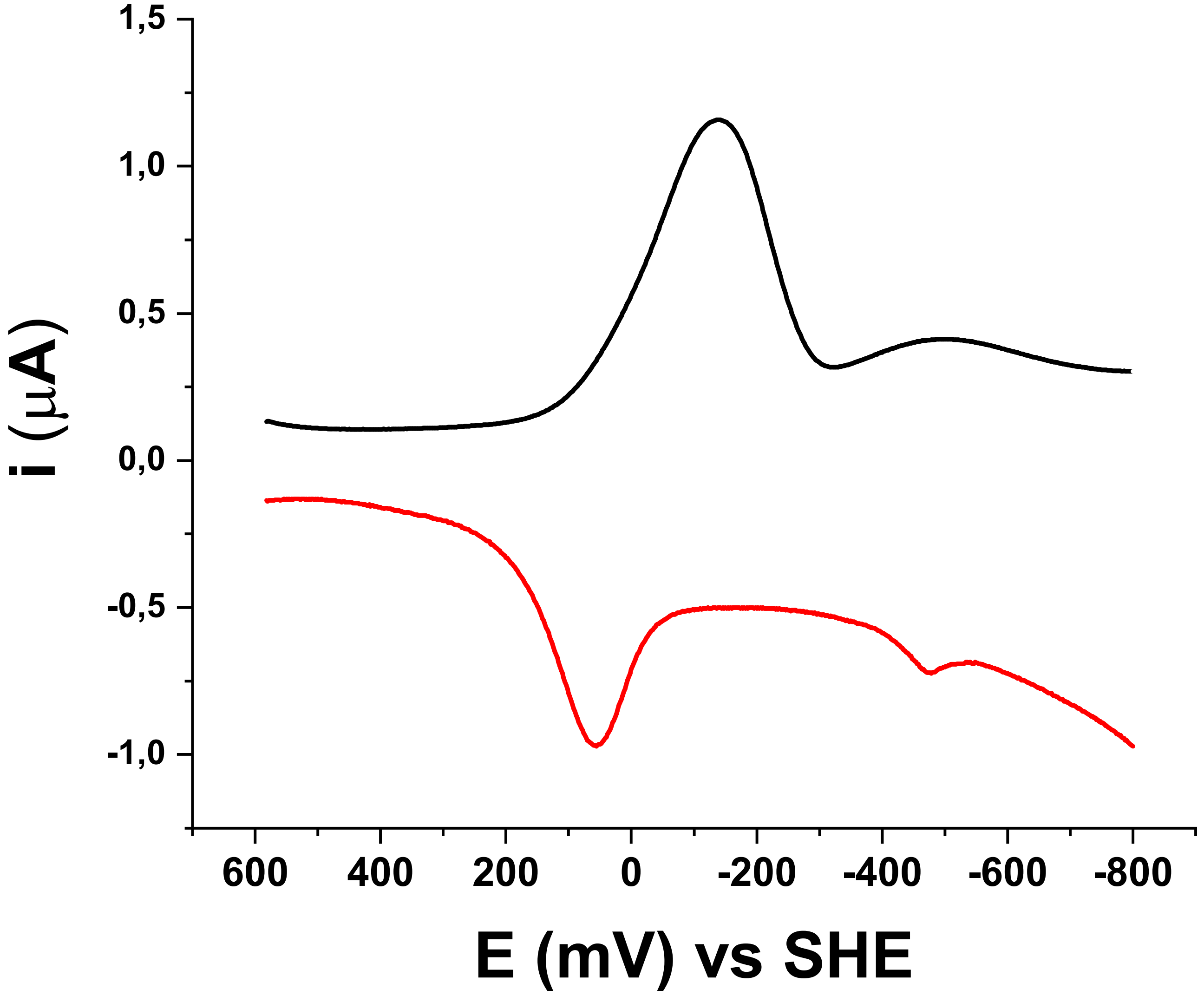

Supplementary Figure 27. Square wave voltammetry of 1:1 HS^{-}:Fe^{3+} glutathione (40 mM glutathione, 2 mM Na_2S, 2 mM $FeCl_3$) in H_2O, pH 8.6. Cathodic square wave voltammetry (black curve) showed one peak at -0.50 ± 0.01 V (broad), with the corresponding anodic signal at -0.48 ± 0.01 V (well shaped), attributed to a $[4Fe\text{-}4S]^{2+}$ cluster. Furthermore, signals attributed to a mononuclear species can be observed at -0.13 ± 0.01 V (E_{cp}) and +0.05 ± 0.01 V (E_{ap}). Under these conditions, the mononuclear species showed a formal reduction potential of E°' = -0.04 ± 0.01 V.

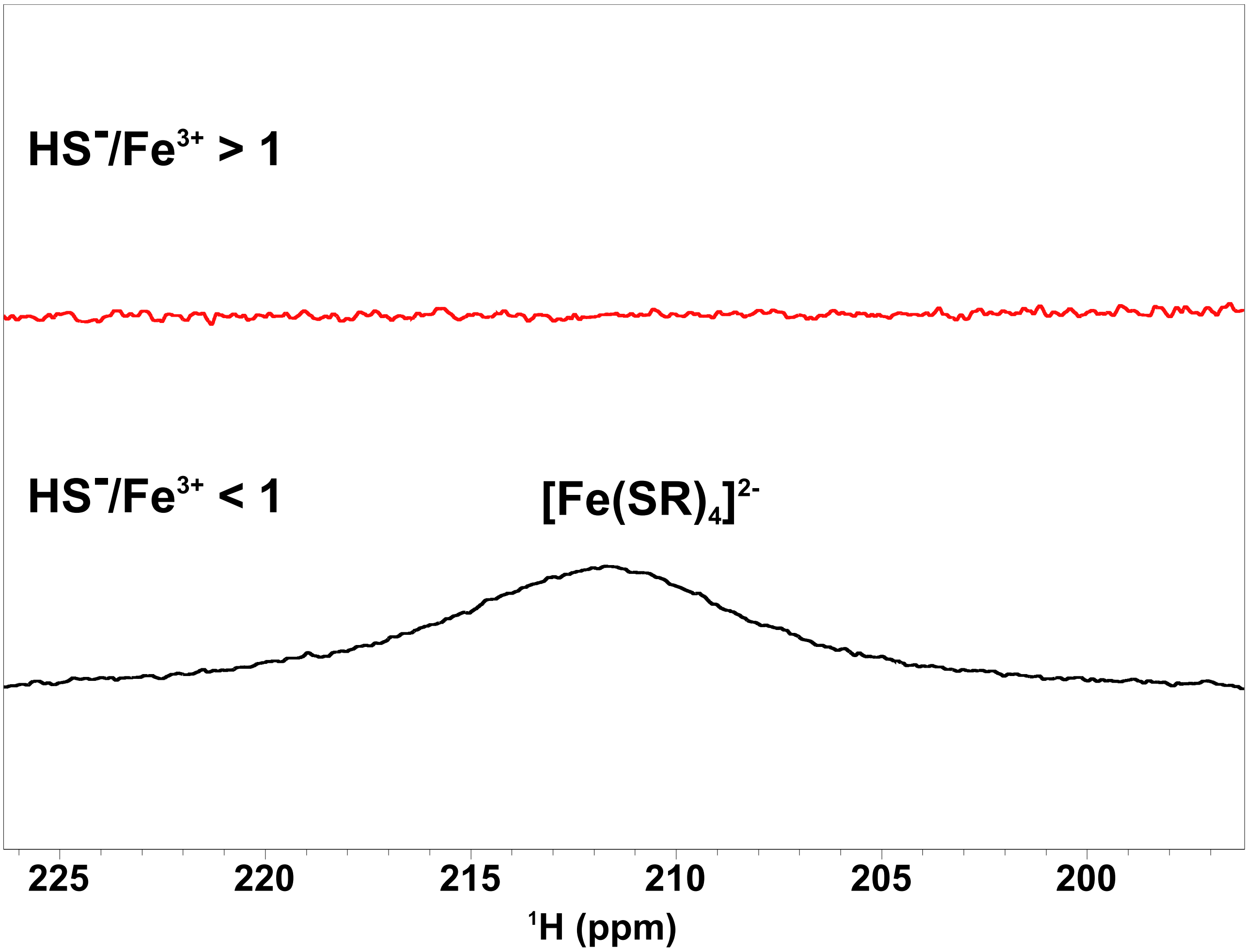

Supplementary Figure 28. Comparison of paramagnetic ^{1}H NMR spectra in D_2O, pD 8.6 with two different HS^-:Fe^{3+} ratios. The solution containing 2:1 HS^-:Fe^{3+} (3.75 mM Na_2S, 1.87 mM $FeCl_3$, 150 mM glutathione, pD 8.6), conditions that suggest formation of the putative $[6Fe\text{-}9S]^{2-}$ cluster, showed no resonances in the paramagnetic region indicative of the presence of the reduced mononuclear species (red, top). The solution containing 0.4:1 HS^-:Fe^{3+} (750 μM Na_2S and 1.87 mM $FeCl_3$, 150 mM glutathione, pD 8.6) conditions in which $[2Fe\text{-}2S]^{2+}$ cluster is formed, showed the presence of the reduced mononuclear species with a broad resonance at 211 ppm (black, bottom).

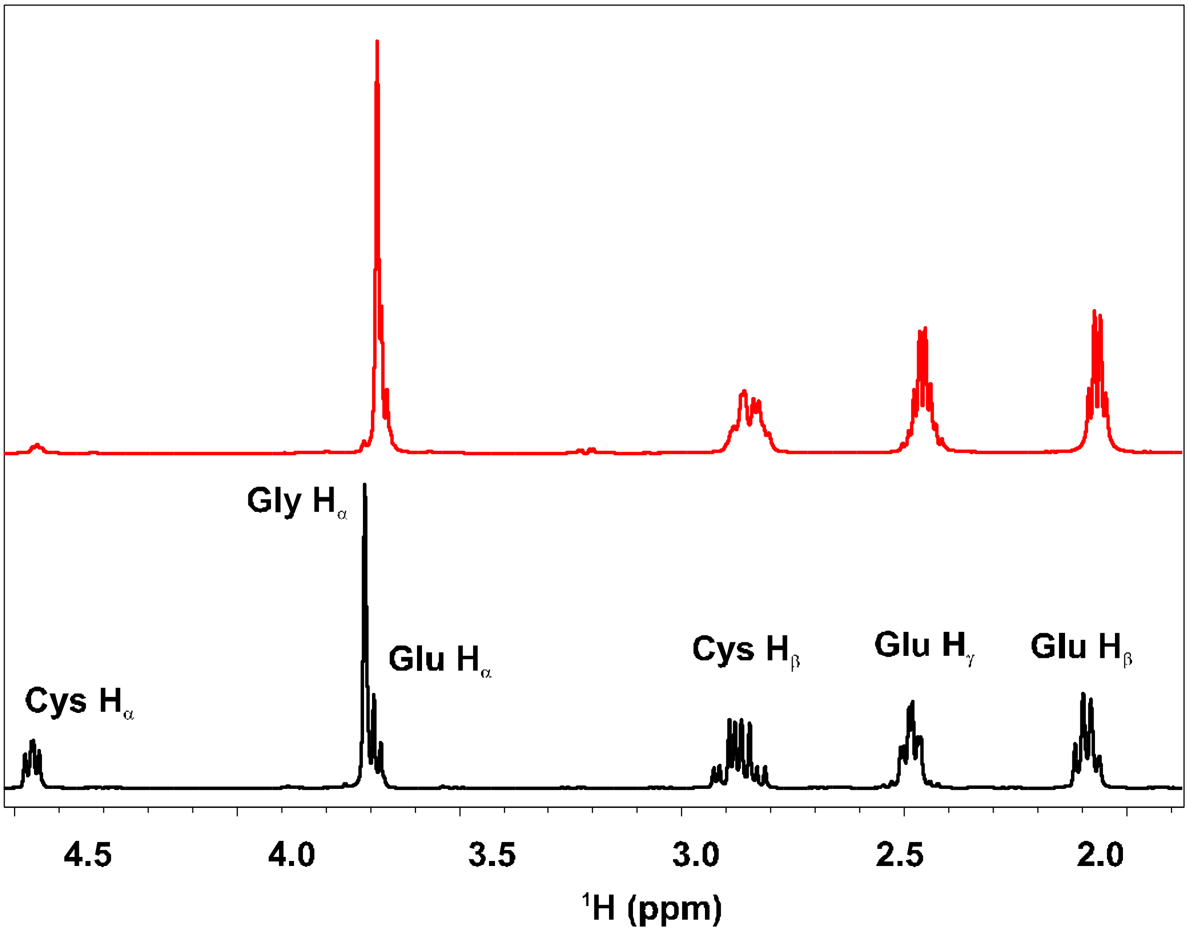

Supplementary Figure 29. ^{1}H NMR spectra of 150 mM glutathione (black, bottom) and 2:1 HS^-:Fe^{3+} glutathione (150 mM glutathione, 3.75 mM Na_2S, 1.87 mM $FeCl_3$) (red, top) in D_2O, pD 8.6. With respect to the lower HS^-:Fe^{3+} ratios investigated (see **Fig. S1, S7** and **S19**), all of the resonances related to glutathione fully recovered signal multiplicity. However, compared to the ^{1}H NMR spectra of non-metallated glutathione, the intensities of the resonances were not fully recovered, likely still due to ligand exchange.

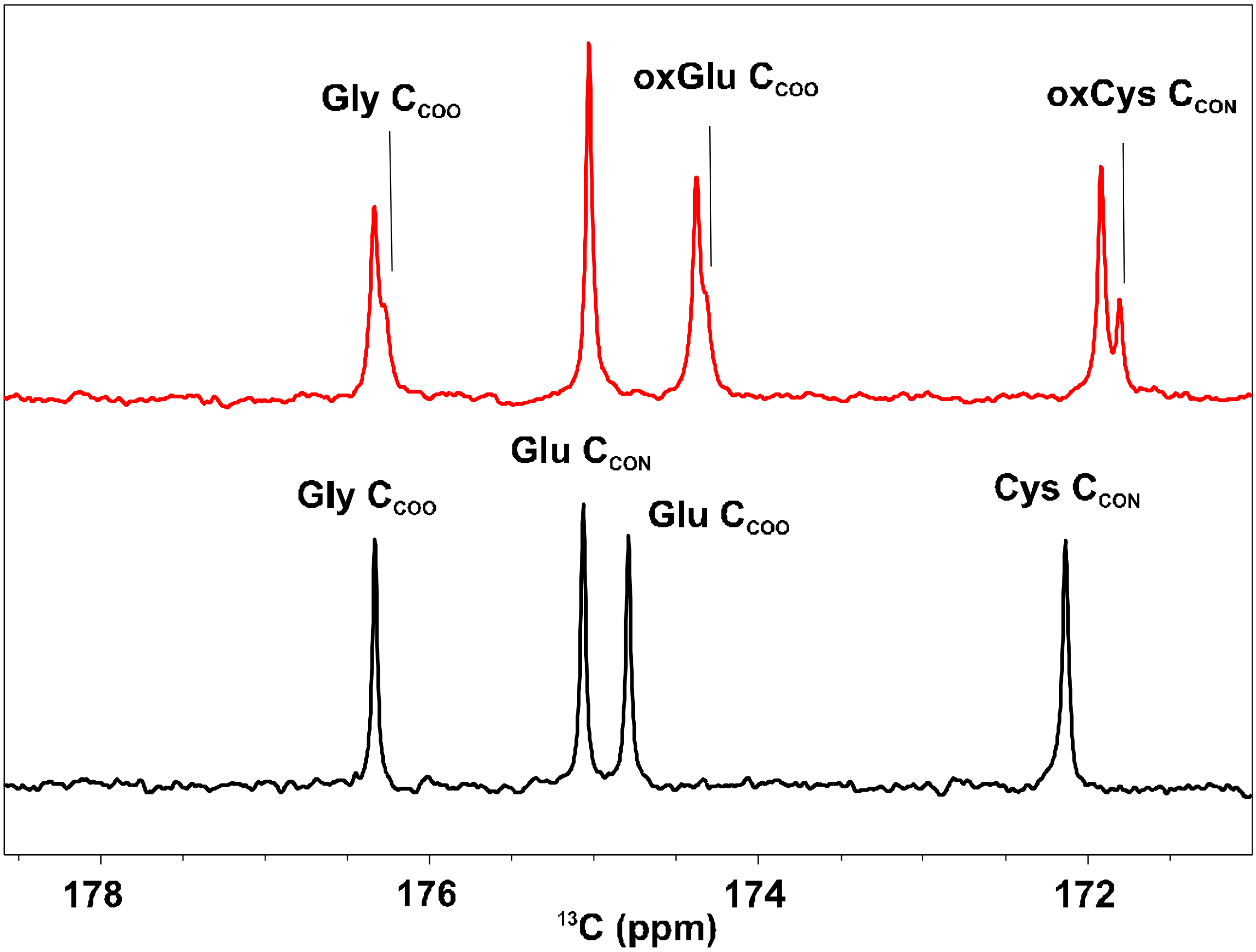

Supplementary Figure 30. ^{13}C NMR spectra of the carbonyl/carboxy (CON/COO) region of 150 mM glutathione (black, bottom) and 2:1 HS^-:Fe^{3+} glutathione (150 mM glutathione, 3.75 mM Na_2S, 1.87 mM $FeCl_3$) (red, top) in D_2O, pD 8.6. Differently from the $[2Fe\text{-}2S]^{2+}$ and $[4Fe\text{-}4S]^{2+}$samples, the Cys $^{13}C_{CON}$ (171.91), Glu $^{13}C_{COO}$ (174.37), Glu $^{13}C_{CON}$ (175.02), and Gly $^{13}C_{COO}$ (176.33 ppm) resonances of the putative $[6Fe\text{-}9S]^{2-}$ glutathione experienced little broadening. A weak upfield shift of the resonances (-0.05 ppm for Gly $^{13}C_{COO}$ and -0.12 ppm for Glu $^{13}C_{COO}$ and Cys $^{13}C_{COO}$) with respect to non-metallated glutathione was detected. Except for Glu $^{13}C_{CON}$, a less intense peak (about 1/3 of the intensity) was observed flanking each signal. All the new signals might be attributable to the ^{1}H nuclei of oxidized glutathione. The low intensity ratio between the reduced and the oxidized glutathione ^{13}C resonances (about 3 to 1) suggests still an impactful effect of ligand exchange on the signal intensity, even at high HS^-/Fe^{3+}ratios.

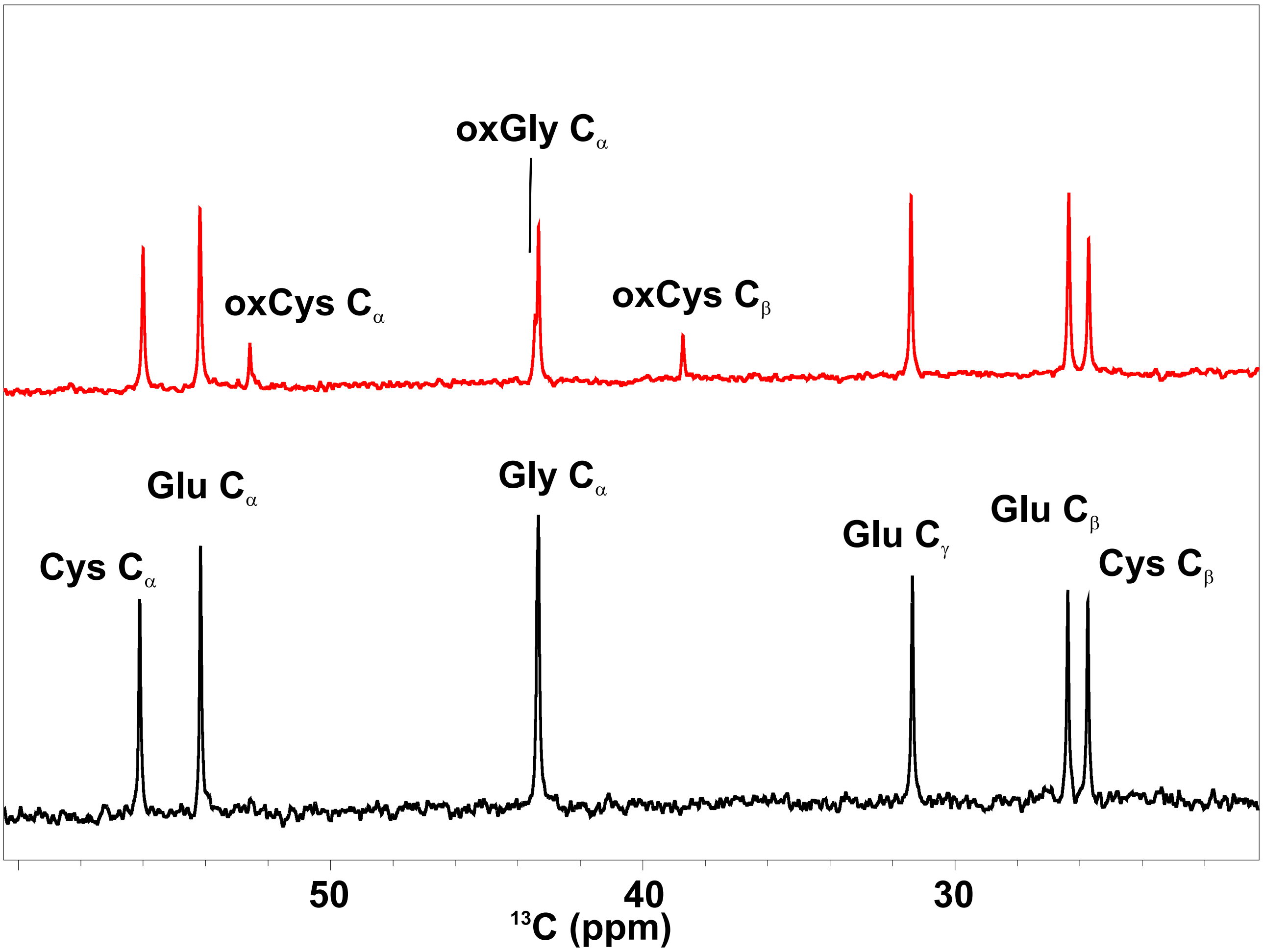

Supplementary Figure 31. Alkyl region of ^{13}C NMR spectra of 150 mM glutathione (black, bottom) and 2:1 HS^{-}:Fe^{3+} glutathione (150 mM glutathione, 3.75 mM Na_2S, 1.87 mM $FeCl_3$) (red, top) in D_2O, pD 8.6. [6Fe-9S]$^{2-}$ glutathione formed in solution exhibited little line broadening. ^{13}C resonances were at 25.71 (Cys $^{13}C_\beta$), 26.35 (Glu $^{13}C_\beta$), 31.40 (Glu $^{13}C_\gamma$), 43.33 (Glu $^{13}C_\alpha$), 54.18 (Glu $^{13}C_\alpha$), and 55.99 ppm (Cys $^{13}C_\alpha$). The sharper signals allowed for the detection of small upfield (-0.11 Cys $^{13}C_\alpha$, -0.03 ppm for Cys and Glu $^{13}C_\beta$) and downfield shifts (+0.04 ppm for Glu $^{13}C_\gamma$, +0.02 Glu $^{13}C_\alpha$) of the resonances with respect to glutathione-only. Signals related to oxCys $C_{\alpha,\beta}$ could be detected. Similarly to what observed in the $^{13}C_{CON}/_{COO}$ spectral region (**Fig. S30**) the low intensity ratio between the reduced and the oxidized glutathione alkyl ^{13}C resonances (about 3 to 1) suggests still an impactful effect of ligand exchange on the signal intensity, even at high HS^{-}/Fe^{3+}ratios.

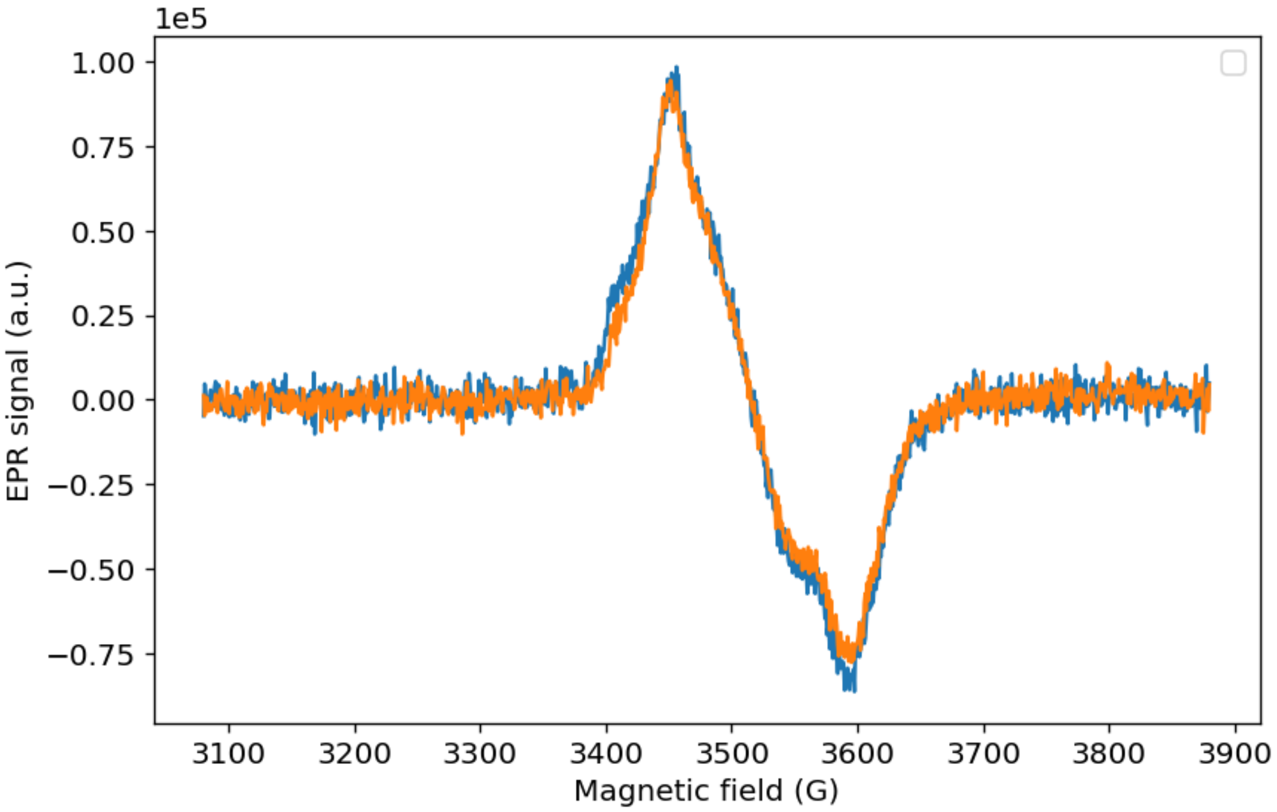

Supplementary Figure 32 EPR spectra of 150 mM glutathione, 3.75 mM Na_2S, 1.875 mM $FeCl_3$ in H_2O, pH 8.6 at 20 K immediately after mixing (blue) and 2.5 hours later (orange). A paramagnetic signal was detected at 3515 G (g = 2, corresponding to $[2Fe\text{-}2S]^+$ species). Spin counting demonstrated a minority contribution (11%) of this signal to the overall iron present in solution. The concentration of $[4Fe\text{-}4S]^+$ cluster was negligible in this sample (shoulder at 3420 G), contrary to solution containing $HS^-/Fe^{3+} < 1$. The signal corresponding to species $[2Fe\text{-}2S]^+$ did not decreased with time in this condition.

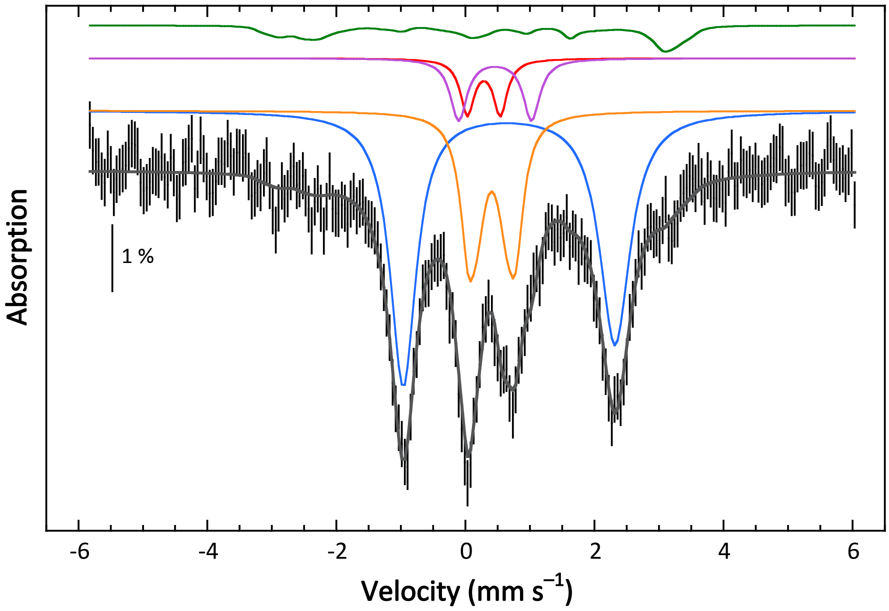

Supplementary Figure 33. Mössbauer spectrum of a solution of glutathione, HS^-, and Fe^{3+} (160 mM glutathione, 4 mM Na_2S, 2 mM $FeCl_3$) in H_2O, pH 8.6 at 4.2 K with a 0.06 T external magnetic field applied parallel to the γ-rays. Five different iron-sulfur clusters are considered to reproduce this spectrum. The starting ferrous, mononuclear complex (blue solid line) accounts for 54 % of the total iron content (δ = 0.68 mm s^{-1} and ΔE_Q = 3.28 mm s^{-1}). The magnetic contribution (absorption below –2 mm s^{-1} and at ≈3 mm s^{-1}) can be reproduced using the parameters of the reduced cluster of the two-iron, i.e. $[2Fe\text{-}2S]^+$, ferredoxin of *Clostridium pasteurianum* (green solid line). This $[2Fe\text{-}2S]^+$ cluster accounts for 8 % of the total iron content. The central part of the spectrum can be reproduced by considering four doublets. The first one is associated with an oxidized $[2Fe\text{-}2S]^{2+}$ cluster (δ = 0.28 mm s^{-1} and ΔE_Q = 0.51 mm s^{-1}, 6 %, red solid line) and the second to a $[4Fe\text{-}4S]^{2+}$ cluster (δ = 0.45 mm s^{-1} and ΔE_Q = 1.12 mm s^{-1}, 8 %, mauve solid line). Nuclear parameters for the $[Fe_6S_9(S^tBu)_2]^{4-}$ have been used to complete the simulation (24 %, orange solid line). Two doublets in a 2:1 ratio are considered with δ_1 = 0.41 mm s^{-1}, $\Delta E_{Q,1}$ = 0.72 mm s^{-1}, δ_2 = 0.40 mm s^{-1}, and $\Delta E_{Q,2}$ = 0.43 mm s^{-1}.

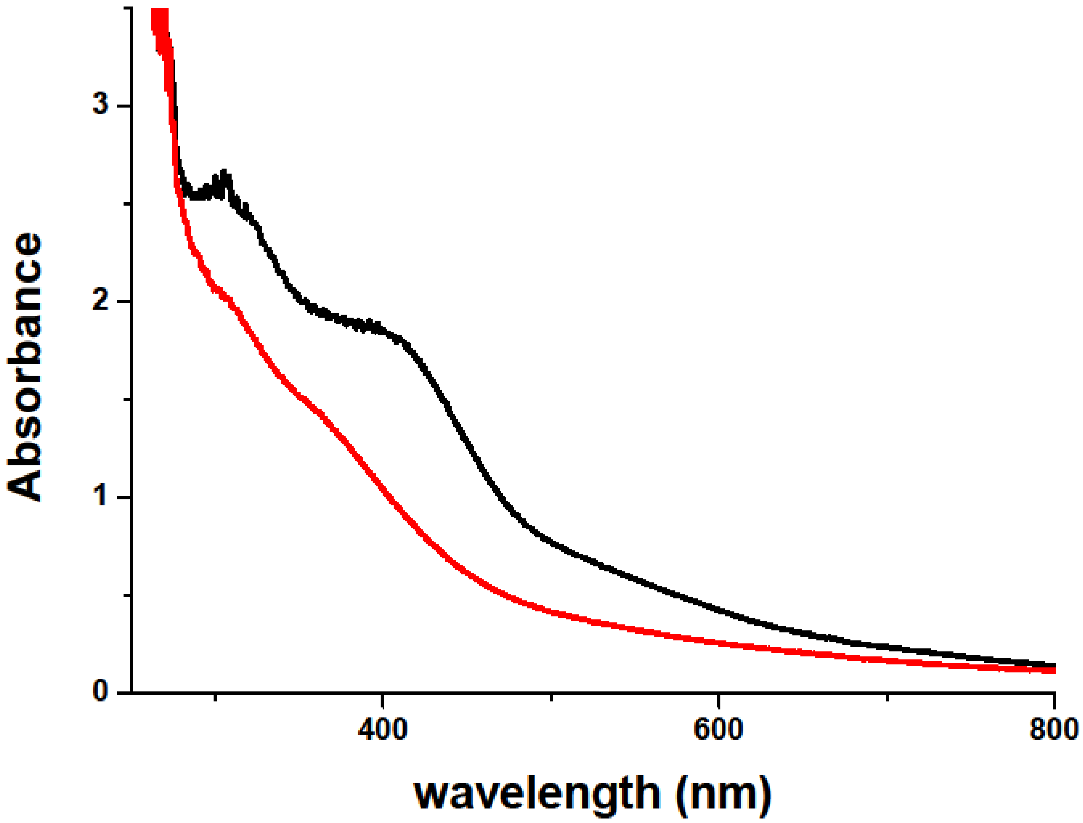

Supplementary Figure 34. UV-visible absorption spectra of 2:1 HS^{-}:Fe^{3+} glutathione (40 mM glutathione, 1 mM Na_2S, 0.5 mM $FeCl_3$) in H_2O, pH 8.6 before (black, top) and after (red, bottom) the addition of 125 μM sodium dithionite. The solution initially possessed a broad absorption band at 410 nm, corresponding to the putative $[6Fe\text{-}9S]^{2-}$ cluster (black, top).[11]

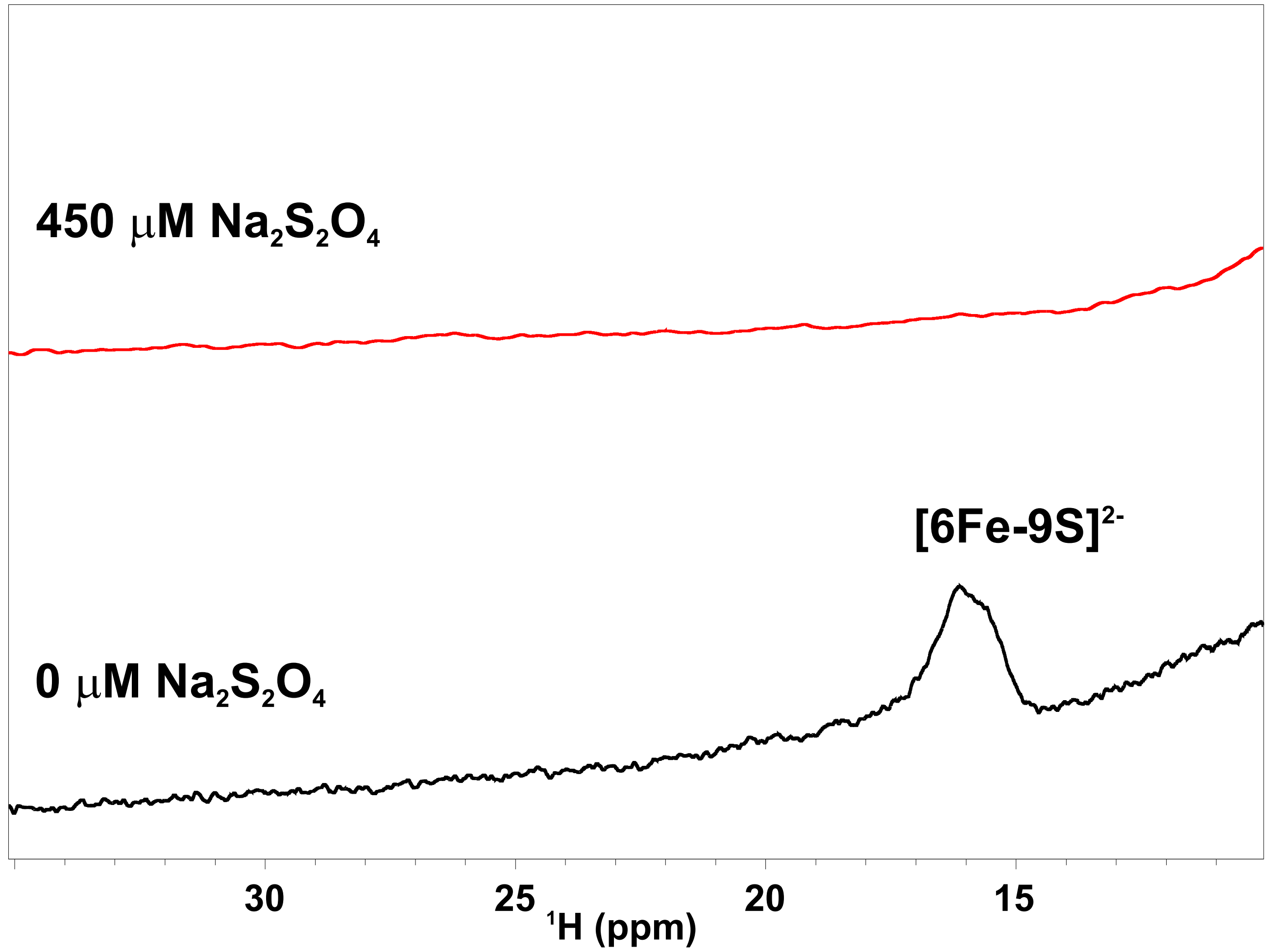

Supplementary Figure 35. Paramagnetic ^1H NMR spectra of 2:1 HS^-:Fe^{3+} glutathione (150 mM glutathione, 3.75 mM Na_2S, 1.87 mM $FeCl_3$) in D_2O, pD 8.6 before (black) and after (red) the addition of 450 μM sodium dithionite. Upon the addition of the reducing agent, the hyperfine resonance at 16.1 ppm, i.e. the Cys H_α of a $[6Fe\text{-}9S]^{2-}$ cluster, disappeared and no other paramagnetic resonances emerged. No signal of the $[Fe(SR)_4]^{2-}$ mononuclear species (e.g. resonance at 211 ppm) was observed.

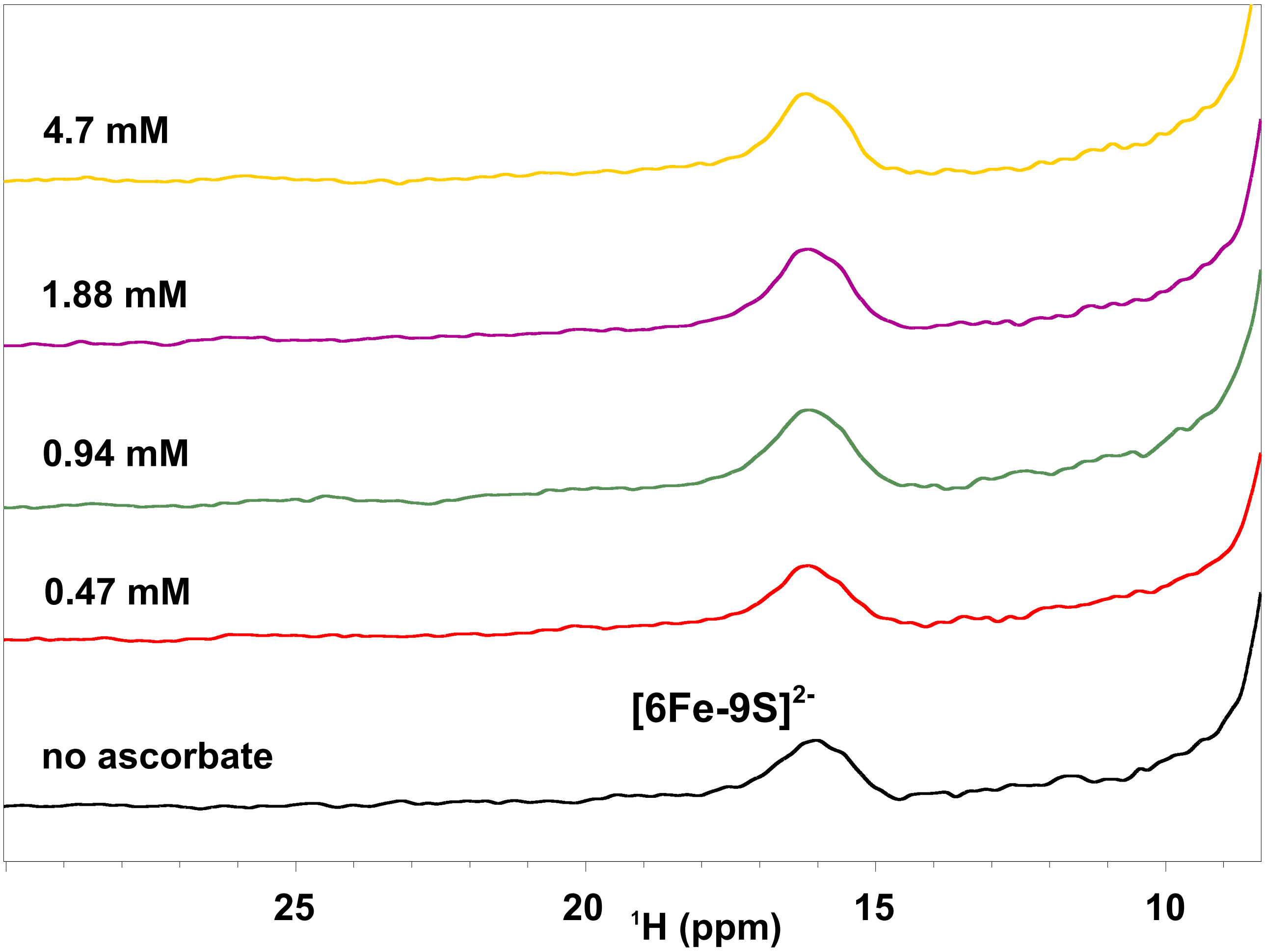

Supplementary Figure 36. Paramagnetic ^{1}H NMR spectra of 2:1 HS^{-}:Fe^{3+} glutathione (150 mM glutathione, 3.75 mM Na_2S, 1.87 mM $FeCl_3$) in D_2O, pD 8.6 titrated with the reducing agent sodium ascorbate. The concentration of sodium ascorbate was, from bottom to top, 0 mM (black), 0.47 mM (red), 0.94 mM (green), 1.88 mM (mauve), and 4.7 mM (yellow). No variation in the hyperfine resonance at 16 ppm (i.e. the Cys H_{α} of ligands to the putative $[6Fe\text{-}9S]^{2-}$ cluster) was detected.

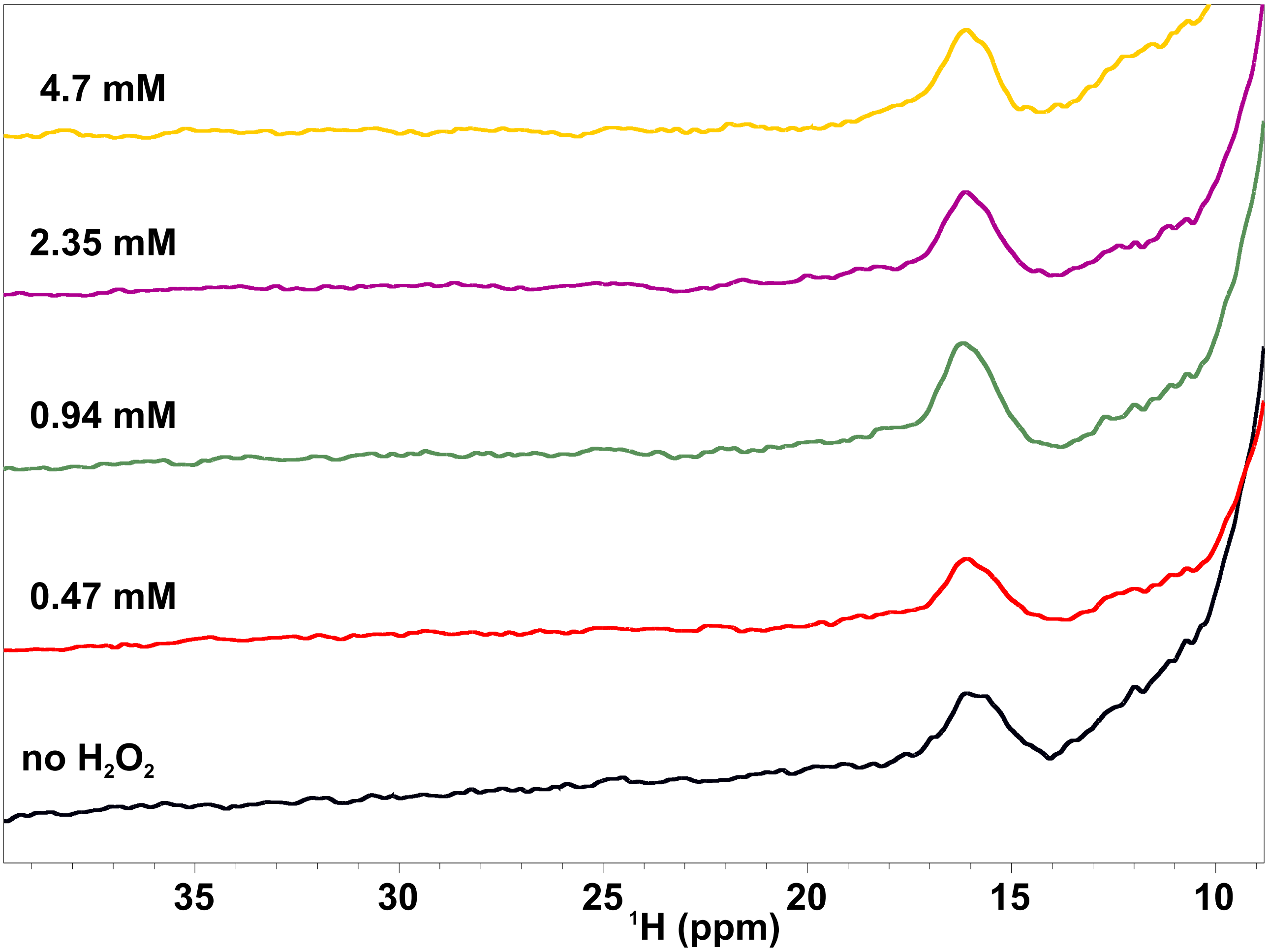

Supplementary Figure 37. Paramagnetic ^{1}H NMR spectra of 2:1 HS^{-}:Fe^{3+} glutathione (150 mM glutathione, 3.75 mM Na_2S, 1.87 mM $FeCl_3$) in D_2O, pD 8.6 titrated with hydrogen peroxide. From bottom to top, 0 (black), 0.47 mM (red), 0.94 mM (green), 2.35 mM (mauve), and 4.7 mM (yellow) hydrogen peroxide was added to the putative $[6Fe\text{-}9S]^{2-}$ cluster (0.3 mM). No variations in the hyperfine resonance at 16 ppm (i.e. the Cys H_{α} of ligands to the putative $[6Fe\text{-}9S]^{2-}$ cluster) were detected.

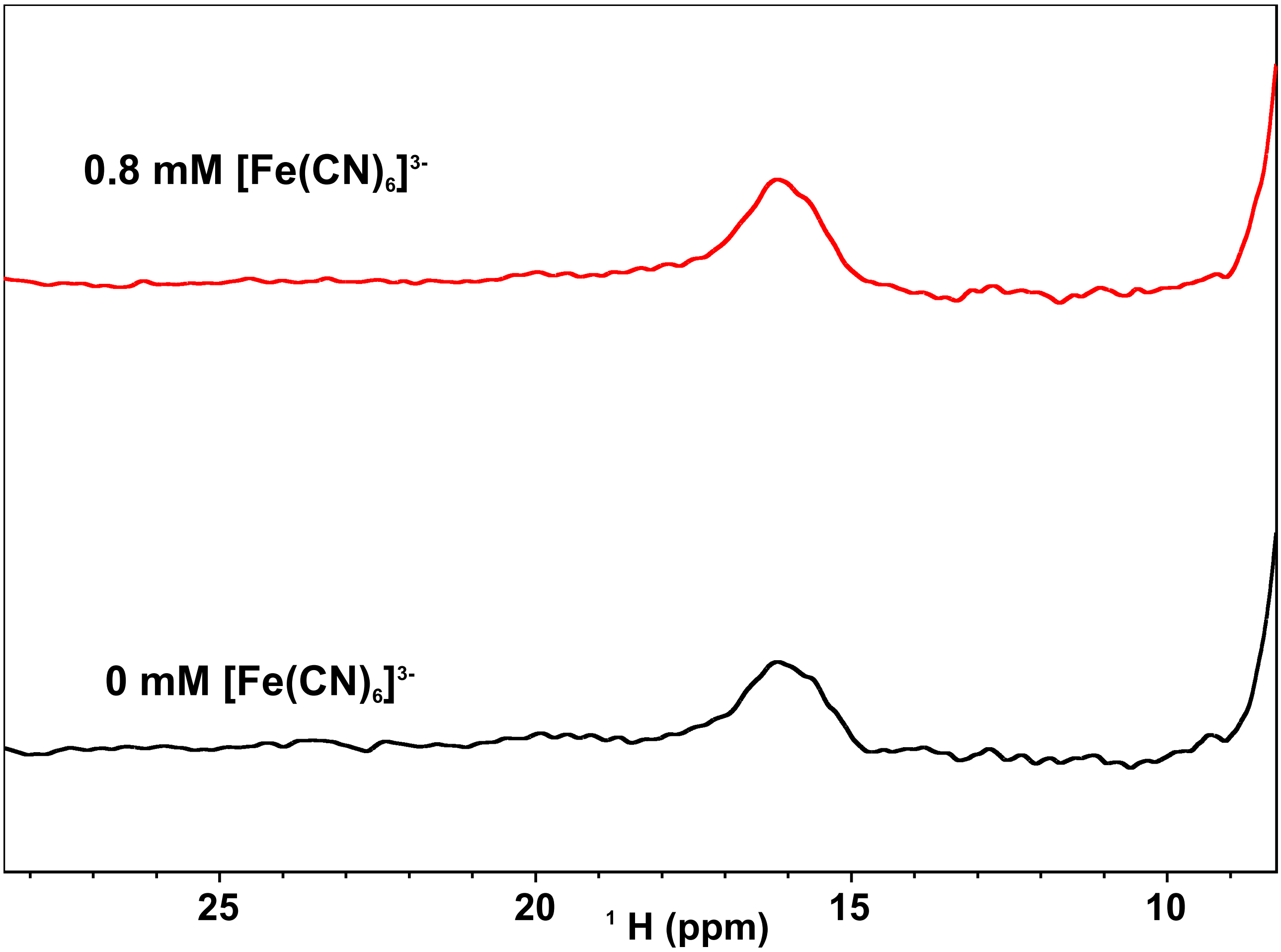

Supplementary Figure 38. The effect of potassium ferricyanide on paramagnetic ^{1}H NMR spectra of 2:1 HS^{-}:Fe^{3+} glutathione (150 mM glutathione, 3.75 mM Na_2S, 1.87 mM $FeCl_3$) in D_2O, pD 8.6. Spectra are of solutions containing 0 μM (black, bottom) and 0.80 mM potassium ferricyanide (red, top). The hyperfine resonance at 16 ppm, indicative of the putative $[6Fe\text{-}9S]^{2-}$ glutathione, was unperturbed by the addition of approximately two equivalents of potassium ferricyanide.

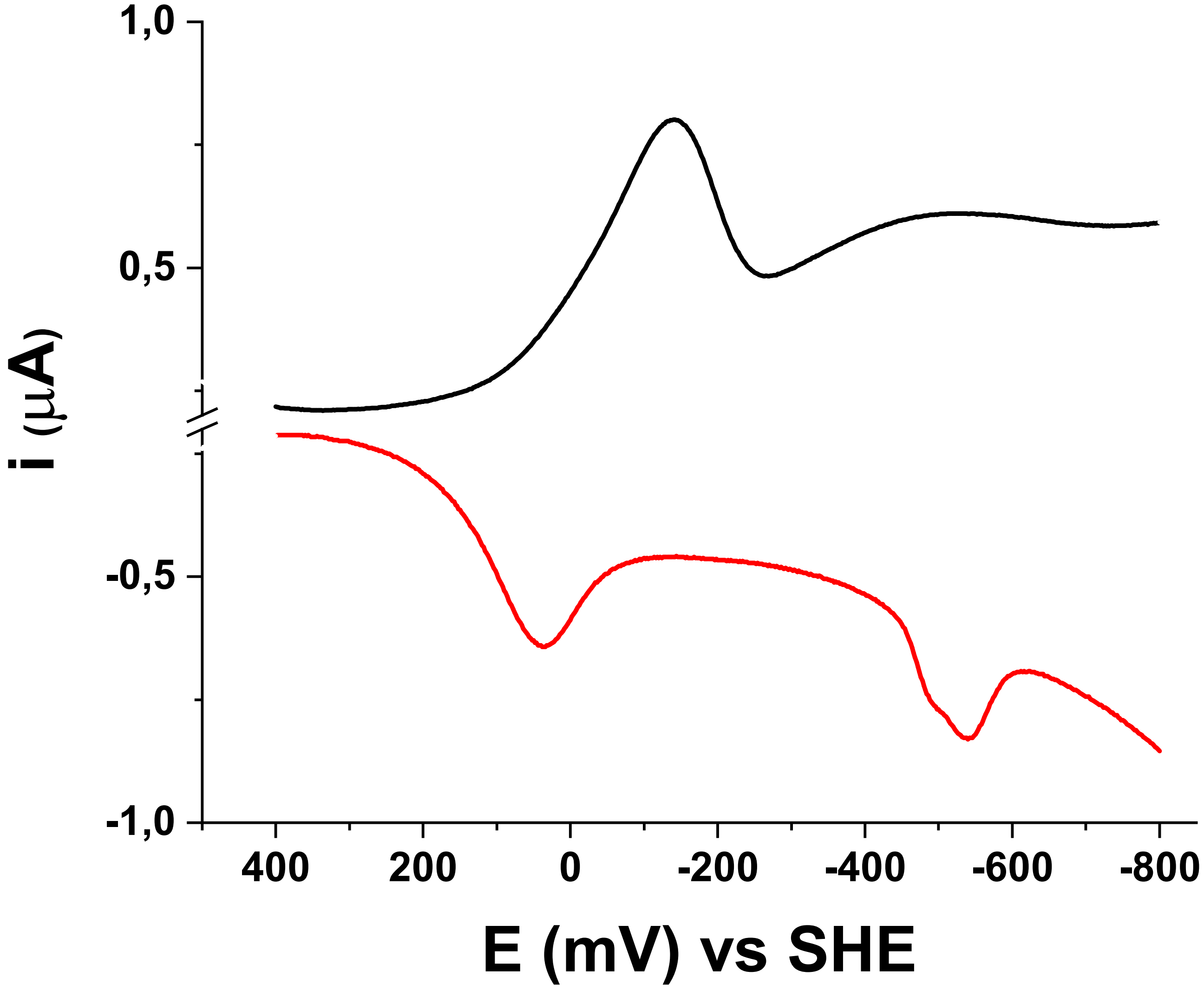

Supplementary Figure 39 Square wave voltammetries of 1.5:1 HS^{-}:Fe^{3+} glutathione (40 mM glutathione, 3 mM Na_2S, 2 mM $FeCl_3$) in H_2O, pH 8.6. Anodic square wave voltammetry (red curve) showed, together with the peak at -0.48 ± 0.01 V ([4Fe-4S]), a well defined current peak assigned to a putative [6Fe-9S] cluster found at -0.55 ± 0.01 V, which becomes prevalent for 2:1 HS^{-}:Fe^{3+} (see **Fig. 2**). Signals attributed to a mononuclear species can be observed at +0.04 ± 0.01 V (E_{ap}). Cathodic square wave voltammetry (black curve) showed a broad peak at -0.54 ± 0.01 V attributed to a putative [6Fe-9S] and overlapped to that of [4Fe-4S] (in **Fig. S27**, observed at -0.50 ± 0.01 V), which became prevalent for 2:1 HS^{-}:Fe^{3+} (see **Fig. 2**). Signals attributed to a mononuclear species can be observed at -0.13 ± 0.01 V (E_{cp}).

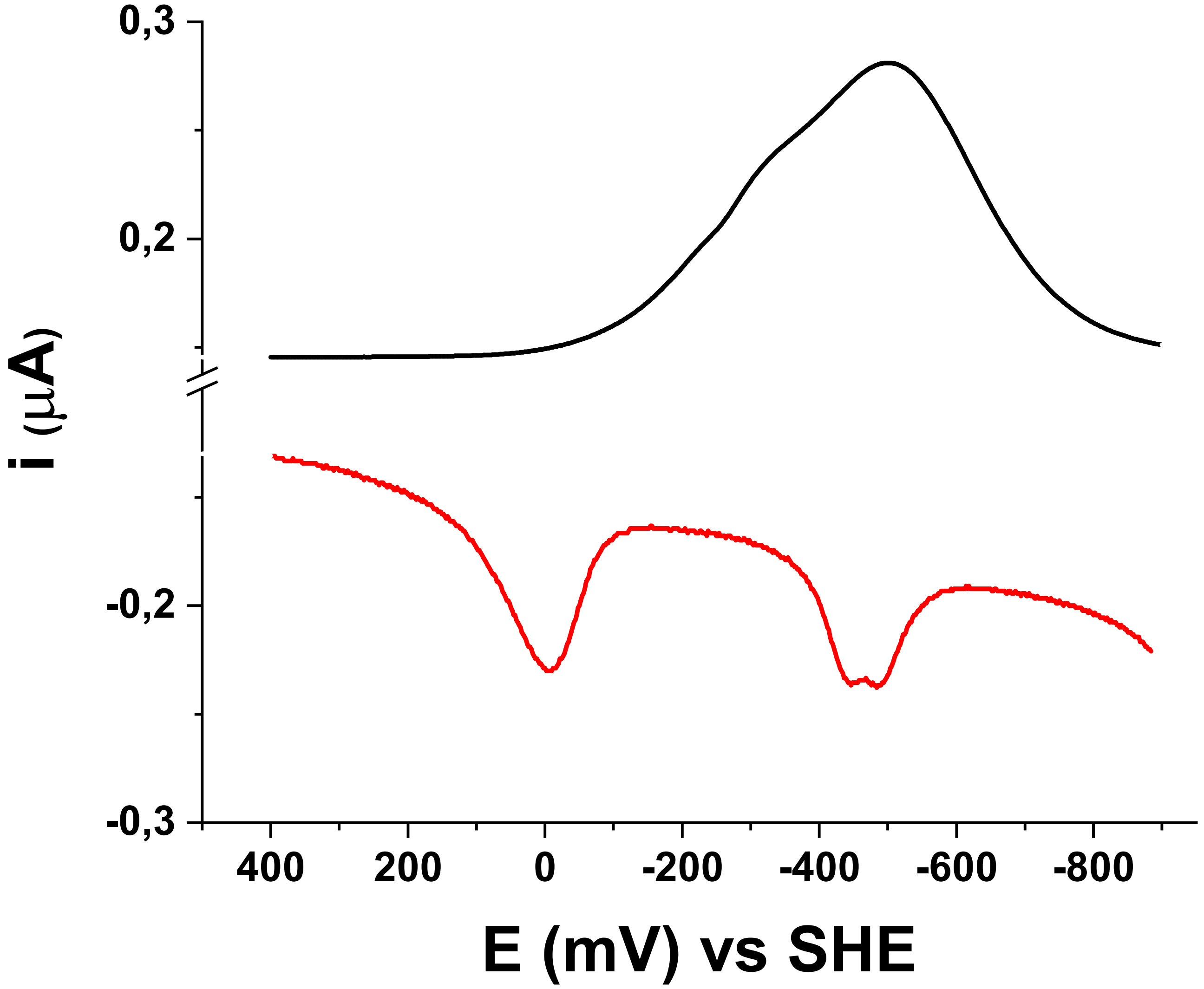

Supplementary Figure 40 SWV of 0.4:1:7.5 HS^{-}:Fe^{3+}:glutathione (0.8 mM Na_2S, 2 mM $FeCl_3$, 15 mM glutathione, 100 mM sodium carbonate pH 8.6). Cathodic scan (black curve) showed a composite signal consisting of a peak and two shoulders at E_{cp} = -0.50 ± 0.01 V ([4Fe-4S]), ca. -0.38 V (a new species) and ca. -0.21 V ([2Fe-2S]). In the anodic scan (red curve) peaks were found at -0.49 ± 0.01 V ([4Fe-4S]) and at -0.45 ± 0.01 V (new species). As already indicated, no anodic signal corresponding to [2Fe-2S] was observed, while an oxidation peak attributed to a rubredoxin-like mononuclear species (-0.01 ± 0.01 V) was found indicating reductive conversion of [2Fe-2S] into a rubredoxin-like species.

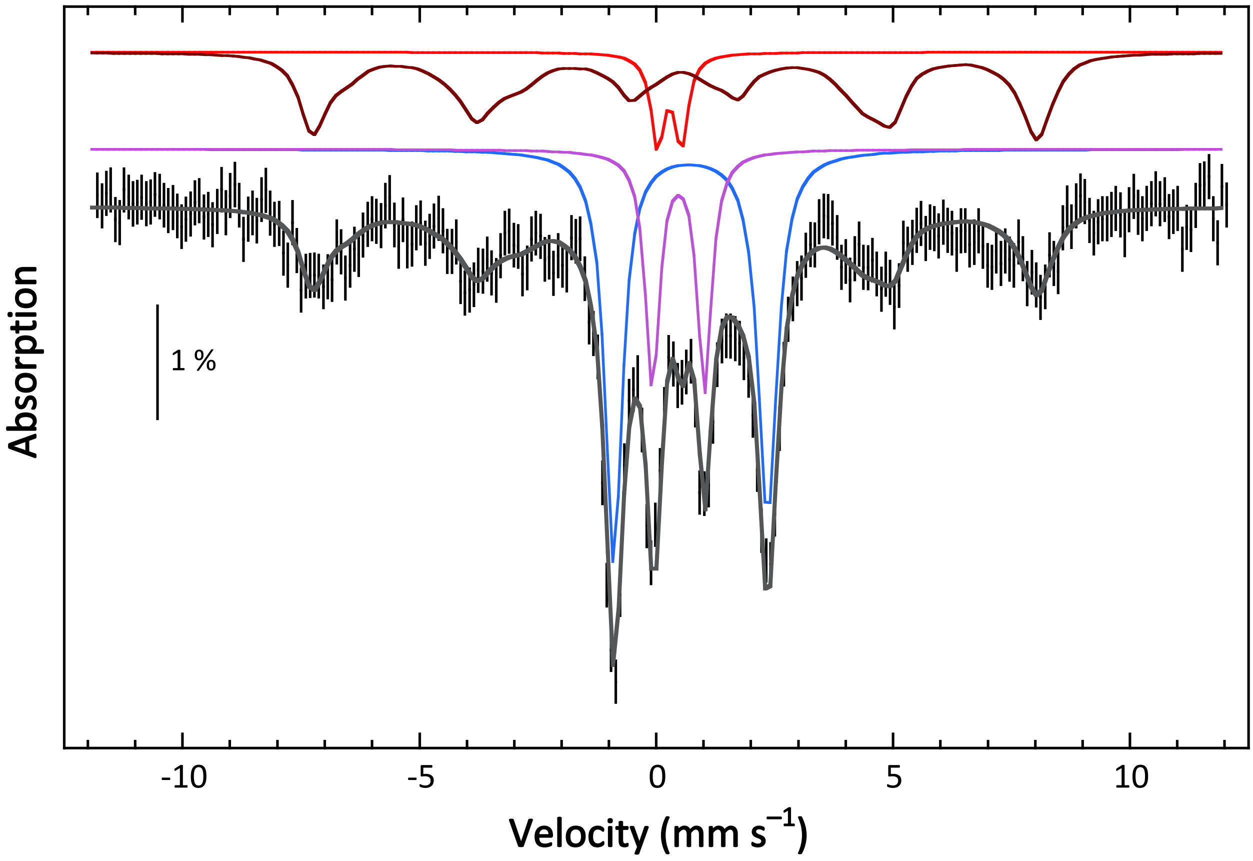

Supplementary Figure 41. Mössbauer spectra of the 0.4:1:7.5 HS^-:Fe^{3+}:glutathione (15 mM glutathione, 0.74 mM Na_2S and 2 mM $FeCl_3$) in H_2O with 100 mM sodium carbonate, pH 8.6 at 4.2 K with a 0.06 T external magnetic field applied parallel to the γ-rays. The overlaid simulation (thick grey solid line) is the sum of the four contributions displayed above the spectrum. The magnetic contribution can be satisfyingly reproduced by a *S*=5/2 species that presents parameters consistent with a hexa-coordinated ferric ion (see the values below). The central part of the spectrum presents five lines at –0.9, –0.1, 0.5, 1.0, and 2.3 mm s^{-1} that evidenced the mononuclear starting ferrous complex (lines 1 and 5), the diamagnetic [4Fe-4S] cluster (lines 2 and 4) and the diamagnetic [2Fe-2S cluster] (lines 2 and 3). No $[6Fe\text{-}9S]^{2-}$ was detected.

List of the parameters used to reproduce the spectrum:

Species	Color	δ (mm s^{-1})	ΔE_Q (mm s^{-1})	Contribution (%)
**$[1Fe\text{-}0S]^{2+}$**	blue	0.73	3.25	36
**$[2Fe\text{-}2S]^{2+}$**	red	0.28	0.50	6
**$[4Fe\text{-}4S]^{2+}$**	mauve	0.47	1.10	17
**High-spin Fe^{III} (*S*=5/2)**	brown	0.49	0.92	39

The other parameters used for the *S*=5/2 are:
D = 0.88 cm^{-1}; E/D = –0.29; g_{iso} = 2.0; $a_{x,y,z}/(g_n\mu_n)$ = –20.0/–29.6/–19.0 T; η = 0.53

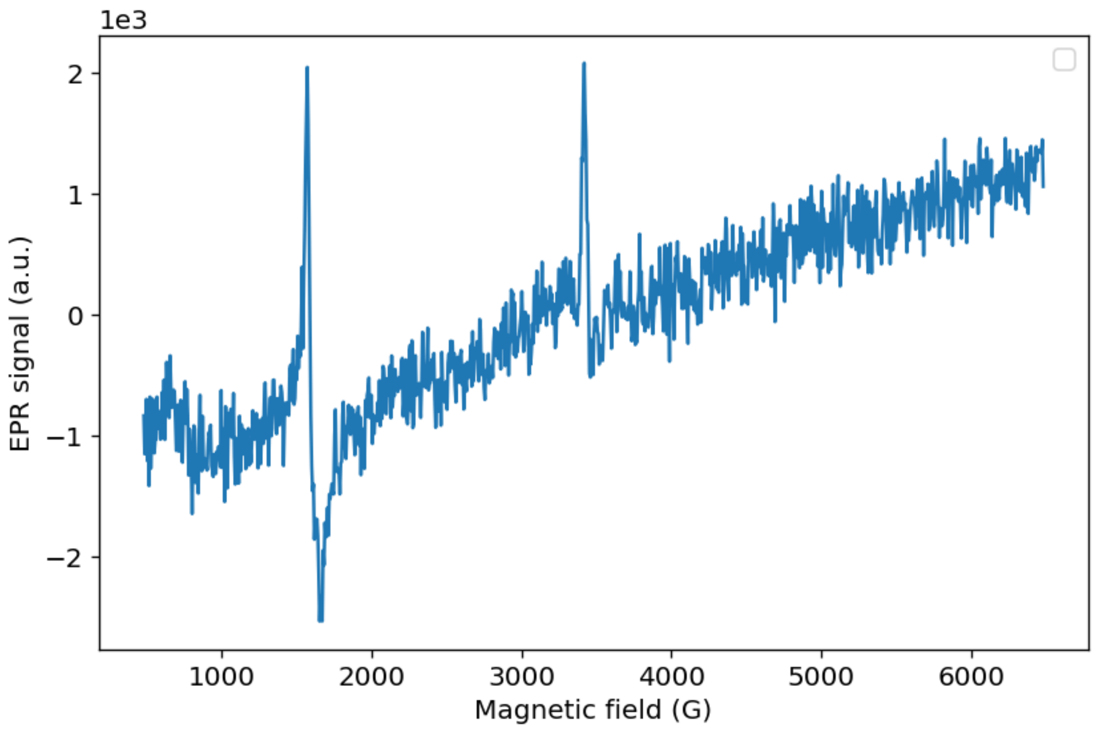

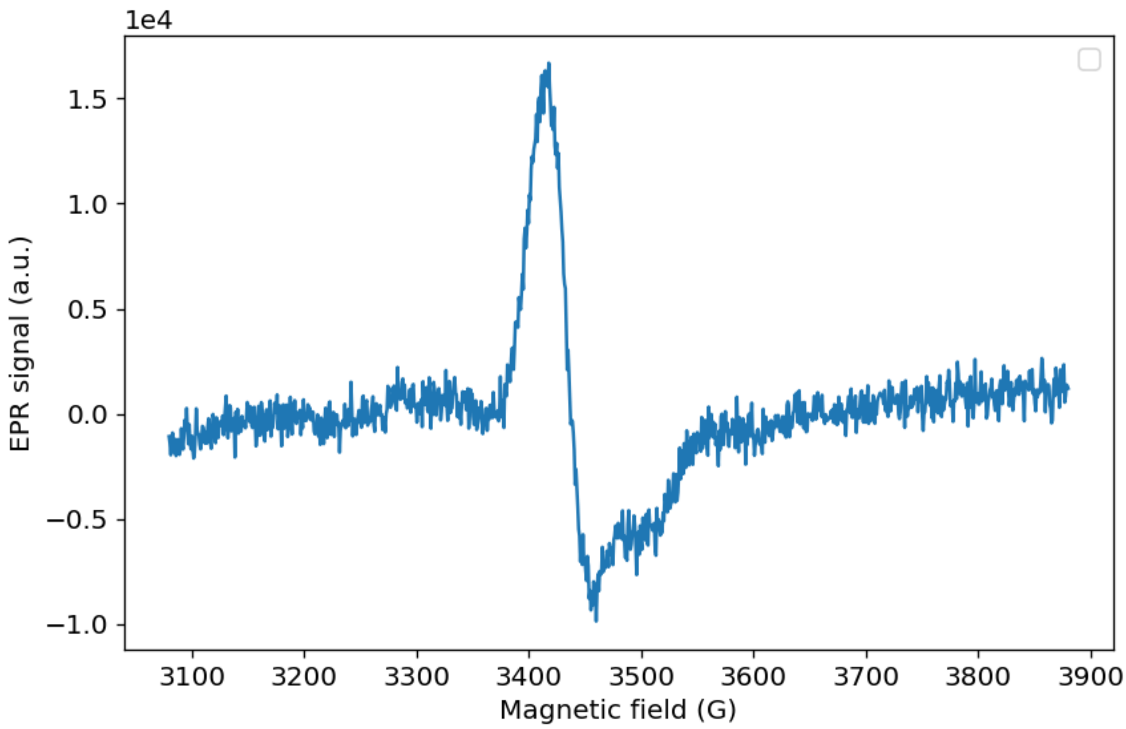

Supplementary Figure 42. EPR spectra of the 0.4:1:7.5 HS^{-}:Fe^{3+}:glutathione (15 mM glutathione, 0.74 mM Na_2S, 2 mM $FeCl_3$) in H_2O, and 100 mM carbonate, pH 8.6 at 6K immediately after mixing (large window, top) and at 20 K (window centered around g=2, bottom). In the top spectrum, two species can be observed. One (around g= 4.4) corresponds to a Fe^{3+} complex in octahedral symmetry (S=5/2), the other corresponds to a S=1/2 species ($[4Fe\text{-}4S]^{+}$, g=2). In the bottom image, the spectrum is dominated by the signal attributed to $[4Fe4S]^{+}$.

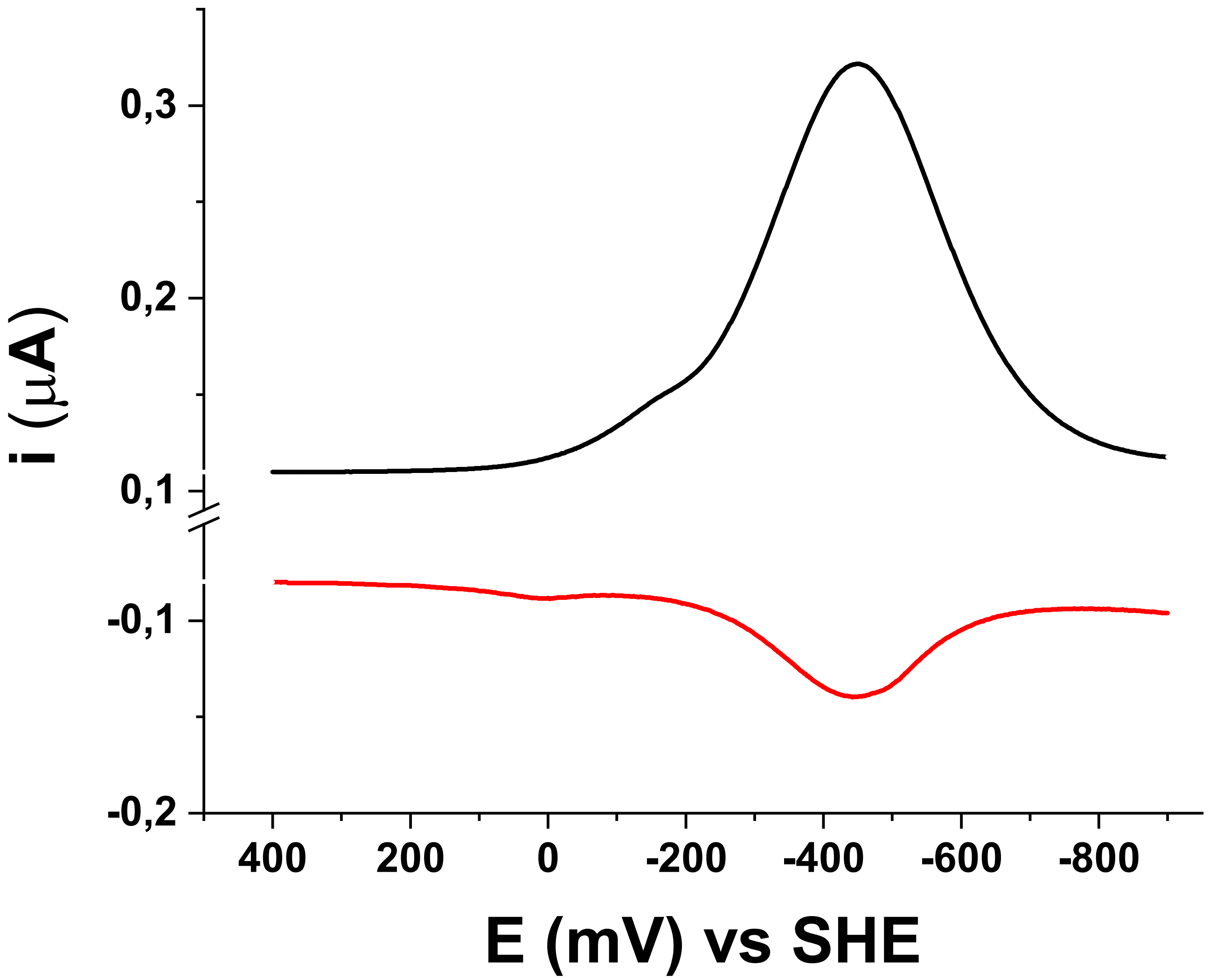

Supplementary Fig. 43. SWV of 1:1:7.5 HS^{-}:Fe^{3+}:glutathione (2 mM Na_2S, 2 mM $FeCl_3$, 15 mM glutathione, 100 mM sodium carbonate, pH 8.6). Cathodic scan (black curve) showed the main presence of a signal at E_{cp} = -0.46 ± 0.01 V (new species, tentatively a [4Fe-4S]), with a corresponding anodic peak (red curve) at -0.45± 0.01 V. Similarly to lower HS^{-}:Fe^{3+} ratios, traces of cathodic and anodic signals related to mononuclear complex (E°' = -0.01 ± 0.01 V) can be observed.

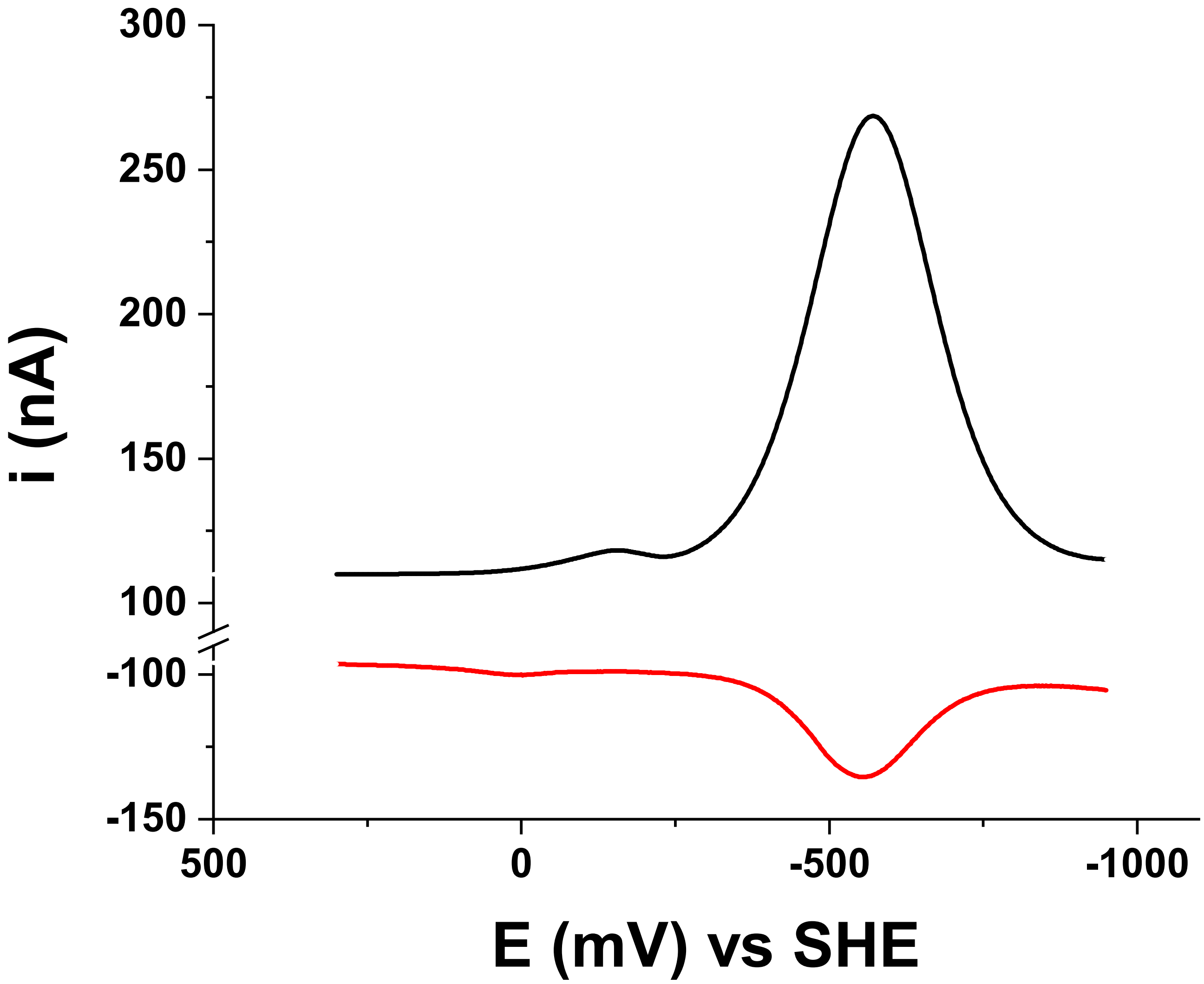

Supplementary Fig. 44. SWV of 2:1:7.5 HS^-:Fe^{3+}:glutathione (4 mM Na_2S, 2 mM $FeCl_3$, 15 mM glutathione, 100 mM sodium carbonate, pH 8.6). As the concentration of HS^- was further increased above 1:1 HS^-:Fe^{3+}, the signal at the more positive potential (new species, with potentials compatible with a [4Fe-4S]) decreased. Under these conditions, another signal formed at -0.57 ± 0.01 V in the cathodic scan (black curve) and at -0.56 ± 0.01 V in the anodic scan (red curve), respectively, that become prevalent at HS^-/Fe^{3+} molar ratio ≥ 2. This signal could be related to the formation of a putative $[6Fe\text{-}9S]^{2-}$ cluster. Furthermore, the presence of a residual mononuclear species (0.01 V vs SHE) confirmed the degradation of the $[2Fe\text{-}2S]^{2+}$ cluster.

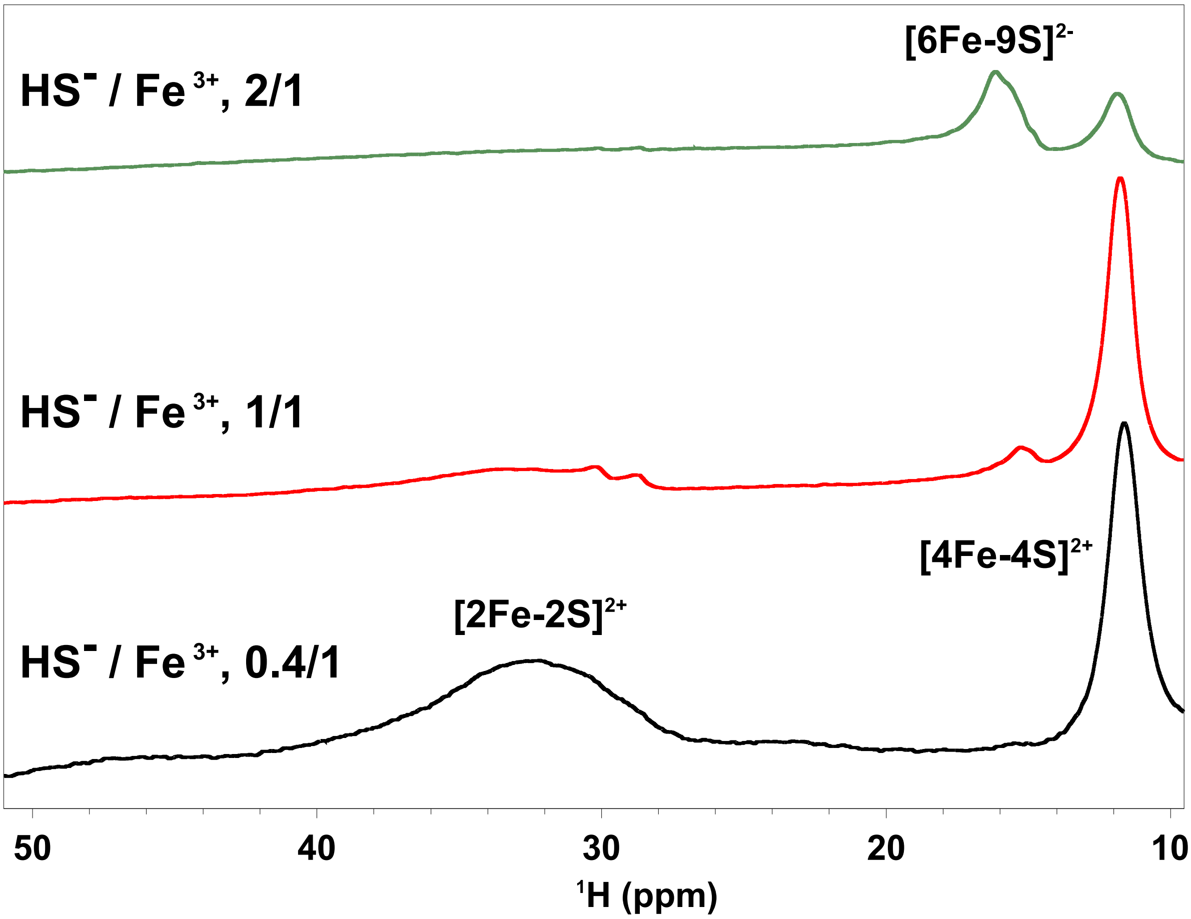

Supplementary Figure 45. ^{1}H NMR paramagnetic spectra of 15 mM of glutathione, 2 mM $FeCl_3$, and 100 mM sodium carbonate, pH 8.6 with the addition of 0.74 mM (black, bottom, 0.4:1 HS^{-}:Fe^{3+}), 2 mM (red, middle, 1:1 HS^{-}:Fe^{3+}), and 4 mM Na_2S (green, top, 2:1 HS^{-}:Fe^{3+}). At 0.4:1 HS^{-}:Fe^{3+}, resonances at 11.8 ($[4Fe\text{-}4S]^{2+}$) and 33 ppm ($[2Fe\text{-}2S]^{2+}$) were detected, while no resonance of the mononuclear, rubredoxin-like species was observed (see **Fig. S46**). Increasing concentration of hydrosulfide progressively reduced the contribution of the resonance at 11.8 ppm ($[4Fe\text{-}4S]^{2+}$) and increased the resonance at 15 ppm (intermediate structure between [4Fe-4S] and [6Fe-9S] clusters). At 2:1 HS^{-}:Fe^{3+}, the putative $[6Fe\text{-}9S]^{2-}$ cluster was the main cluster type detected in solution together with residual $[4Fe\text{-}4S]^{2+}$.

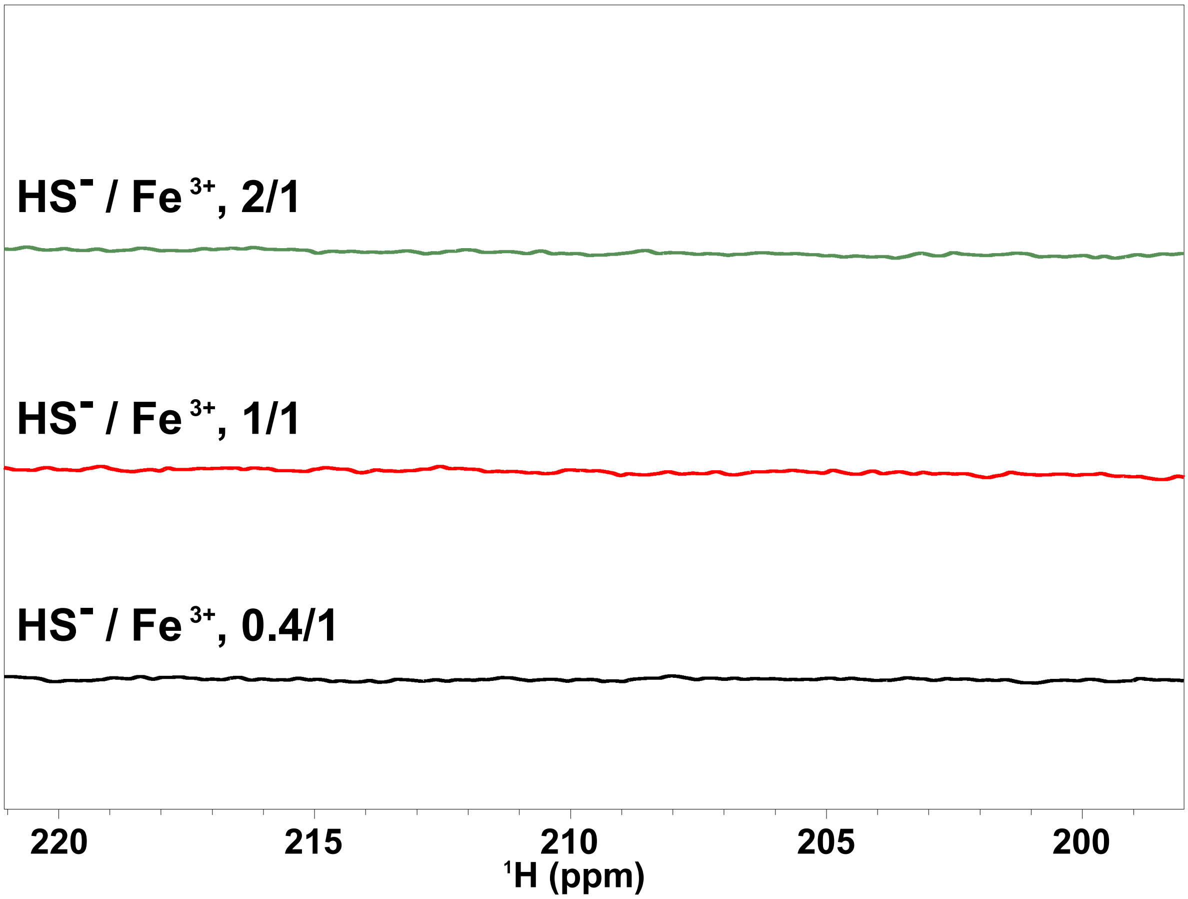

Supplementary Figure 46. ^{1}H NMR paramagnetic spectra of 15 mM of glutathione, 2 mM $FeCl_3$, and 100 mM sodium carbonate, pH 8.6 with the addition of 0.74 mM (black, bottom, 0.4:1 HS^-:Fe^{3+}), 2 mM (red, middle, 1:1 HS^-:Fe^{3+}), and 4 mM Na_2S (green, top, 2:1 HS^-:Fe^{3+}). The rubredoxin-like $[Fe(SR)_4]^{2-}$ mononuclear species was not detected (resonance at 211 ppm) at any of the HS^-:Fe^{3+} tested. The related upfield region is reported in **Fig. S45**.

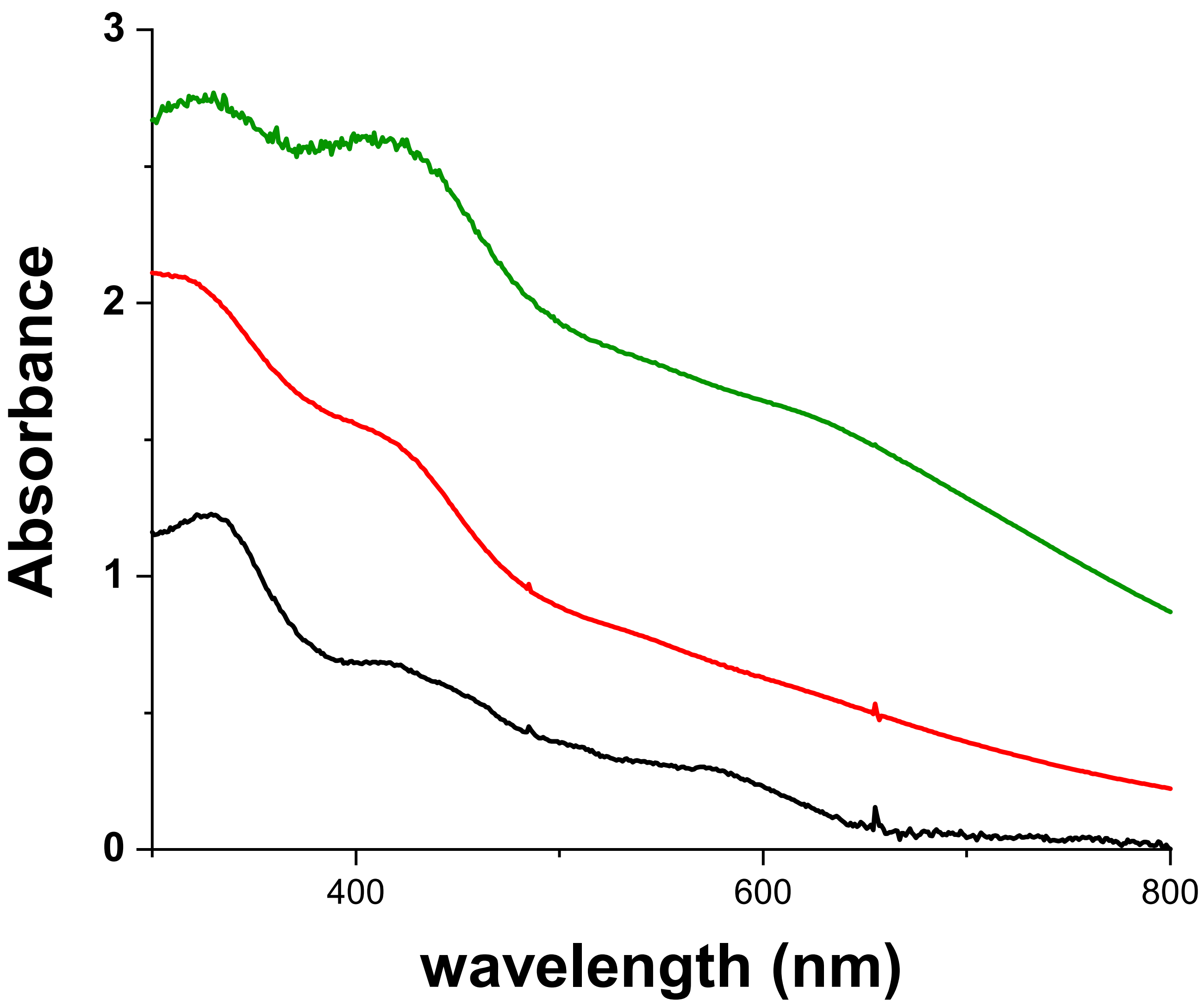

Supplementary Figure 47. UV-vis absorption spectra of 2.5 mM of glutathione, 0.3 mM $FeCl_3$, and 40 mM sodium carbonate, pH 8.6 with the addition of 0.1 mM (black, bottom, 0.4:1 HS^-:Fe^{3+}), 0.3 mM (red, middle, 1:1 HS^-:Fe^{3+}), and 0.6 mM Na_2S (green, bottom, 2:1 HS^-:Fe^{3+}), respectively. In the presence of carbonate, for S/Fe ratio 0.4 (black curve) the UV-vis spectrum suggested a mixture $[2Fe\text{-}2S]^{2+}$/ $[4Fe\text{-}4S]^{2+}$ (330, 420 and 578 nm).[11] With the progressive increase of the S/Fe ratio, the spectrum assumed mainly traits of $[4Fe\text{-}4S]^{2+}$ and the putative $[6Fe\text{-}9S]^{2-}$ (broad 320 and 410 nm, S/Fe 1, red curve) and, for higher S/Fe ratios, typical features of $[6Fe\text{-}9S]^{2-}$ (330, 416 and 578 nm, green curve).[11]

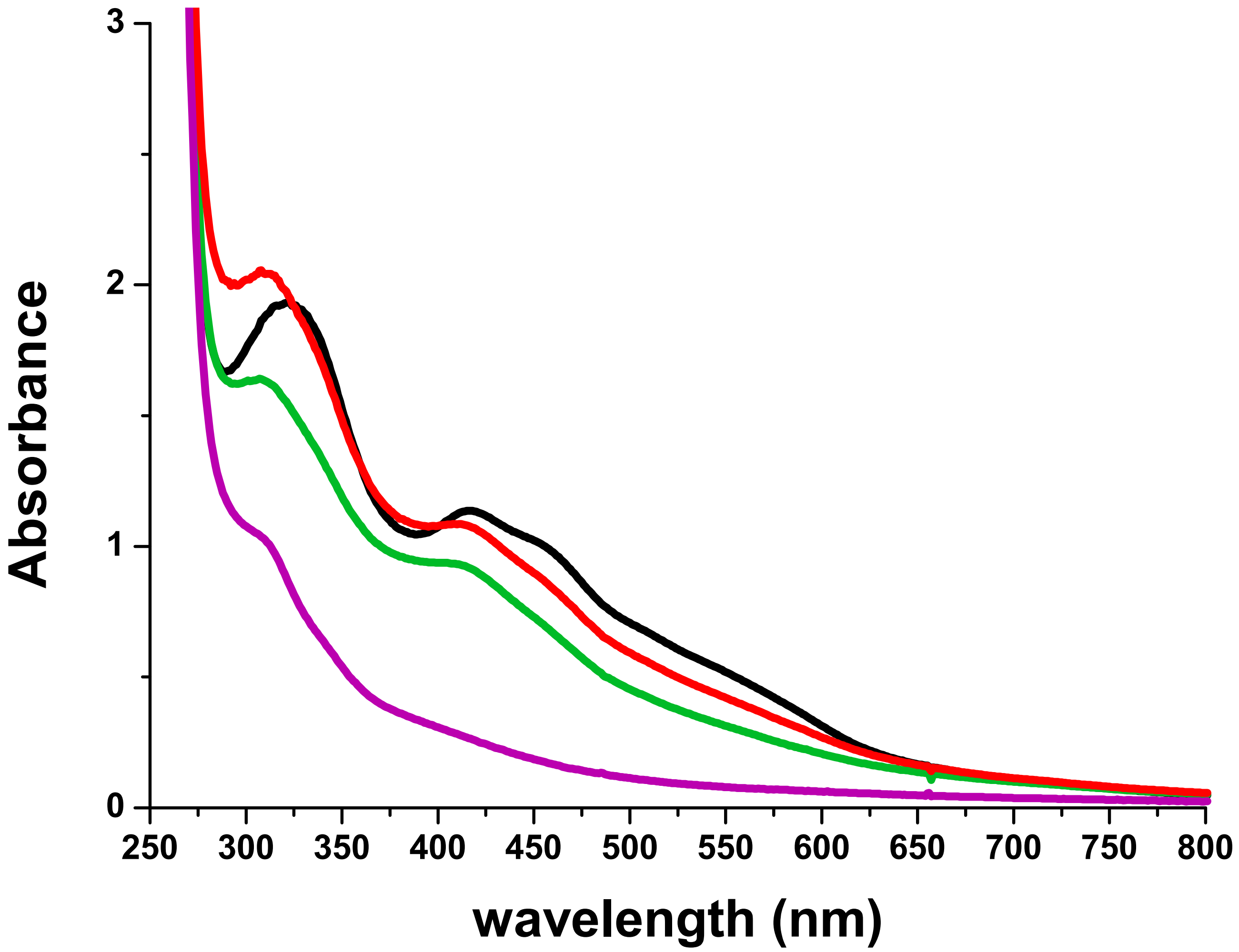

Supplementary Figure 48. UV-visible absorption spectra of 40 mM glutathione, 180 μM Na_2S to which 500 μM iron ions were added. Although the total concentration of iron ions was kept constant, the percent of Fe^{2+} and Fe^{3+} was varied. The conditions were, from top to bottom, 100% Fe^{3+} (black), 70% Fe^{3+} (red), 50% Fe^{3+} (green), 0% Fe^{3+} (mauve). When only Fe^{3+} and no Fe^{2+} was provided, the absorption bands at 420 nm and 450 nm that are typically observed for a $[2Fe\text{-}2S]^{2+}$ cluster were detected (black curve). By progressively decreasing the fraction of Fe^{3+} to 70% and 50%, the absorption bands indicative of the $[2Fe\text{-}2S]^{2+}$ cluster decreased. The spectra were indicative of a mixture of $[2Fe\text{-}2S]^{2+}$ and $[4Fe\text{-}4S]^{2+}$ clusters (red and green curve, respectively). When no Fe^{3+} was added (i.e. only Fe^{2+} was present), no absorption band consistent with a polynuclear iron-sulfur cluster was observed (mauve curve).

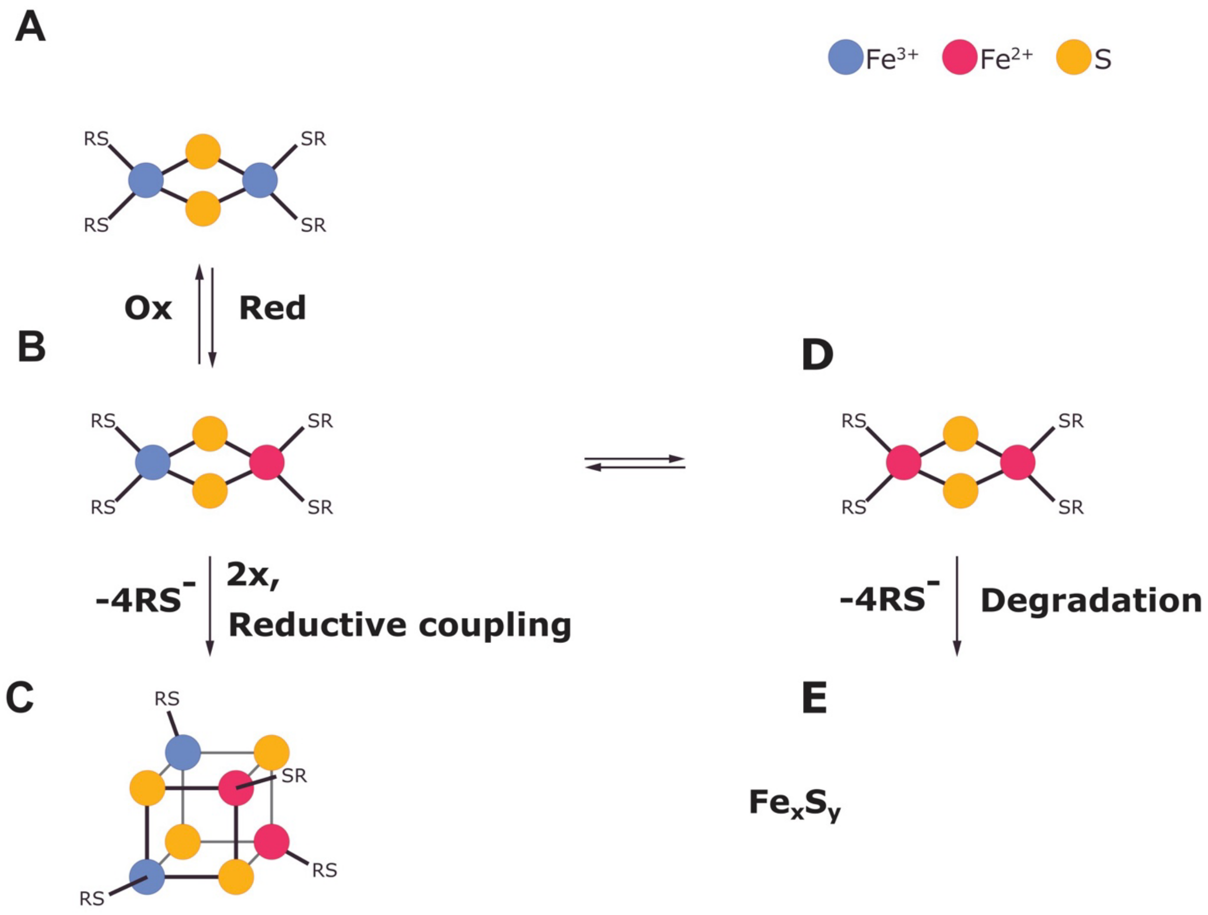

Supplementary Figure 49. The reductive coupling pathway for the synthesis of [4Fe-4S] clusters. RS indicates a generic thiol-containing ligand. Red circles correspond to ferrous ions, blue circles to ferric ions. Yellow circles correspond to sulfide ions. The A to C pathway illustrates the classic hypothesized condensation of two $[2Fe\text{-}2S]^{+}$ units (B) into a $[4Fe\text{-}4S]^{2+}$. A fully reduced [2Fe-2S] (D) would result in the degradation of the cluster core into iron sulfides and glutathione (B to E).

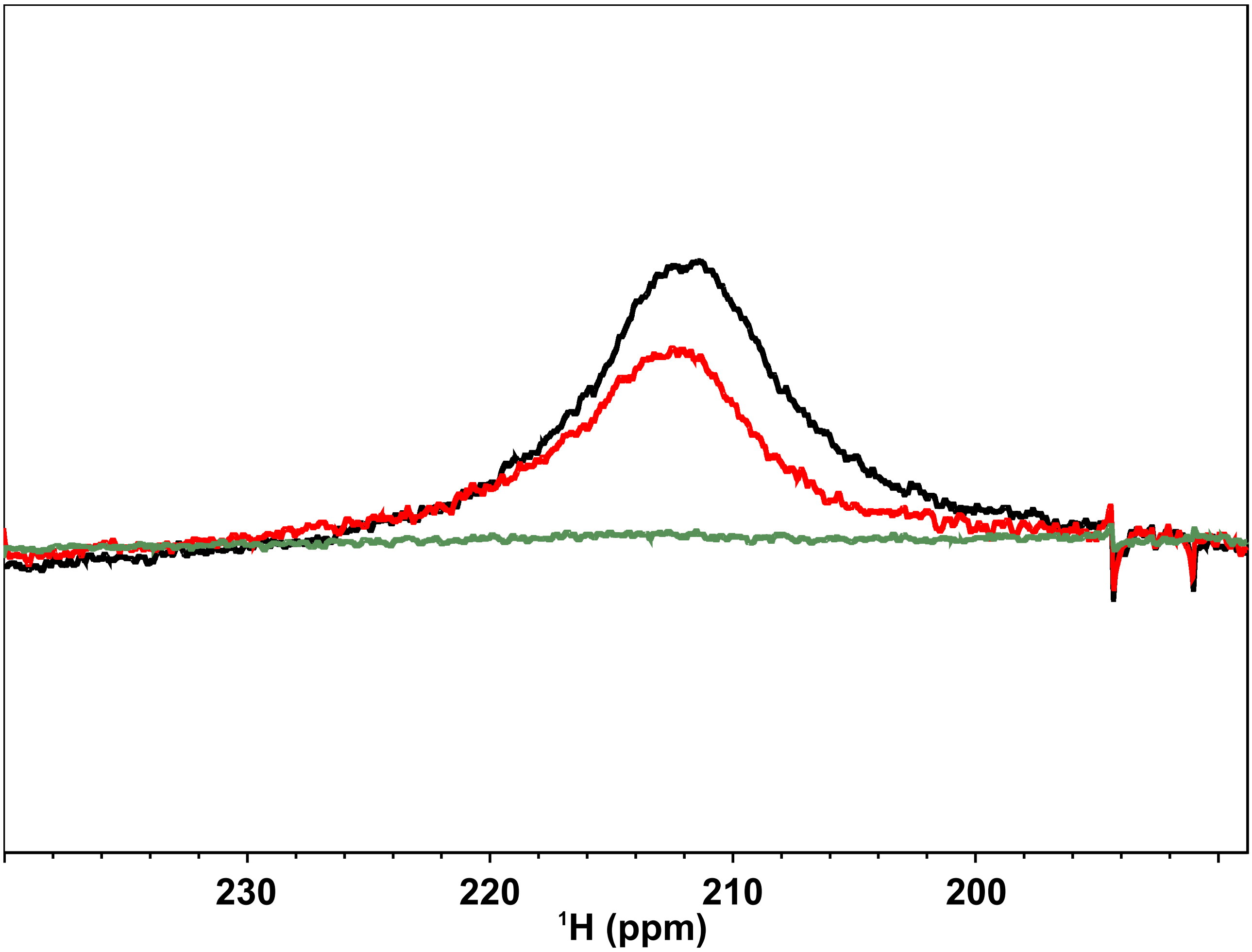

Supplementary Fig. 50. Paramagnetic ^1H NMR spectra of 0.4:1 HS^-:Fe^{3+} glutathione (150 mM glutathione, 2.77 mM Na_2S, 7.5 mM $FeCl_3$) in D_2O, pD 8.6 before (black) and after the addition of 2.4 mM (red) and 4.8 mM (green) Na_4EDTA to the mixture. The concentration of EDTA was approximately equivalent to the concentration of mononuclear species. Before the addition of EDTA, the mixture contained the reduced, rubredoxin-like, mononuclear species (211 ppm), $[2Fe\text{-}2S]^{2+}$ cluster (33 ppm) and traces of $[4Fe\text{-}4S]^{2+}$ at 11.8 ppm (see **Fig. S51**). Upon the addition of EDTA, the intensity of the resonance at 211 ppm (mononuclear species) reduced, consistent with the removal of the Cys of glutathione from the Fe^{2+} coordination sphere.

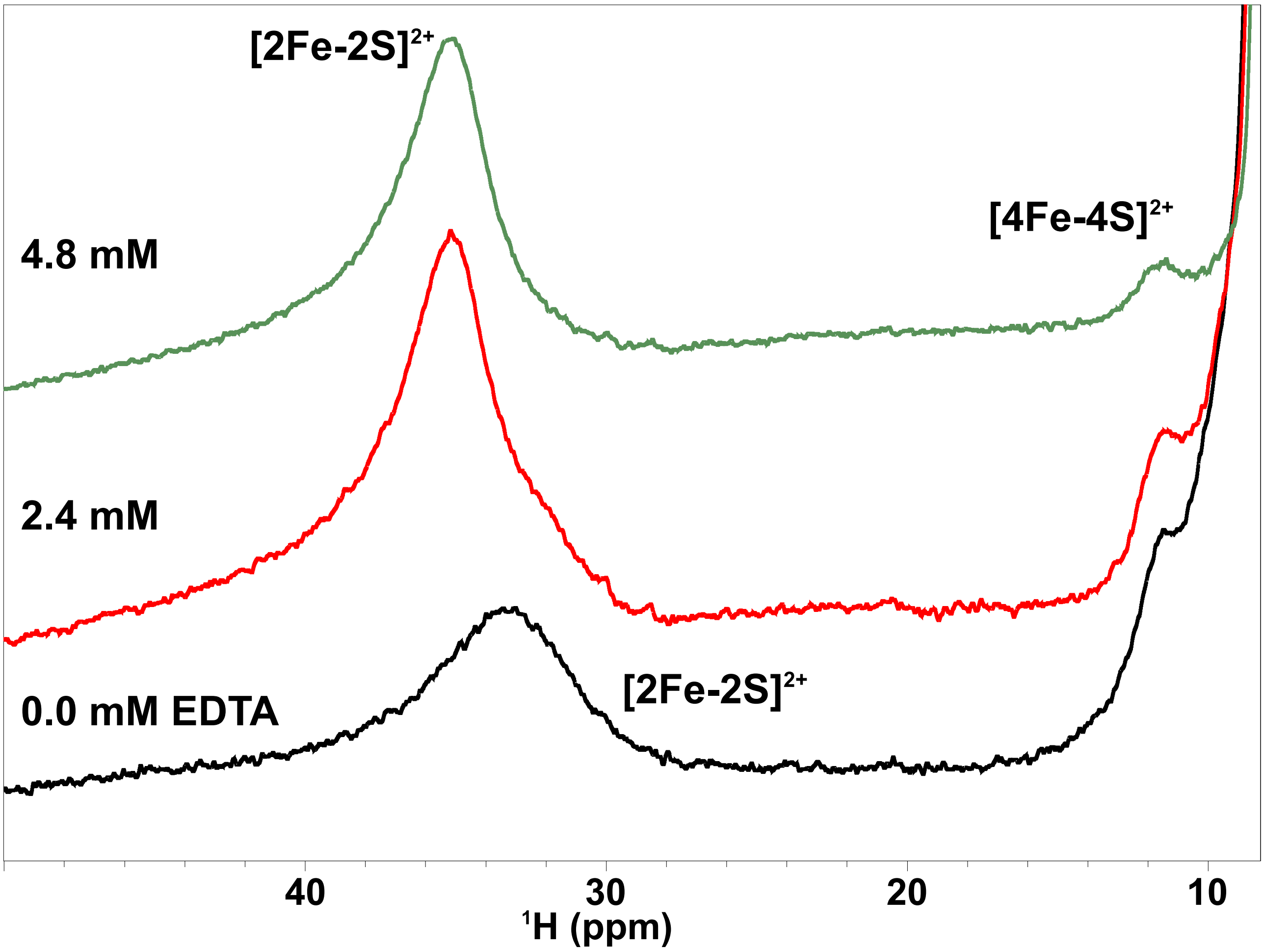

Supplementary Figure 51. Paramagnetic ^{1}H NMR spectra of 0.4:1 HS^-:Fe^{3+} glutathione (150 mM glutathione, 2.77 mM Na_2S, 7.5 mM $FeCl_3$) in D_2O, pD 8.6 before (black, bottom) and after the addition of 2.4 mM (red, middle) and 4.8 mM (green, top) Na_4EDTA to the mixture. Before the addition of EDTA, the solution contained $[2Fe\text{-}2S]^{2+}$ cluster (33 ppm), traces of $[4Fe\text{-}4S]^{2+}$ (11.8 ppm) and, downfield, reduced rubredoxin-like mononuclear species (211 ppm, **Fig. S50**). Upon the addition of 2.4 mM Na_4EDTA, the resonance corresponding to the reduced, mononuclear species diminished and the resonance corresponding to the $[2Fe\text{-}2S]^{2+}$ cluster (33 pm) resulted overlapped to a new, sharp resonance at 35 ppm (red curve). Upon addition of 4.8 mM Na_4EDTA to the mixture, the resonance related to the new species increased. At the same time, the resonance at 33 ppm ($[2Fe\text{-}2S]^{2+}$) is no longer detected, and only traces of $[4Fe\text{-}4S]^{2+}$ resonance at 11.8 ppm are found. The new resonance detected can be attributable to a [2Fe-2S] system more stabilized in the absence of mononuclear complex. In fact, native $[2Fe\text{-}2S]^{2+}$ ferredoxins have been reported to show Cys_β resonance in the region 32-37 ppm[13–15], as well as model complexes[11] and in particular peptide complexes in DMSO exhibit similar shape and chemical shift. This result strongly put in evidence the existence of a very sensitive equilibrium between the clusters in solution. By considering the fast ligand exchange experienced at HS^-:Fe^{3+} < 1, it is likely that the removal of the mononuclear species by means of EDTA contributed in stabilizing the coordination sphere of the dinuclear complex, thus lowering the ligand exchange rate and, subsequently, the reductive coupling route towards $[4Fe\text{-}4S]^{2+}$.

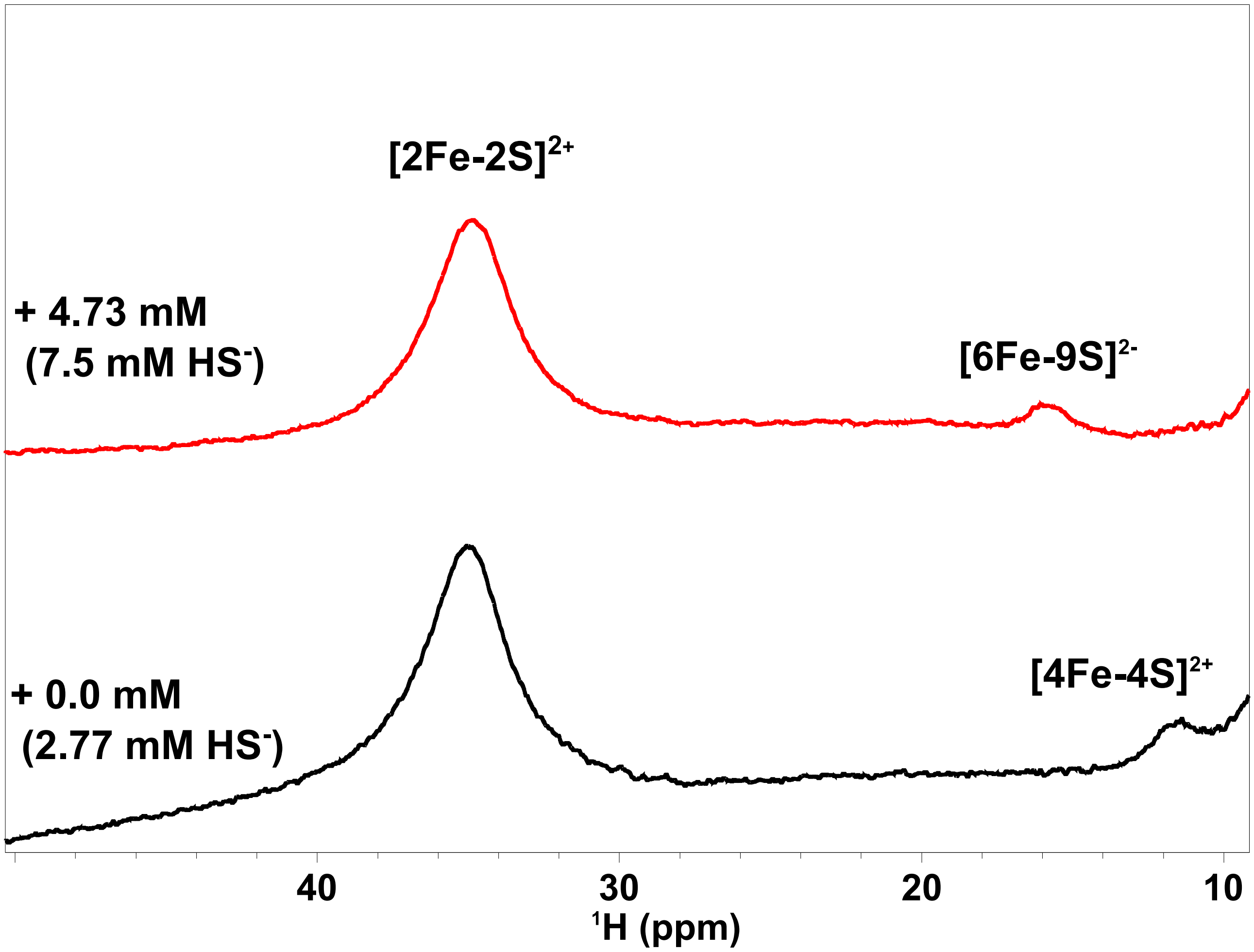

Supplementary Figure 52. Paramagnetic ^{1}H NMR spectra of 0.4:1 HS^{-}:Fe^{3+} glutathione (150 mM glutathione, 2.77 mM Na_2S, 7.5 mM $FeCl_3$, 4.8 mM Na_4EDTA) in D_2O, pD 8.6 before (black, bottom) and after the addition of 4.73 mM (red) Na_2S to the mixture (final [HS^{-}] = 7.5 mM). Before the addition of HS^{-}, the solution contained a species characterized by a resonance at 35 ppm and traces of $[4Fe\text{-}4S]^{2+}$. Upon the addition of Na_2S to the mixture, the resonance corresponding to the $[4Fe\text{-}4S]^{2+}$ cluster (11.8 ppm) disappeared and the resonance corresponding to the putative $[6Fe\text{-}9S]^{2+}$ cluster (16 ppm) emerged, with the resonance at 35 ppm (tentatively attributed to a $[2Fe\text{-}2S]^{2+}$) which resulted unchanged. This experiment indicates that the species characterized by the 35 ppm resonance is not involved in the conversion of the $[4Fe\text{-}4S]^{2+}$ cluster to the putative $[6Fe\text{-}9S]^{2-}$ species when no $[1Fe\text{-}0S]^{2+}$ is present. xx

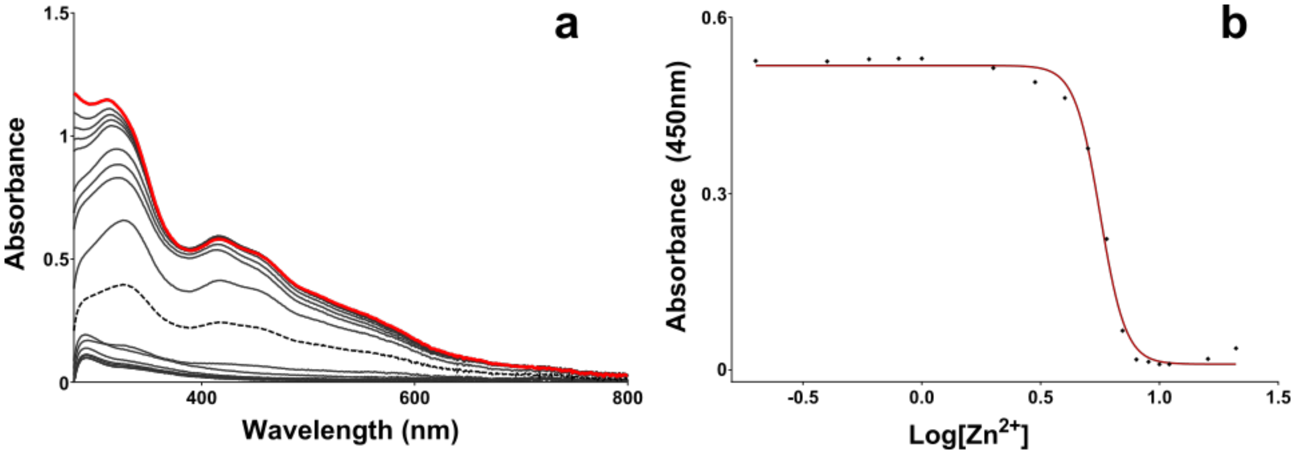

Supplementary Fig. 53. Calculation of concentration of zinc sulfate ($ZnSO_4$) needed to degrade half of the oxidized $[2Fe\text{-}2S]^{2+}$ cluster concentration ($[Zn]_{50}$) in the mixture 0.4:1 HS^-:Fe^{3+} glutathione (40 mM glutathione, 0.2 mM Na_2S, 0.5 mM $FeCl_3$) in H_2O, pH 8.6. The Zn salt was progressively added to the solution and related UV spectra collected at each addition. a) UV-visible absorption spectra of the Zn^{2+} titration of a solution containing $[2Fe\text{-}2S]^{2+}$ glutathione (red, solid curve, bands at 420 and 450 nm) and rubredoxin-like reduced mononuclear complex (no distinctive absorption bands). With the increase of the concentration of Zn^{2+}, the intensity of the spectra progressively decreased, without any significant change in the position of the absorption bands. The black dotted curve corresponded to $[Zn^{2+}]_{50}$ added to the solution. b) Fitting of absorbance at 450 nm as a function of Zn^{2+} concentration; $[Zn^{2+}]_{50}$ = 5.6 mM.

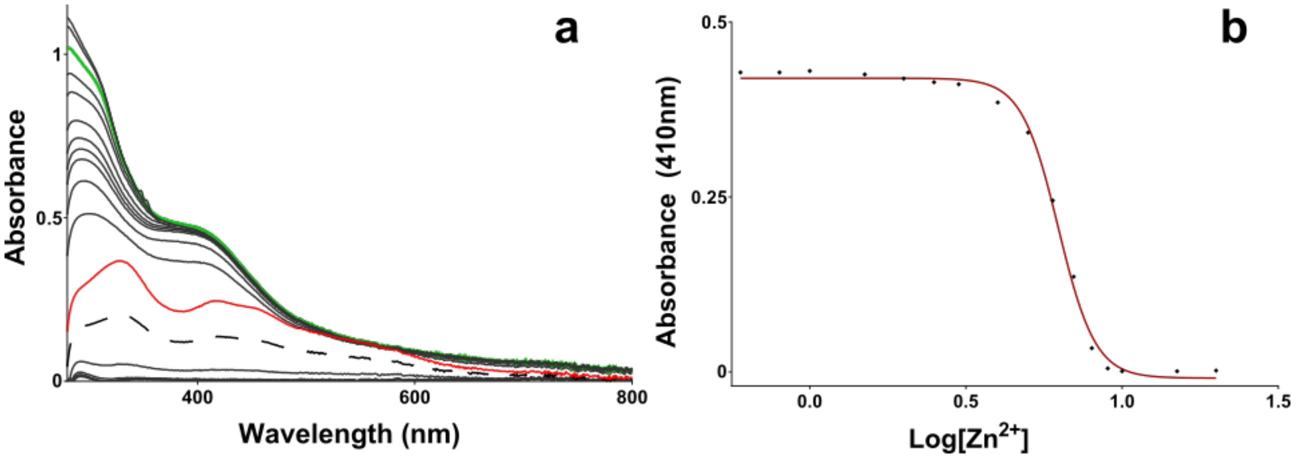

Supplementary Fig. 54. Calculation of concentration of zinc sulfate ($ZnSO_4$) needed to degrade half of $[4Fe\text{-}4S]^{2+}$ cluster concentration ($[Zn]_{50}$) in the mixture 1:1 HS^-:Fe^{3+} glutathione (40 mM glutathione, 0.5 mM Na_2S, 0.5 mM $FeCl_3$) in H_2O, pH 8.6. The Zn salt was progressively added to the solution and related UV spectra collected at each addition. a) UV-visible absorption spectra related to the Zn^{2+} titration of the solution containing $[4Fe\text{-}4S]^{2+}$ (green, solid curve, band at 410 nm) and $[2Fe\text{-}2S]^{2+}$ cluster and rubredoxin-like reduced mononuclear complex (no distinctive absorption bands) as minor species. With the increase of Zn^{2+}, the intensity of the spectra progressively decreased, and the absorption peaks shifted until clear bands indicative of $[2Fe\text{-}2S]^{2+}$ at 420 and 450 nm were observed (red, solid curve). The black dotted curve corresponds to the $[Zn^{2+}]_{50}$. b) Fitting of absorbance at 410 nm as a function of Zn^{2+} concentration; $[Zn^{2+}]_{50}$ = 6.2 mM.

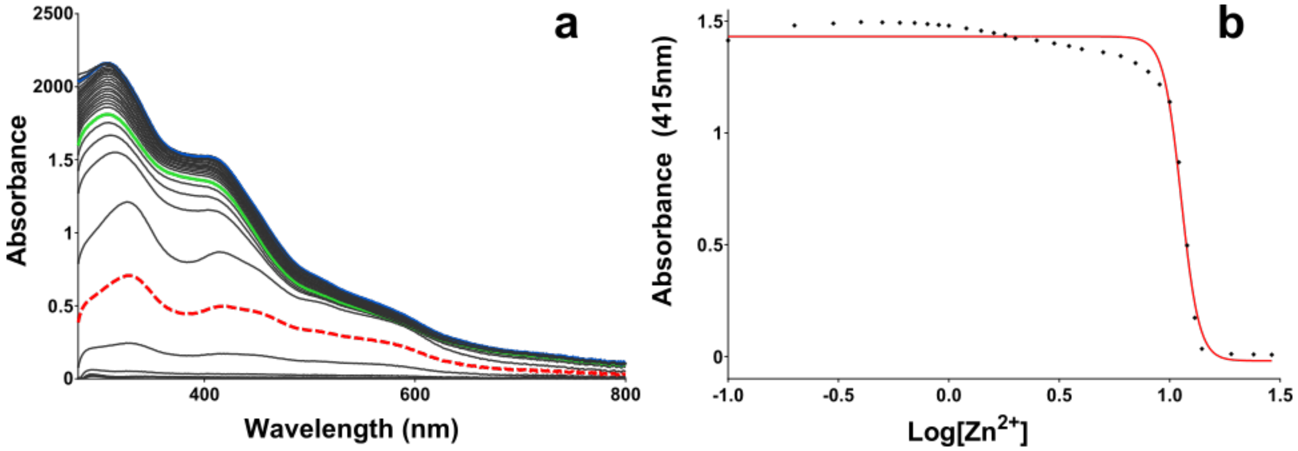

Supplementary Fig. 55. Calculation of the concentration of zinc sulfate ($ZnSO_4$) needed to degrade half of the putative $[6Fe\text{-}9S]^{2-}$ cluster concentration ($[Zn]_{50}$) in the mixture 2:1 HS^-:Fe^{3+} glutathione (40 mM glutathione, 1 mM Na_2S and 0.5 mM $FeCl_3$) in H_2O, pH 8.6. The Zn salt was progressively added to the solution and related UV spectra collected at each addition. a) UV-visible absorption spectra of the Zn^{2+} titration of a solution containing the putative $[6Fe\text{-}9S]^{2-}$ cluster (blue solid curve, band at 415 nm). As the concentration of Zn^{2+} increased, the intensity of the spectra progressively decreased and shifted until the two absorption bands indicative of a $[2Fe\text{-}2S]^{2+}$ cluster at 420 and 450 nm emerged. A $[4Fe\text{-}4S]^{2+}$-like signature (green solid curve, band at 410 nm) is recognizable before the appearance of bands indicating a $[2Fe\text{-}2S]^{2+}$ cluster (red solid curve). The red dotted spectrum, corresponding to $[2Fe\text{-}2S]^{2+}$ is at the $[Zn^{2+}]_{50}$. b) Fitting of the absorbance at 415 nm as a function of Zn^{2+} concentration; $[Zn^{2+}]_{50}$ = 11.3 mM.

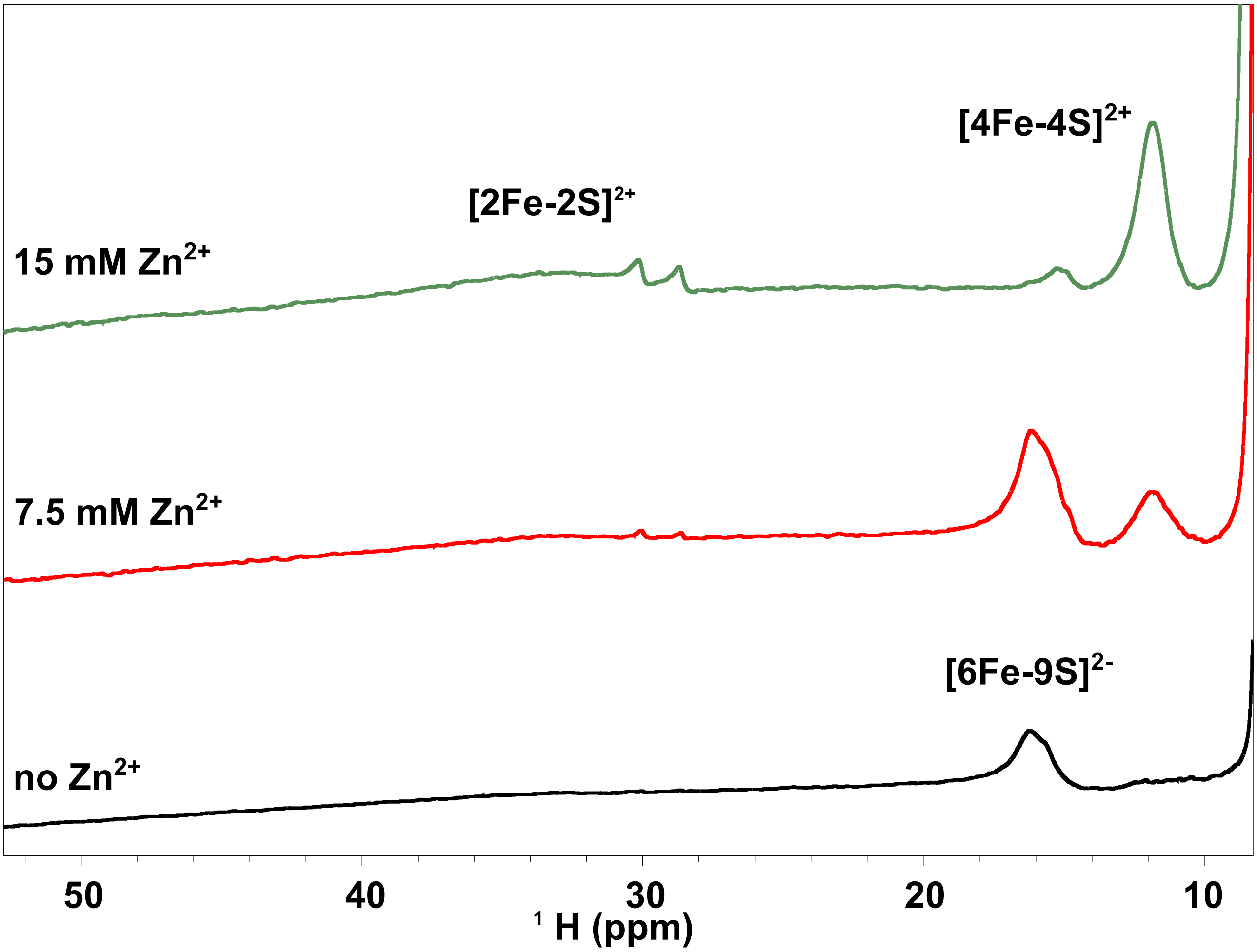

Supplementary Figure 56. ^{1}H NMR paramagnetic spectra of a mixture containing 2:1 HS^-:Fe^{3+} glutathione (150 mM glutathione, 15 mM Na_2S, 7.5 mM $FeCl_3$) in D_2O, pD 8.6 to which $ZnSO_4$ was added. Before adding zinc sulfate, the solution contained a $[6Fe\text{-}9S]^{2-}$ cluster (16 ppm, black, bottom). When 7.5 mM Zn^{2+} was added, the resonance at 16 ppm broadened and a resonance at 11.8, 28 and 30 ppm (indicative of a $[4Fe\text{-}4S]^{2+}$ cluster) emerged (red, middle). Furthermore, a faint, broad resonance at 33 ppm ($[2Fe\text{-}2S]^{2+}$) was detected. At 15 mM Zn^{2+}, the $[6Fe\text{-}9S]^{2-}$ cluster is lost, the resonances of $[4Fe\text{-}4S]^{2+}$ increased and small amounts of $[2Fe\text{-}2S]^{2+}$ are observed. The data are consistent with that observed by UV-visible absorption spectroscopy for Zn^{2+} titrations of a putative $[6Fe\text{-}9S]^{2+}$ cluster (**Fig. S55**). In the process, no resonance related to mononuclear complex (211 ppm) could be detected.

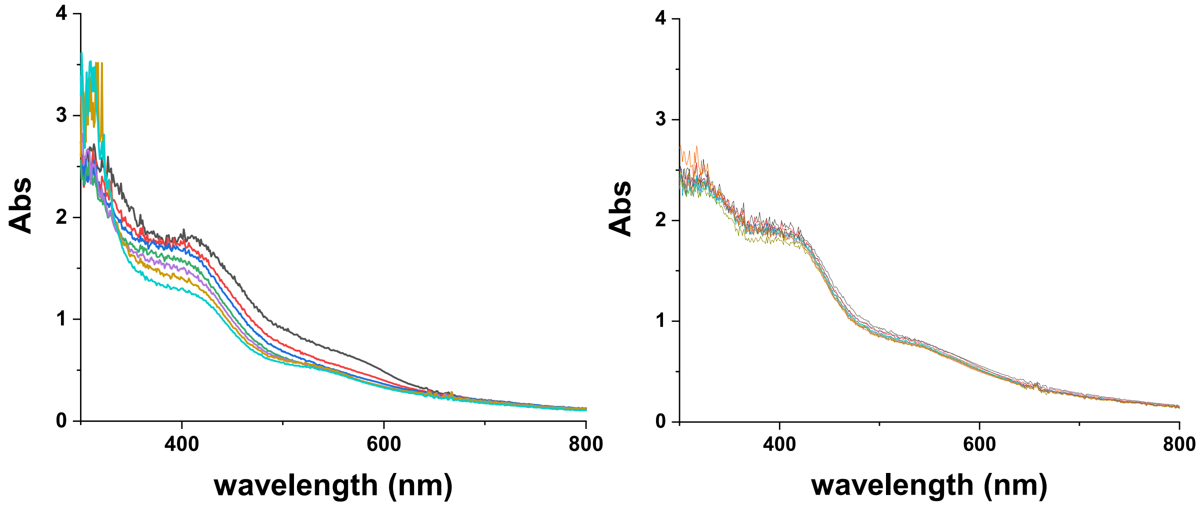

Supplementary Figure 57. Comparison of UV-visible absorption spectra of mixtures containing $[4Fe\text{-}4S]^{2+}$ glutathione (left, 40 mM glutathione, 0.5 mM Na_2S, 0.5 mM $FeCl_3$) and putative $[6Fe\text{-}9S]^{2-}$ glutathione (right, 40 mM glutathione, 1 mM Na_2S, 0.5 mM $FeCl_3$) progressively collected over the time (time-points at 1, 60, 120, 240, 480, 960 and 1350 minutes, respectively). The spectra show, for $[4Fe\text{-}4S]^{2+}$ glutathione, a progressive decrease of the signal intensity, suggesting a slow degradation of the cluster core occurring over the time. At the end of the experiment (about 24 hours) it is possible to observe a black precipitate, attributable to precipitation of iron sulfide species, in the cuvette. Conversely, over the same period of time, the spectra collected for $[6Fe\text{-}9S]^{2-}$ glutathione show small or negligible variations in both curve profiles and intensity, suggesting a more pronounced stability of the higher nuclearity cluster core with respect to the $[4Fe\text{-}4S]^{2+}$. After 24 hours no precipitate was observed in the cuvette.

Supplemental references

1 M. Carboni, M. Clémancey, F. Molton, J. Pécaut, C. Lebrun, L. Dubois, G. Blondin and J.-M. Latour, *Inorg. Chem.*, 2012, **51**, 10447–10460.

2 M. Carboni, M. Clémancey, F. Molton, J. Pécaut, C. Lebrun, L. Dubois, G. Blondin and J.-M. Latour, *Inorg. Chem.*, 2012, **51**, 12053–12053.

3 C. Charavay, S. Segard, F. Edon, M. Clemancey and G. Blondin, SimuMoss Software Univ.. Grenoble Alpes, CEA/BIG, CNRS, Grenoble 2012.

4 G. Battistuzzi, M. Borsari, M. Sola and F. Francia, *Biochemistry*, 1997, **36**, 16247–16258.

5 J. Meyer, M. D. Clay, M. K. Johnson, A. Stubna, E. Münck, C. Higgins and P. Wittung-Stafshede, *Biochemistry*, 2002, **41**, 3096–3108.

6 H.-C. Zhou, W. Su, C. Achim, P. V. Rao and R. H. Holm, *Inorg. Chem.*, 2002, **41**, 3191–3201.

7 I. Bertini and C. Luchinat, *NMR of Paramagnetic Substances*, Elsevier, 1996.

8 M. Y. Hamed, J. Silver and M. T. Wilson, *Inorganica Chim. Acta*, 1983, **78**, 1–11.

9 M. Y. Hamed and J. Silver, *Inorganica Chim. Acta*, 1983, **80**, 115–122.

10 J. Silver, M. Y. Hamed and I. E. G. Morrison, *Inorganica Chim. Acta*, 1985, **107**, 169–178.

11 K. S. Hagen, A. D. Watson and R. H. Holm, *J. Am. Chem. Soc.*, 1983, **105**, 3905–3913.

12 J. Liu, S. Chakraborty, P. Hosseinzadeh, Y. Yu, S. Tian, I. Petrik, A. Bhagi and Y. Lu, *Chem. Rev.*, 2014, **114**, 4366–4469.

13 I. Salmeen and G. Palmer, *Arch. Biochem. Biophys.*, 1972, **150**, 767–773.

14 K. Nagayama, Y. Ozaki, Y. Kyogoku, T. Hase and H. Matsubara, *J. Biochem. (Tokyo)*, 1983, **94**, 893–902.

15 S. Ueno, N. Ueyama, A. Nakamura and T. Tukihara, *Inorg. Chem.*, 1986, **25**, 1000–1005.
